# 3-Substituted Blatter Radicals: Cyclization of *N*-Arylguanidines and *N*-Arylamidines to Benzo[*e*][1,2,4]triazines and PhLi Addition

**DOI:** 10.1021/acs.joc.2c02703

**Published:** 2023-02-17

**Authors:** Dominika Pomikło, Agnieszka Bodzioch, Piotr Kaszyński

**Affiliations:** † Centre of Molecular and Macromolecular Studies, Polish Academy of Sciences, 90-363 Łódź, Poland; ‡ Faculty of Chemistry, University of Łódź, 91-403 Łódź, Poland; § Department of Chemistry, Middle Tennessee State University, 37132 Murfreesboro, Tennessee, United States

## Abstract

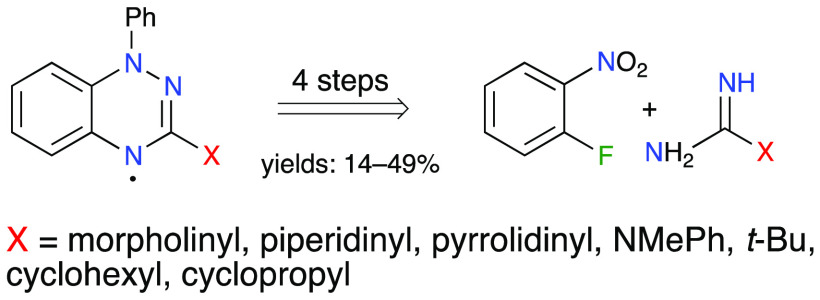

A series of 3-amino- and 3-alkyl-substituted 1-phenyl-1,4-dihydrobenzo[*e*][1,2,4]triazin-4-yls was prepared in four steps involving N-arylation, cyclization of *N*-arylguanidines and *N*-arylamidines, reduction of the resulting *N*-oxides to benzo[*e*][1,2,4]triazines, and subsequent addition of PhLi followed by aerial oxidation. The resulting seven C(3)-substituted benzo[*e*][1,2,4]triazin-4-yls were analyzed by spectroscopic and electrochemical methods augmented with density functional theory (DFT) methods. Electrochemical data were compared to DFT results and correlated with substituent parameters.

## Introduction

**Figure 1:**
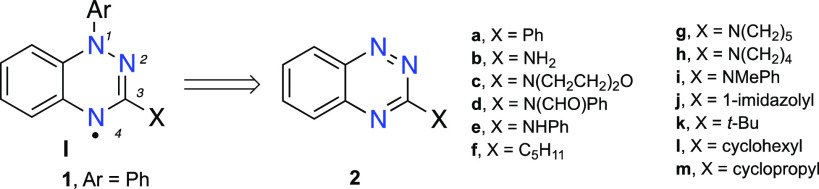
Preparation of Blatter radicals **I** by azaphilic addition of ArLi to benzo[*e*][1,2,4]triazines **2**.

Benzo[*e*][1,2,4]triazin-4-yls **I**,^1−3^ derivatives of the prototypical Blatter radical^4^ (**1a**, X = Ph, Figure 1), are increasingly important elements of advanced materials investigated in the context of controlled polymerization,^5^ organic batteries,^6−8^ photoconductive liquid crystals,^9−14^ surface functionalization,^15^ molecular electronics,^16^ sensory,^17,18^ and spintronic^19^ applications. These investigations have stimulated advancement in chemistry of the benzo[*e*][1,2,4]triazinyls^20−22^ and preparation of materials with tailored properties. One of the methods for the synthesis of Blatter radical derivatives of the general structure **I** involves azaphilic addition of ArLi to benzo[*e*][1,2,4]triazines **2** (Figure 1).^23^ This method permitted the preparation of paramagnetic liquid crystals^9−13^ and C(3) functional derivatives of Blatter radical, including the 3-amino **1b** and 3-(morpholin-4-yl) **1c**.^24^ Another derivative, containing substituent X = N(CHO)Ph at the C(3) position (**1d**), was obtained by a rearrangement of a stable carbene and hydrolyzed to **1e** (X = NHPh).^25^

An analysis of literature data indicates that the 3-amino substituent in benzo[*e*][1,2,4]triazin-4-yl derivatives is particularly effective in the modification of electronic properties of the radicals: it effects a significant cathodic shift of the oxidation potential and a bathochromic shift in the electronic absorption, relative to the prototypical Blatter radical **1a**.^24^ Similar, although less pronounced effects, were observed for the 3-pentyl derivative **1f**.^24^ For these reasons, 3-amino and 3-alkyl derivatives **1** are of interest for the fine-tuning of electronic properties of the benzo[*e*][1,2,4]triazinyl system and also in the context of our program in self-organizing paramagnetic materials^9−13^ with controlled photophysical and redox behavior. In addition, 3-aminobenzo[*e*][1,2,4]triazines, direct precursors to the radicals, have been demonstrated to possess antimalarial,^26^ antitumor,^27,28^ and *Abl* enzyme-inhibiting^29^ activities, while their 1,4-dioxides are bioreductive antitumor agents with selective toxicity to oxygen-deprived (hypoxic) cells.^30−32^

**Figure 2:**
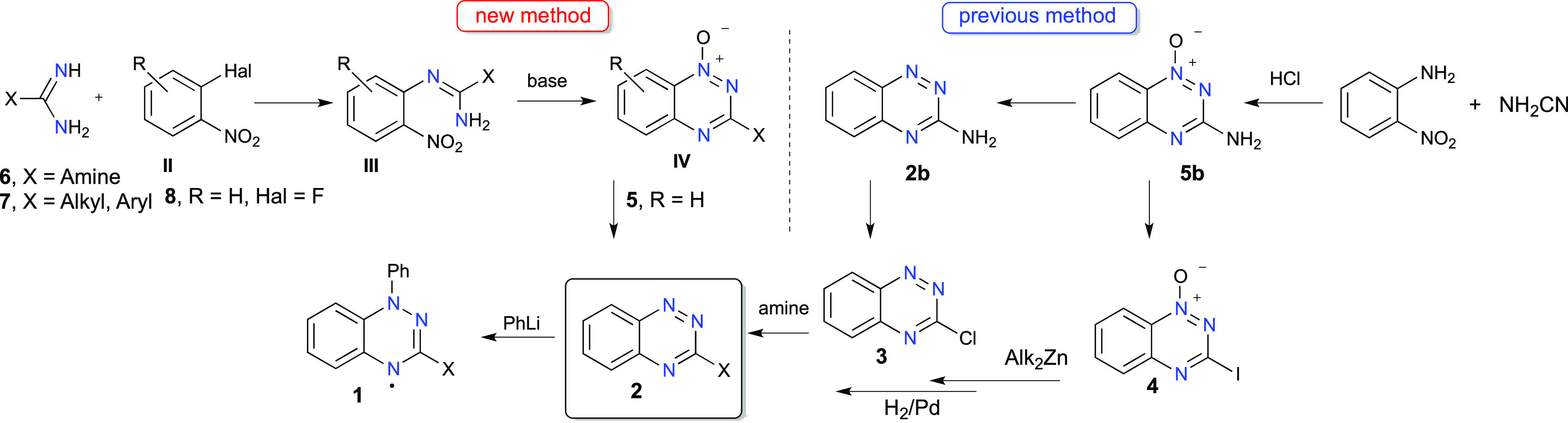
A comparison of two general strategies for the formation 3-substituted benzo[*e*][1,2,4]triazines **2**, precursors to radicals **1**.

The existing methods^24,25^ for the preparation of 3-amino and 3-alkyl derivatives **I** rely mainly on benzo[*e*][1,2,4]triazines **2**.^33,34^ The requisite amines **2c** and **2e** were obtained from 3-chlorobenzo[*e*][1,2,4]triazine (**3**, Figure 2), while the 3-pentyl derivative **2f** was prepared in two steps from 3-iodobenzo[*e*][1,2,4]triazine-1-oxide (**4**).^33^ Although the two halo derivatives **3** and **4** are general intermediates to a variety of such C(3)-substituted radicals,^24^ their synthesis is a multistep process and involves poorly soluble intermediates, e.g., **5b**,^33,35^ which is problematic for the preparation of polyradicals and more complex molecular systems. Therefore, in search for an alternative, more direct, and convenient method for the preparation of **2**, we focused on N-substituted guanidines **6** and amidines **7** as the starting materials. We have envisioned that their N-arylation with 1-fluoro-2-nitrobenzene (**8**) followed by cyclization could lead to the desired benzo[*e*][1,2,4]triazines **2** with the amino substituent of guanidine **6** and the alkyl residue of **7** incorporated at the C(3) position.

A literature search revealed that there are limited examples of N-arylation of the parent guanidine^35−39^**6** (X = NH_2_) and amidines **7** (X = Me, Ph, RC_6_H_4_)^40^ with 2-halonitroarenes **II**, via the S_N_Ar mechanism, and formation of the substitution products **III** (Figure 2). In the absence of the activating NO_2_ group, N-arylations of **6** and **7** are typically accomplished using Ullmann-type conditions (base and CuI).^41−45^ Treatment of *N*-(2-nitroaryl)guanidine derivatives **III** (X = amine) with bases, such as NaOH, *t*-BuOK, or *t*-BuOLi, leads to 3-aminoareno[*e*][1,2,4]triazine-1-oxides **IV** (Figure 2).^30,35,38,46−49^ The two processes, N-arylation and base-induced cyclization, are often combined into a one-pot reaction, and areno[1,2,4]triazine-1-oxides **IV** are isolated in good yields.^30^ The analogous cyclization of *N*-(2-nitrophenyl)amidines **III** (X = alkyl, aryl) in the presence of MeONa/MeOH was reported to lead also to benzo[*e*][1,2,4]triazine-1-oxides **IV**.^50^ The substitution–cyclization tandem working for 2-halonitroarenes was different for reactions of nitronaphthalenes and nitroquinolines with guanidine and two amidines **7** (X = Ph, Me), for which a sequence of addition-oxidation-cyclization-deoxygenation was postulated as a one-pot process.^49^

Guanidines and amidines are often difficult to work with as reagents. Free guanidines are highly basic,^51^ rapidly absorbing carbon dioxide and moisture and, like amidines, are thermally unstable undergoing decomposition with a release of ammonia.^30,49^ On the other hand, the guanidine functionality has been found in many natural products and pharmaceuticals, playing key roles in various biological functions.^52−54^ Guanidine derivatives serve also as nucleophilic catalysts,^53,55^ auxiliaries in asymmetric synthesis,^56^ precursors for the synthesis of heterocycles,^45,56^ anion recognition, and as ligands for metal complexes and clusters.^54^

Herein, we explore the synthetic access to four C(3)-amino (X = morpholin-4-yl **c**, piperidin-1-yl **g**, pyrrolidin-1-yl **h**, NMePh **i**, and imidazol-1-yl **j**, Figure 1) and three C(3)-alkyl (X = *t*-Bu **k**, cyclohexyl **l**, and cylopropyl **m**) benzo[*e*][1,2,4]triazines **2** by nucleophilic aromatic guanidinylation and amidinylation, respectively, of 1-fluoro-2-nitrobenzene (**8**). The cyclization of the corresponding *N*-arylguanidines and *N*-arylamidines gave a series of *N*-oxides **5**, which were deoxygenated. The resulting benzo[*e*][1,2,4]triazines **2** were converted by azaphilic addition of PhLi to radicals **1**, which were investigated by spectroscopic and electrochemical methods. The experimental data were compared with density functional theory (DFT) computational results and substituent parameters.

## Results and Discussion

### Preparation of Guanidines 6

**Scheme 1:**
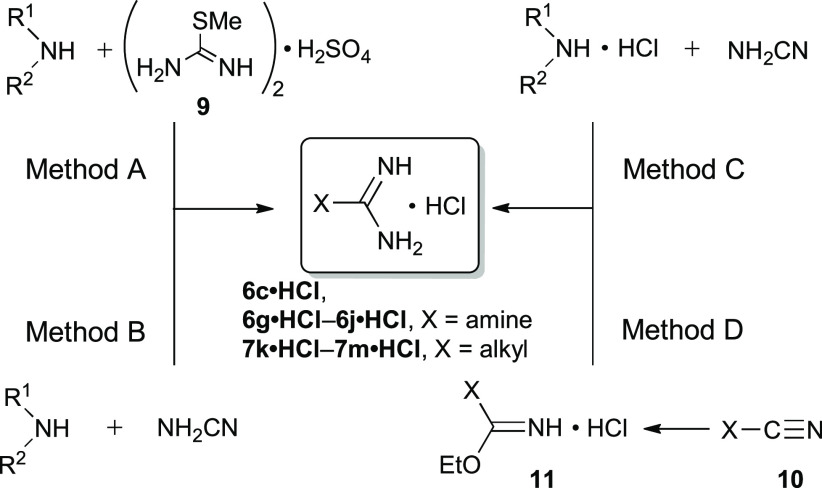
Preparation of Guanidine (6·HCl) and Amidine (7·HCl) Hydrochlorides *Reagents and conditions*: Method A: (1) H_2_O, reflux, overnight; (2) BaCl_2_, reflux, 1h. Method B: HCl, EtOH, reflux, overnight. Method C: pH 8–9, H_2_O, reflux, overnight. Method D: (1) HCl, EtOH, 0 °C, overnight; (2) dry EtOH, NH_3_ gas, rt, overnight.

Classical syntheses of guanidines involve mainly cyanamides, carbodiimides, thiourea, and isocyanide-based precursors or guanylating reagents, such as *S*-methylisothioureas, pyrazole-1-carboximidamide and its derivatives, or triflyl guanidines.^57,58^ In spite of a variety of known methods, most of them involve harmful precursors or harsh reaction conditions. For our purpose, substituted guanidines **6** were obtained as hydrochorides **6·HCl** using relatively safe reactions of amines with commercially available *S*-methylisothiourea guanylation agent **9** (Scheme 1, Method A). Thus, following the literature procedure,^59^ reaction of 2-methyl-2-thiopseudourea sulfate (**9**) with morpholine proceeded smoothly giving the desired morpholine-4-carboximidamide hydrochloride (**6c·HCl**) in 90% yield after 2 h. Contrary to the literature report,^59^ the synthesis of piperidine-1-carboximidamide hydrochloride (**6g·HCl**) required significant elongation of the reaction time, and even after 48 h an inseparable mixture of the desired guanidine hydrochloride **6g·HCl** and piperidine hydrochloride was obtained. Therefore, an additional amount (1 equiv) of 2-methyl-2-thiopseudourea sulfate (**9**) was added, and the reaction was conducted for another 24 h giving the complete transformation. A similar result was obtained in reaction of **9** with *N*-methylaniline. Therefore, a strategy involving reaction of the amine with cyanamide in the presence of HCl was explored (Scheme 1, Method B).^60^ Thus, a reaction of cyanamide with morpholine and *N*-methylaniline gave the corresponding guanidine hydrochlorides **6c·HCl** and **6i·HCl** in 75% and 74% yields, respectively. On the other hand, attempts at a synthesis of piperidine-1-carboximidamide hydrochloride (**6g·HCl**) gave only piperidine hydrochloride under these conditions. Finally, condensing piperidine hydrochloride with cyanamide in a buffered solution (pH = 8–9) consisting of piperidine hydrochloride and piperidine provided **6g·HCl** in 91% yield (Scheme 1, Method C).^61^ The same strategy was used for the preparation of guanidine hydrochlorides containing pyrrolidin-1-yl (**6h·HCl**) and imidazol-1-yl (**6j·HCl**) substituents in 86% and 48% yields, respectively.

### Preparation of Amidines 7

Amidine hydrochlorides containing *t*-Bu (**7k·HCl)** and *c*-Hex (**7l·HCl)** substituents were obtained via the Pinner reaction.^57^ Thus, an acid-induced reaction of the appropriate nitrile **10** with dry EtOH resulted in the formation of imino ester salts **11·HCl**, which were reacted with ammonia to form the desired amidine hydrochlorides **7k·HCl** and **7l·HCl** in 81% and 90% yields, respectively (Scheme 1, Method D). Cyclopropanecarboxamidine hydrochloride (**7m·HCl**) was commercially available.

### *N*-Arylation and Cyclization to Benzo[*e*][1,2,4]triazine-1-oxides 5

N-Substituted guanidine hydrochlorides **6·HCl** and amidine hydrochlorides **7·HCl** were used as key substrates for the synthesis of C(3)-amino and C(3)-alkyl derivatives of Blatter radicals **1** via a three-step procedure involving: (1) nucleophilic aromatic substitution of 1-fluoro-2-nitrobenzene (**8**) with free guanidine **6** or amidine **7** followed by base-induced cyclization,^30,38^ (2) reduction of *N*-oxides **5**, and, finally (3) addition of PhLi to the obtained benzo[*e*][1,24]triazines **2** (Figure 2).

**Scheme 2:**
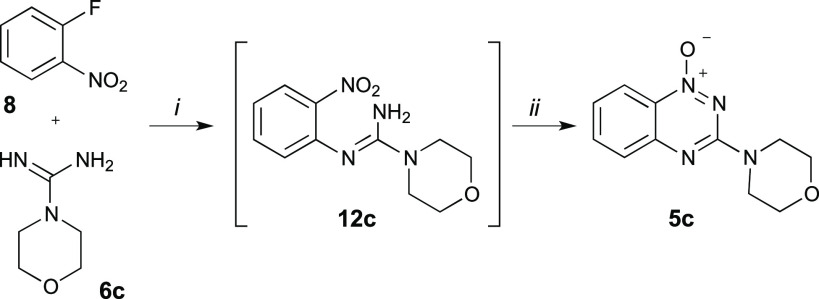
Optimized Reaction Conditions for Synthesis of *N*-Oxide 5c *Reagents and conditions*: (*i*) **8**, MeCN, 78 °C, overnight; (*ii*) *t*-BuOK, MeCN, 78 °C, 3 h.

Initial experiments involved a reaction of **8** with morpholine-4-carboximidamide (**6c**), which was liberated from **6c·HCl** using equivalent amounts of EtONa in EtOH. The strong basicity of guanidine derivatives considerably limits the type of solvent, which can be used for this reaction. Following a literature report,^30^ tetrahydrofuran (THF) was selected as the solvent for this reaction. However, due to low solubility of free guanidine **6c** in THF at both ambient and elevated temperatures, 25% v/v of dimethyl sulfoxide (DMSO) was added to the reaction mixture. After 12 h at 70 °C thin layer chromatography (TLC) showed a highly polar product suggesting the formation of substitution product **12c** (Scheme 2), which was accompanied by unreacted 1-fluoro-2-nitrobenzene (**8**). Addition of 1.5 equiv of *t*-BuOK initialized the cyclization reaction; however, after 3 h at 70 °C, TLC still showed the unreacted substitution product **12c**. Therefore, additional amounts of *t*-BuOK (1.5 equiv) were added, and the reaction time was extend for another 12 h giving the desired 3-(morpholin-4-yl)benzo[*e*][1,2,4]triazine-1-oxide (**5c**) in an overall yield of 65% (Scheme 2). A similar strategy was applied for substitution of 1-fluoro-2-nitrobenzene (**8**) with other guanidine derivatives containing piperidine and *N*-methylaniline moiety providing the corresponding *N*-oxides **5g** and **5i** in 25% and 42% yields, respectively.

**Scheme 3:**
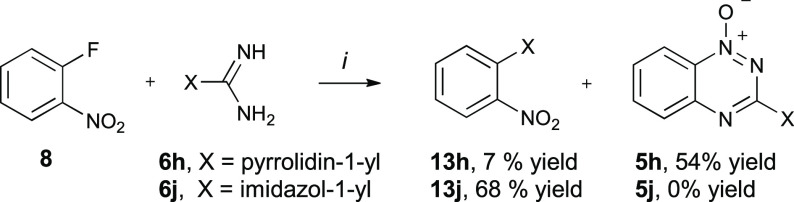
Reaction of 8 with Guanidines 6h and 6j *Reagents and conditions*: (*i*) **8**, MeCN, 78 °C, overnight.

In all cases TLC analysis showed the presence of unreacted 1-fluoro-2-nitrobenzene (**8**) after the substitution step. Therefore, to improve conversion of **8**, 6 equiv of guanidine **6** was used. In addition, the solvent mixture (THF/DMSO) was replaced with MeCN to simplify the reaction workup procedure. Under these conditions, the nucleophilic aromatic guanidinylation of **8** with morpholine guanidine **6c** showed full conversion of **8** to **12c** (TLC analysis), which after *t*-BuOK-promoted cyclization, provided *N*-oxide **5c** in 72% yield (Table 1). Following this one-pot procedure, substitution of **8** with piperidin-1-yl (**6g**) and *N*-methyl-*N*-phenyl (**6i**) guanidines gave the corresponding *N*-oxides **5** in 54% and 63% yields, respectively. Surprisingly, the reaction of **8** with pyrrolidine-1-carboxamidine (**6h**) proceeded smoothly providing the desired *N*-oxide **5h** in 70% yield during the substitution step (Table 1) without the need of *t*-BuOK. The formation of **5h** was accompanied by **13h** as a substitution product of the fluorine atom with pyrrolidine (Scheme 3). Under the reaction conditions, imidazole guanidine **6j** underwent a complete decomposition to imidazole, which reacted with 1-fluoro-2-nitrobenzene (**8**) giving **13j** as the substitution product isolated in 68% yield (Scheme 3). Subsequent catalytic (Pd/C) hydrogenation of *N*-oxides **5** in EtOH/AcOEt gave 3-aminobenzo[*e*][1,2,4]triazines **2** in nearly quantitative yields (Figure 2, Table 1).

**Table 1:** Synthesis of Radicals 1

Guanidine **6**/ amidine**7**	*N*-Oxide **5** (yield %)^a^	Benzotriazine **2** (yield %)	Radical **1** (yield %)
**c**, X = morpholin-4-yl	72^b^	99	67
**g**, X = piperidin-1-yl	54^b^	99	86
**h**, X = pyrrolidin-1-yl	70^c^	95	72
**i**, X = NMePh	63^b^	99	73
**k**, X = *t*-Bu	25^d^	99	76
**l**, X = cyclohexyl	22^d^	99	79
**m**, X = cyclopropyl	17^d^	99	84

a Isolated yields obtained for optimized reaction conditions.

b *t*-BuOK promoted cyclization.

c Without *t*-BuOK.

d Two-step procedure with MeONa-promoted cyclization.

**Scheme 4:**
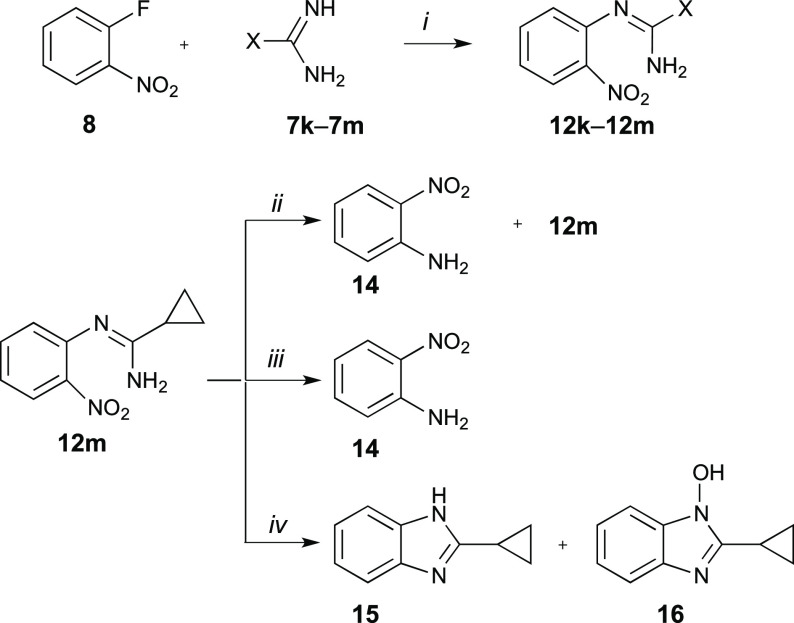
Nucleophilic Aromatic Substitution of 1-Fluoro-2-nitrobenzene (8) with Amidines 7k–7m and Attempts at Cyclization of 12m *Reagents and conditions*: (*i*) **8**, MeCN, 70 °C, overnight; (*ii*) 10% NaOH, EtOH, 78 °C, overnight; (*iii*) cat. HCl, EtOH, 78 °C, overnight; (*iv*) H_2_, Pd/C, EtOH, rt, overnight.

The methodology developed for the synthesis of 3-amino derivatives **2** was extended to conversion of amidines **7** to 3-alkyl substituted benzo[*e*][1,2,4]triazines **2**. The formation of amidine-substituted products **12k**–**12m** was observed by TLC during the reaction of amidines **7** with **8**; however, cyclization under the previously applied conditions (*t*-BuOK) gave complex mixtures of products without formation of the desired *N*-oxides **5k**–**5m**. Therefore, the arylation products **12k**–**12m** were isolated (yields 88–94%) from the reaction of 1-fluoro-2-nitrobenzene (**8**) with amidines **7k**–**7m**, and several cyclization reactions were tested (Scheme 4). Thus, a reaction of **12m** with 10% NaOH^46^ or with catalytic amounts of HCl in EtOH gave only the unreacted substrate **12m** after overnight stirring at room temperature. Increasing the temperature to 60 °C resulted in the formation of trace amounts of 2-nitroaniline (**14**), which accompanied the unreacted **12m**. Finally, overnight reflux of an ethanolic solution of **12m** with 10% NaOH gave 16% of **14** (based on ^1^H NMR), while the same with catalytic amounts of HCl gave **14** in 72% yield (based on ^1^H NMR) (Scheme 4). Formation of small amounts of 2-nitroaniline (**14**) was also observed for **12m** in refluxing EtOH. The same results were obtained for reactions of **12l** and **12k** with NaOH and HCl in EtOH. On the other hand, an attempted transformation of **12m** to the corresponding *N*-oxide under reductive conditions (Pd/C, H_2_) resulted in cyclization to benzimidazole derivatives **15** and **16** isolated in 16% and 82% yields, respectively (Scheme 4).

Due to the lack of success in the cyclization of **12m** to *N*-oxide **5m** using typical conditions, it was decided to use MeONa as the cyclization-promoting reagent previously reported for the synthesis of 3-phenylbenzo[*e*][1,2,4]triazine-1-oxide (**5a**).^50^ In our hands a one-pot synthesis of *N*-oxide **5a**, involving N-arylation of **8** with amidine **7a** in MeOH and subsequent treatment with MeONa, provided the desired product **5a** in 61% yield. A similar strategy applied in the synthesis of **5k**–**5m** resulted in the formation of 2-nitroanisole as the main product. To avoid this undesired process, it was decided to follow a two-step procedure with the isolation of substitution products **12k**–**12m**. Thus, cyclization of the isolated **12k**–**12m** using 1.5 equiv of MeONa in MeOH gave the corresponding *N*-oxides **5k**–**5m** in 17–25% yields (Table 1).

### Preparation of C(3)-Substituted Radicals 1

3-Amino- and 3-alkyl substituted benzo[*e*][1,2,4]triazines **2** were reacted with PhLi (Figure 2) giving the desired radicals **1** in good yields (Table 1). In comparison to morpholine derivative **1c**,^24^ radicals **1g**–**1i** were unstable during purification by column chromatography (Et_3_N passivated silica gel, neutral aluminum oxide, or neutral Florosil) and underwent fast decomposition to highly polar purple products, presumably iminoquinone type,^62,63^ which could not be eluted from the column. Therefore, radicals **1g**–**1i** were purified by passing the crude mixture through a short diatomaceous earth pad (Cellite), washing the residue with *n*-pentane, and finally recrystallization from *n*-heptane. A similar purification process was used to obtain pure 3-alkyl-substituted radicals **1k**–**1m**. Following this procedure, radicals **1g**–**1i** and **1k**–**1m** were obtained in 67–86% yields from **2** (Table 1). All newly prepared radicals are solids except for cyclohexyl and cyclopropyl derivatives **1l** and **1m**, which are liquids and thus slowly decompose on standing.

### Characterization of Radicals 1

**Figure 3:**
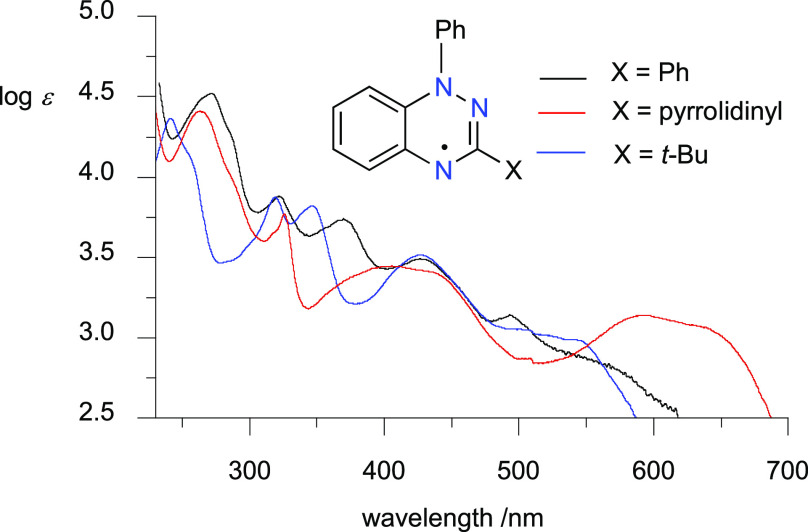
UV–Vis spectra for Blatter **1a** (black), 3-pyrrolidinyl **1h** (red), and 3-*t*-Bu **1k** (blue) radicals in CH_2_Cl_2_.

**Figure 4:**
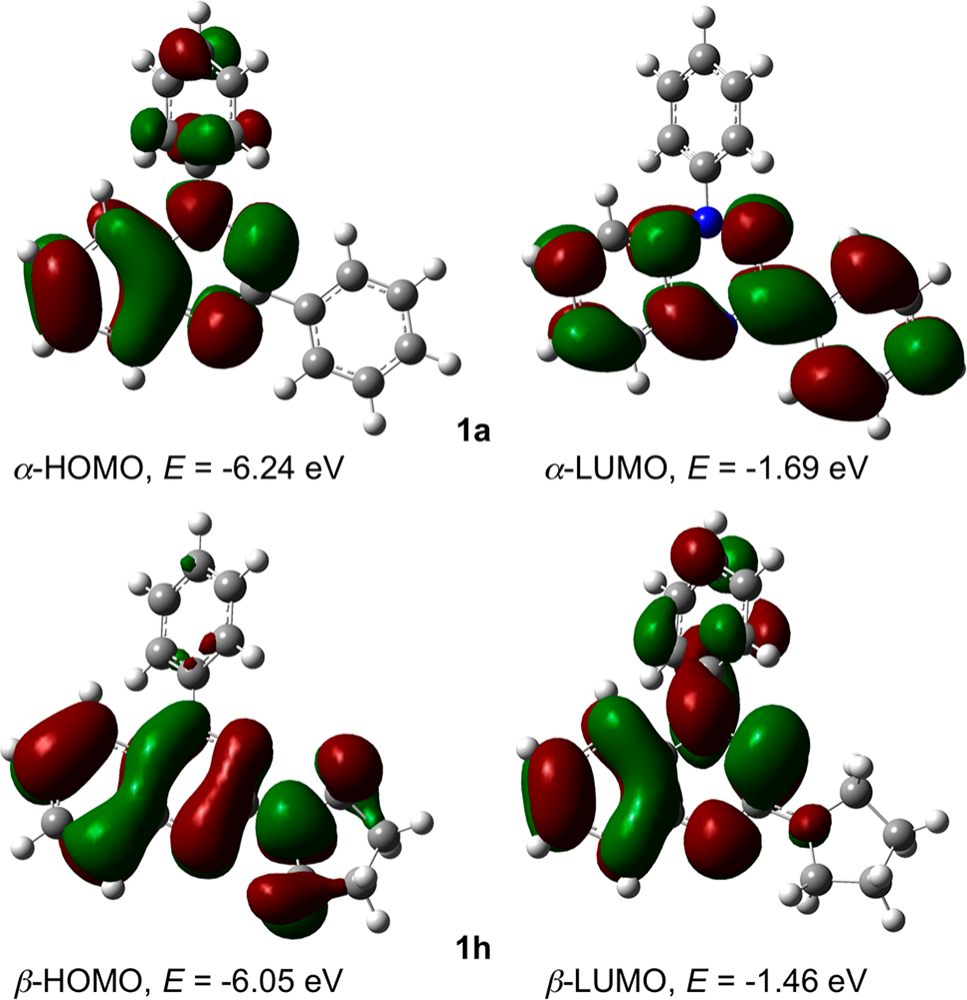
TD UCAM-B3LYP/6-31++G(2d,p)//UB3LYP/6-31G(2d,p) derived contours (isovalue = 0.02) and energies of molecular orbitals in CH_2_Cl_2_ dielectric medium relevant to the lowest-energy excitations in **1a** and **1h**.

Analysis of the radicals in series **1** revealed the effects of the C(3) substituent on the spectroscopic and electrochemical properties. Thus, all radicals exhibit broad, low-intensity absorption bands in the visible range up to 700 nm for C(3)-amino derivatives **1c** and **1g**–**1i** and up to 600 nm for C(3)-alkyl derivatives **1k**–**1m**, with poorly defined absorption maxima. The most pronounced bathochromic effect on the absorption spectrum is exhibited by the 3-pyrrolidinyl derivative **1h** (Figure 3). The observed trend in excitation energies (Table 2) is well-reproduced computationally. A time-dependent density functional theory (TD DFT) analysis indicates that the lowest-energy excitation calculated at about 500 nm for C(3)-amino radicals **1c** and **1g**–**1i** is of the π–π* type involving the β-HOMO → β-LUMO transition (HOMO = highest occupied molecular orbital; LUMO = lowest unoccupied molecular orbital), while for C(3)-phenyl radical **1a** it involves mainly the α-HOMO → α-LUMO transition (Figure 4). On the other hand, a TD DFT analysis of C(3)-alkyl derivatives **1k**–**1m** suggests comparable contributions of α-HOMO → α-LUMO and β-HOMO → β-LUMO transitions to the lowest-energy excitations calculated at about 460 nm. The difference in the origin of the lowest-energy excitation is due to destabilization of the β-HOMO by the amine lone pair and consequent narrowing of the β-HOMO-β-LUMO energy gap (Figure 4 and Supporting Information). Thus, the energy of the β-HOMO in amines is up to 0.7 eV higher than that in the prototypical **1a**.

**Table 2:** Selected Experimental and Calculated Electronic Parameters for C(3)-Substituted Benzo[e][1,2,4]triazin-4-yls 1

radical	λ_max_ exp^a^ /nm	λ_max_ theor^b^ /nm	*Ε*_α-HOMO_^b^ /eV	*Ε*_β-LUMO_^b^ /eV	*E*1/2–1/0^c^ /V	*E*1/2^0/ + 1^^c^ /V	*E*_cell_^d^ /V	*a*_N(1)_^e^ /G	*a*_N(2)_^e^ /G	*a*_N(4)_^e^ /G
**1a**^f^	492	516	–6.240	–1.690	–0.92	0.28	1.20	7.65	4.87	4.90
**1b**^f^	565	478	–6.198	–1.607	–0.956	0.150	1.106	7.96	4.24	5.71
**1c**^f^	584	514	–6.127	–1.530	–0.981	0.083	1.064	7.99	4.13	5.75
**1g**	593	531	–6.060	–1.466	–1.001	0.021	1.022	7.79	4.09	5.88
**1h**	595	534	–6.047	–1.457	–1.021	0.012	1.033	7.83	4.07	5.93
**1i**	590	514	–6.128	–1.535	–0.921	0.088	1.009	7.78	4.21	5.72
**1k**	545	462	–6.171	–1.623	(–1.028)^g^	0.218	–	7.46	4.82	5.34
**1l**	545	462	–6.192	–1.639	(–0.994)^g^	0.218	–	7.54	5.02	5.02
**1m**	548	466	–6.196	–1.650	(–1.008)^g^	0.218	–	7.52	4.96	4.96

a The lowest-energy absorption band recorded in CH_2_Cl_2_.

b Obtained at the TD UCAM-B3LYP/6-31++G(2d,p)//UB3LYP/6-31G(2d,p) level of theory in CH_2_Cl_2_ dielectric medium.

c Potentials vs Fc/Fc^+^ couple (0.46 V vs SCE).^64^ Recorded in CH_2_Cl_2_ with [*n-*Bu_4_N]^+^[PF_6_]^−^ (50 mM), at ca. 20 °C, 50 mV s^–1^, glassy carbon working electrode (2 mm disc). For details see the Supporting Information.

d *E*_cell_ = *E*_1/2_^0/+1^ – *E*_1/2_^–1/0^.

e Recorded in benzene at ca. 20 °C.

f Ref (24).

g Cathodic potential for irreversible reduction process.

**Figure 5:**
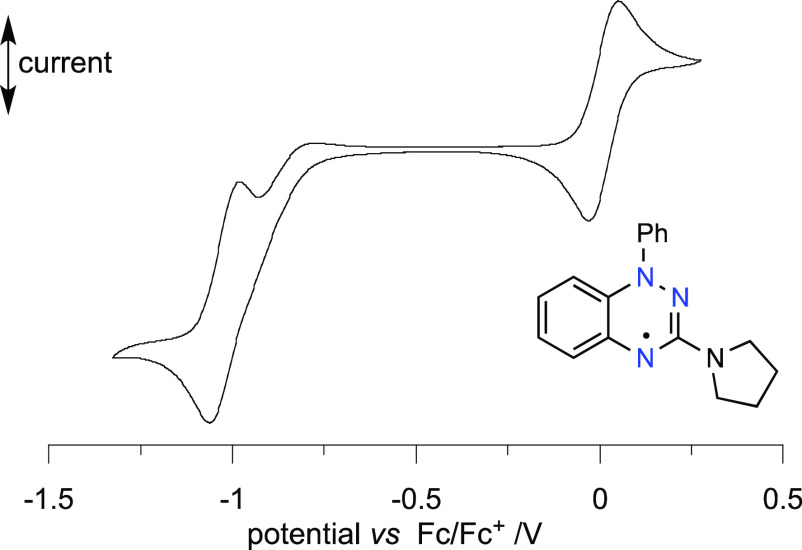
Cyclic voltammograms for **1h** (0.5 mM) in CH_2_Cl_2_ [*n-*Bu_4_N]^+^[PF_6_]^−^ (50 mM) vs Fc/Fc^+^, ca. 20 °C, 50 mV s^–1^, glassy carbon electrode (2 mm disc), scan starting at 0 V in the anodic direction. For details see the Supporting Information.

Results of electrochemical analysis of radicals **1g**–**1i** are generally consistent with those previously obtained for morpholine derivative **1c**,^24^ showing quasi-reversible oxidation and reduction processes. The only exception are two radicals with the most basic substituents, piperidinyl **1g** and pyrrolidinyl **1h**, for which reduction is a complex, presumably 2e^–^ process involving a chemical step, such as protonation (Figure 5). Similar results were obtained for the C(3)–alkyl derivatives **1k**–**1m**, which exhibit an essentially irreversible, presumably 2e^–^ reduction process (see the Supporting Information). For the purpose of comparative analyses, the reduction potential *E*_1/2_^–1/0^ for two C(3)–amino radicals **1g** and **1h** was derived from the cathodic and anodic peak potentials (Δ*E* ≈ 80 mV).

A comparison of redox potentials in series **1** shows that replacement of the Ph substituent at the C(3) position in the parent Blatter radical **1a** with an amino group lowers the oxidation potential *E*_1/2_^0/+1^ by 0.19 V in **1i** and up to 0.27 V in **1h** (Table 2). Replacement of the Ph group in the Blatter radical **1a**, with an alkyl substituent in series **1k**–**1m**, also causes an anodic shift of the potentials, although to a lesser extent, when compared to the amines (Table 2).

**Figure 6:**
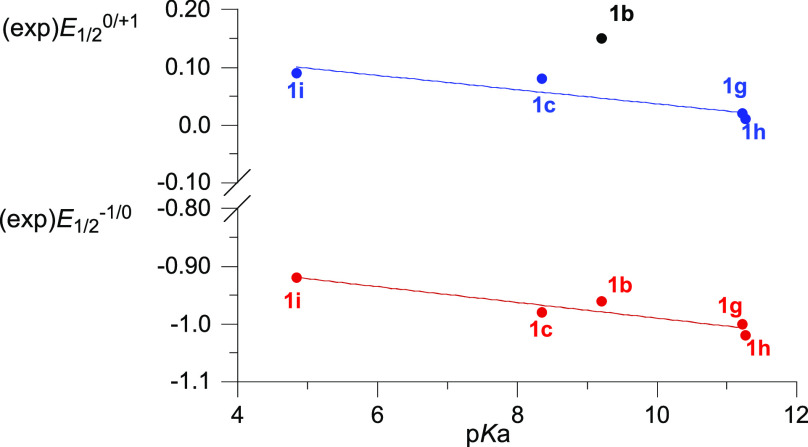
Plot of half-wave oxidation (*E*_1/2_^0/+1^) and reduction (*E*_1/2_^–1/0^) potentials in C(3)-amino series **1** vs p*K*_a_ for the corresponding amines.

For a quantitative analysis of the substituent effect on redox behavior of 3-amino-substituted derivatives **1**, the p*K*_a_ values of the corresponding amines^65^ were used, since Hammett constants are not available for many of these substituents. Thus, both oxidation and reduction potentials correlate well with the p*K*_a_ values^65^ of the corresponding amines (Figure 6); the increasing basicity of the amine corresponds to more cathodic redox potentials. The only exception from this trend is **1b** (X = NH_2_), for which, *E*_1/2_^0/+1^ is too anodic by about 0.1 V, according to the correlation.

**Figure 7:**
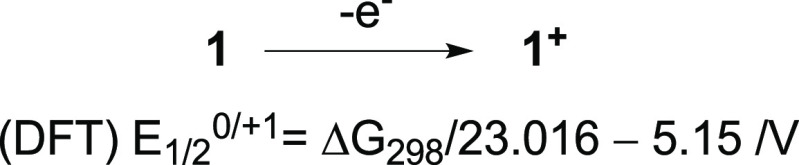
Oxidation of radicals **1** and conversion of the calculated Δ*G*_298_ in kcal mol^–1^ to the oxidation potential *E*_1/2_^0/+1^ in V vs Fc/Fc^+^.

**Figure 8:**
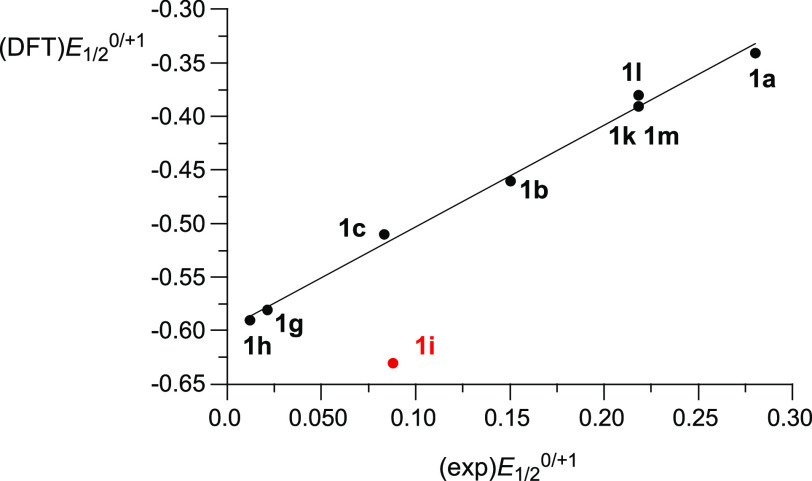
A comparison of experimental and DFT-calculated oxidation potentials *E*_1/2_ in series **1**. Best-fit line excluding the data point for **1i**: (DFT)*E*_1/2_^0/+1^ = (exp)*E*_1/2_^0/+1^ – 0.605(3), *r*^2^ = 0.992.

The oxidation process in series **1** shown in Figure 7 was modeled using the (U)B3LYP/6-31++G(2d,p)//(U)B3LYP/6-31G(2d,p) level of theory in CH_2_Cl_2_ dielectric medium. The obtained free energy of the process was expressed in volts and corrected for the absolute potential of the standard hydrogen electrode^66^ (SHE) corrected for the Fc/Fc^+^ potential versus SHE (+0.71 V at 25 °C) giving the calculated oxidation potential (DFT)*E*_1/2_^0/+1^. Calculated potentials (DFT)*E*_1/2_^0/+1^ generally correlate well with the experimental *E*_1/2_^0/+1^ values, showing that the experimental potentials are systematically underestimated by 0.605(3) V by the DFT method (Figure 8). The only exception from this trend is **1i**, for which the calculated value of oxidation potential is underestimated by about 0.1 eV presumably due to conformational aspects of the NMePh substituent not correctly accounted for by calculations.

**Figure 9:**
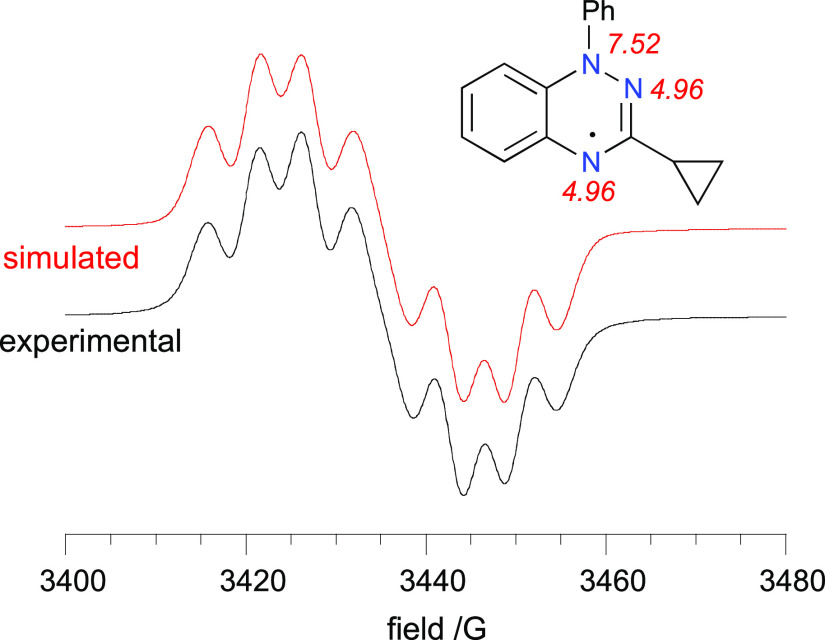
Experimental (black) and simulated (red) EPR spectra for radical **1m** recorded in benzene. (inset) An assignment of the resulting *hfcc*.

Radicals **1** exhibit typical EPR spectra consisting of seven principal lines resulting from splitting with three ^14^N nuclei (e.g., **1m** in Figure 9). The experimental hyperfine coupling constants (*hfcc*) values depend on the C(3) substituent. Thus, in comparison to the prototypical Blatter radical **1a**, introduction of an amino substituent at C(3) increases the spin density at the N(1) and N(4) atoms resulting in higher *a*_N(1)_ and *a*_N(4)_*hfcc* values. At the same time, concentration of the electron spin decreases on the N(2) atom and, consequently, diminishes the *a*_N(2)_*hfcc* values (Table 2). In the case of C(3)-alkyl derivatives **1k**–**1m** a slight decrease of *a*_N(1)_*hfcc* values and slight increase of the *a*_N(4)_*hfcc* values are observed.

**Figure 10:**
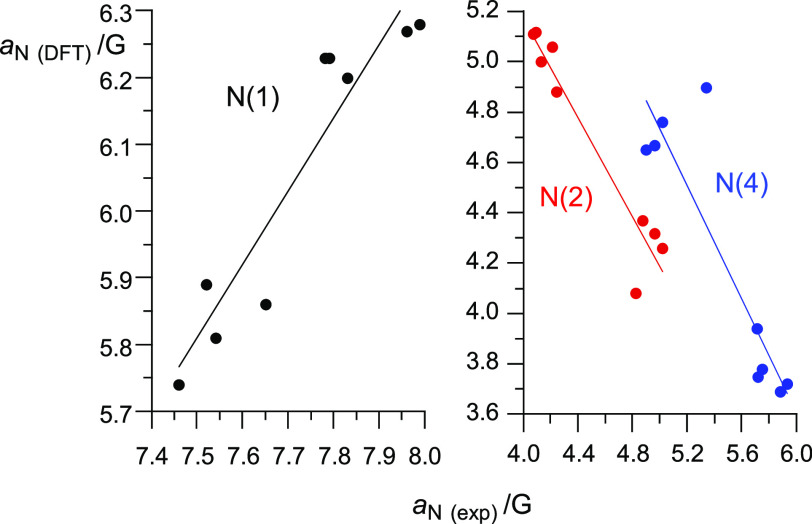
A comparison of experimental and DFT-calculated *hfcc* for the ring nitrogen atoms in series **1**. Calculated at the UCAM-B3LYP/EPR-III//UB3LYP/6-31G(2d,p) level of theory in benzene dielectric medium.

DFT calculations reproduced reasonably well the trend in the experimental *hfcc* values for series **1**. Correlations shown in Figure 10 demonstrate that the DFT method underestimates *a*_N(1)_ values by 1.7 G and *a*_N(4)_ up to 2.2 G for **1h** (X = pyrrolidyn-1-yl). The largest differences between the experimental and DFT-derived values are observed for **1l** (X = cyclohexyl), which may be related, in part, to the conformational mobility of the substituents not taken into account in calcluations.

## Conclusions

In summary, we have demonstrated that benzo[*e*][1,2,4]triazines **2** with a range of amino and alkyl substituents at the C(3) position are available by cyclization of the appropriate *N*-arylguanidines and *N*-arylamidines followed by reduction of the resulting *N*-oxides **5**. Thus, four C(3)-amino substituted *N*-oxides **5** have been prepared in good yields (up to 72%) using a one-pot process including *N*-arylation of 1-fluoro-2-nitrobenzene (**8**) with guanidines **6** followed by *t*-BuOK-promoted cyclization of the resulting *N*-arylguanidines. In contrast, synthesis of three C(3)-alkyl *N*-oxides **5** required isolation of the intermediate *N*-arylamidines **12** and their subsequent MeONa-promoted cyclization to **5** isolated in lower yields (up to 25%). The deoxygenation of **5** proceeds smoothly in all cases giving the desired benzo[*e*][1,2,4]triazines **2**, which were finally converted in good yields (up to 86%) to the corresponding C(3)-substituted radicals **1** by addition of PhLi.

In contrast to most Blatter radicals, the radicals in series **1** exhibit limited stability to chromatographic solid support. They can, however, be easily purified by passing through a short diatomaceous earth pad, washing with *n*-pentane, and recrystallization from *n*-heptane. Most radicals **1** are stable after isolation except those containing cyclohexyl (**1l**) and cyclopropyl (**1m**) substituents at the C(3) position, which are liquids and thus slowly decompose on standing.

The experimental redox potentials of 3-amino derivatives **1** correlate well with p*K*_a_ values of the corresponding amines. A good correlation was also obtained for experimental and calculated (DFT) oxidation potentials (*E*_1/2_^0/+1^) of newly synthesized radicals **1**, which offers a tool for predicting oxidation potential values for other C(3)-amino derivatives.

A spectroscopic analysis augmented with TD DFT calculations revealed that the C(3) substituent impacts on the position and origin of the lowest π–π* excitation: The electron-donating group destabilizes the β-HOMO and narrows the HOMO–LUMO gap. Consequently, the lowest-energy excitation changes its character from a nearly pure α-HOMO → α-LUMO transition for the Blatter radical **1a**, through a comparable contribution of α-HOMO → α-LUMO and β-HOMO → β-LUMO transitions for C(3)-alkyl, to purely α-HOMO → α-LUMO for C(3)–amino derivatives **1**.

In comparison to the existing methods for the preparation of 3-amino and 3-alkyl derivatives of benzo[*e*][1,2,4]triazine **2**,^33^ the presented methodology allows one to avoid multistep procedures with poorly soluble intermediates. It offers an alternative access to benzo[*e*][1,2,4]triazines **2**, which serve as convenient precursors to radicals **1** with greater control of their electrochemical and spectroscopic properties. This opens up new opportunities in structural manipulation with the C(3) substituent of benzo[*e*][1,2,4]triazin-4-yls providing a tool for the designing of radicals that show greater functional flexibility and structural variety for modern materials applications.

## Computational Details

All calculations were carried out using the Gaussian 09 suite of programs.^67^ Geometry optimizations were carried out at the UB3LYP/6-31G(2d,p) level of theory using tight convergence criteria and no symmetry constraints. Analytical second derivatives were computed using a vibrational analysis to confirm each stationary point to be a minimum by yielding zero imaginary frequencies.

Electronic excitation energies of radicals **1** in CH_2_Cl_2_ dielectric medium were obtained at the UCAM-B3LYP/6-31++G(2d,p) // UB3LYP/6-31G(2d,p) level of theory using the TD-DFT method.^68^ The solvation model was implemented with the polarizable continuum model (PCM)^69^ using the SCRF (solvent = CH_2_Cl_2_) keyword.

Isotropic Fermi contact coupling constants for radicals **1** were calculated using the UCAM-B3LYP/EPR-III // UB3LYP/6-31G(2d,p) method in benzene dielectric medium requested with the SCRF (solvent = benzene) keyword (PCM model).^69^ Other computational details are provided in the Supporting Information.

## Experimental Section

### General

Commercially reagents and solvents were used as obtained. Reactions were carried out under inert atmosphere (N_2_ or Ar gas), and subsequent reaction workups were conducted in air. Heat for the reactions requiring elevated temperatures was supplied using oil baths. Volatiles were removed under reduced pressure. Reaction mixtures and column eluents were monitored by TLC using aluminum-backed thin layer chromatography plates (Merck Kieselgel 60 F_254_). The plates were observed under UV light at 254 and 365 nm. Melting points were determined on a Melt-Temp II Apparatus in capillaries, and they are uncorrected. ^1^H and ^13^C{^1^H} NMR spectra were obtained at 400 and 100 MHz, respectively, on a Bruker Avance NMR spectrometer in CDCl_3_ and referenced to the solvent (δ = 7.26 ppm for ^1^H and δ = 77.16 ppm for ^13^C{^1^H})^70^ or in DMSO-*d*_6_ and referenced to the solvent (δ = 2.50 ppm for ^1^H and δ = 39.52 ppm for ^13^C{^1^H}),^70^ unless otherwise specified. UV–Vis spectra were recorded on a Jasco V770 spectrophotometer in spectroscopic-grade CH_2_Cl_2_ at concentrations in the range of (1.5–10) × 10^–5^ M. IR spectra were recorded using a Nexus FT-IR Thermo Nilolet IR spectrometer in KBr pellets. High-resolution mass spectrometry (HRMS) measurements were performed using SYNAPT G2-Si High-Definition Mass Spectrometry equipped with an electrospray ionization (ESI) mass analyzer.

### Preparation of Radicals 1. General Procedure^23,24^

A 1.75 M solution of PhLi (1.3 mmol, 1.3 equiv) in *n*-dibutyl ether was added dropwise to a stirred solution of the 3-substituted benzo[*e*][1,2,4]triazine **2** (1 mmol, 1 equiv) in dry THF (8 mL, 0.13 M) at −78 °C under Ar atmosphere, and the resulting mixture was stirred for 40 min at −78 °C and then for 1 h at rt. The reaction flask was opened, and the stirring was continued overnight in air at rt. After evaporation of the solvent, the residue was dissolved in CH_2_Cl_2_ and passed through a short diatomaceous earth pad, and the solvent was evaporated. The obtained solid was treated with *n-*pentane, the solution was filtered, and the solvent was evaporated giving crude radical **1**, which was recrystallized from *n*-heptane.

#### 3-(Piperidin-1-yl)-1-phenyl-1,4-dihydrobenzo[e][1,2,4]triazin-4-yl (1g)

Following the general procedure, radical **1g** (116.7 mg, 86% yield) was obtained as a dark green solid starting from 99.6 mg (0.465 mmol) of 3-(piperidin-1-yl)benzo[*e*][1,2,4]triazine (**2g**). mp 115–117 °C (*n*-heptane). IR ν 2932, 2850, 1512, 1481, 1445, 1333, 1281, 1245, 1207, 1117, 1027, 953, 778, 739, 699, 608 cm^–1^. UV–Vis (CH_2_Cl_2_) λ_max_ (log ε) 265 (4.44), 326 (3.83), 413 (3.50), 593 (3.17) nm. ESI(+)-MS, *m*/*z* 291 (38, [M]^+^), 293 (100, [M + 2H]^+^). HRMS (ESI+-TOF) *m*/*z* [M]^+^ calcd for C_18_H_19_N_4_ 291.1610, found 291.1606. Anal. Calcd for C_18_H_19_N_4_: C, 74.20; H, 6.57; N, 19.23. Found: C, 74.23; H, 6.59; N, 19.11%.

#### 3-(Pyrrolidin-1-yl)-1-phenyl-1,4-dihydrobenzo[e][1,2,4]triazin-4-yl (1h)

Following the general procedure, radical **1h** (46.0 mg, 72% yield) was obtained as a dark green solid starting from 46.2 mg (0.230 mmol) of benzo[*e*][1,2,4]triazine **2h**. mp 134–135 °C (*n*-heptane). IR ν 3052, 2963, 2923, 2860, 1517, 1477, 1448, 1329, 1261, 1022, 750, 698 cm^–1^. UV–Vis (CH_2_Cl_2_) λ_max_ (log ε) 264 (4.41), 326 (3.75), 412 (3.44), 595 (3.13) nm. ESI(+)-MS, *m*/*z* 277 (70, [M]^+^), 279 (100, [M + 2H]^+^). HRMS (ESI+-TOF) *m*/*z* [M]^+^ calcd for C_17_H_17_N_4_ 277.1453, found 277.1442. Anal. Calcd for C_17_H_17_N_4_: C, 73.63; H, 6.18; N, 20.20; for C_17_H_17_N_4_·1/4H_2_O: C, 72.44; H, 6.26; N, 19.88. Found: C, 72.48; H, 6.39; N, 19.18%.

#### 3-(*N*-Methyl-*N*-phenylamino)-1-phenyl-1,4-dihydrobenzo[e][1,2,4]triazin-4-yl (1i)

Following the general procedure, radical **1i** (19.0 mg, 73% yield) was obtained as a dark green solid starting from 20.1 mg (0.085 mmol) of benzo[*e*][1,2,4]triazine **2i**. mp 142–144 °C (*n*-heptane). IR ν 2931, 2850, 1594, 1476, 1399, 1334, 1116, 1026, 754, 693 cm^–1^. UV–Vis (CH_2_Cl_2_) λ_max_ (log ε) 284 (4.41), 326 (3.85), 412 (3.50), 590 (3.19) nm. ESI(+)-MS, *m*/*z* 313 (50, [M]^+^), 315 (100, [M + 2H]^+^). HRMS (ESI+-TOF) *m*/*z* [M]^+^ calcd for C_20_H_17_N_4_ 313.1453, found 313.1456. Anal. Calcd for C_20_H_17_N_4_: C, 76.65; H, 5.47; N, 17.88. Found: C, 76.25; H, 5.56; N, 17.59%.

#### 3-(*tert*-Butyl)-1-phenyl-1,4-dihydrobenzo[e][1,2,4]triazin-4-yl (1k)

Following the general procedure, radical **1k** (71.6 mg, 76% yield) was obtained as a dark purple solid starting from 66.6 mg (0.356 mmol) of benzo[*e*][1,2,4]triazine **2k**. mp 106–108 °C (*n*-heptane). IR ν 2957, 2926, 2862, 1581, 1479, 1399, 1328, 1247, 1188, 1071, 999, 779, 748, 697, 591 cm^–1^. UV–Vis (CH_2_Cl_2_) λ_max_ (log ε) 241 (4.36), 318 (3.87), 347 (3.82), 427 (3.51), 545 (2.98) nm. ESI(+)-MS, *m*/*z* 265 (100, [M + H]^+^). HRMS (ESI+-TOF) *m*/*z* [M + H]^+^ calcd for C_17_H_19_N_3_ 265.1579, found 265.1572. Anal. Calcd for C_17_H_18_N_3_: C, 77.24; H, 6.86; N, 15.90. Found: C, 77.23; H, 6.88; N, 15.91%.

#### 3-(Cyclohexyl)-1-phenyl-1,4-dihydrobenzo[e][1,2,4]triazin-4-yl (1l)

Following the general procedure, radical **1l** (61.8 mg, 79% yield) was obtained as a dark red oil starting from 57.5 mg (0.270 mmol) of benzo[*e*][1,2,4]triazine **2l**. IR ν 2923, 2850, 1584, 1481, 1402, 1328, 1199, 742, 692, 671, 516 cm^–1^. UV–Vis (CH_2_Cl_2_) λ_max_ (log ε) 242 (4.30), 319 (3.81), 349 (3.77), 425 (3.38), 545 (2.80) nm. ESI(+)-MS, *m*/*z* 291 (100, [M + H]^+^). HRMS (ESI+-TOF) *m*/*z* [M + H]^+^ calcd for C_19_H_21_N_3_ 291.1735, found 291.1736.

#### 3-(Cyclopropyl)-1-phenyl-1,4-dihydrobenzo[e][1,2,4]triazin-4-yl (1m)

Following the general procedure, radical **1m** (46.5 mg, 84% yield) was obtained as a dark red oil starting from 38.2 mg (0.223 mmol) of benzo[*e*][1,2,4]triazine **2m**. IR ν 2922, 2850, 1582, 1482, 1429, 1336, 1200, 1025, 926, 872, 822, 745, 697, 615, 504 cm^–1^. UV–Vis (CH_2_Cl_2_) λ_max_ (log ε) 244 (4.27), 320 (3.76), 356 (3.66), 426 (3.30), 548 (2.81) nm. ESI(+)-MS, *m*/*z* 249 (100, [M + H]^+^). HRMS (ESI+-TOF) *m*/*z* [M]^+^ calcd for C_16_H_14_N_3_ 248.1188, found 248.1189.

### Preparation of Benzo[*e*][1,2,4]triazines 2. General Procedure

A mixture of the appropriate 3-substituted benzo[*e*][1,2,4]triazine-1-oxide **5** (1 mmol, 1 equiv) and 10% Pd/C (10 mol %) in EtOH/AcOEt (1:1, 6 mL) was stirred at rt under H_2_ atmosphere (balloon) until the TLC analysis showed the absence of the starting material. The mixture was filtered through a short diatomaceous earth (Cellite) pad, and the solvent was evaporated giving benzo[*e*][1,2,4]triazine **2** as a yellow solid.

#### 3-(Morpholin-4-yl)benzo[e][1,2,4]triazine (2c)^33^

Following the general procedure, benzo[*e*][1,2,4]triazine **2c** (46.0 mg, 99% yield) was obtained as a yellow solid starting from 49.8 mg (0.210 mmol) of *N*-oxide **5c**. Analytical data was identical to that reported previously.^33^

#### 3-(Piperidin-1-yl)benzo[e][1,2,4]triazine (2g)

Following the general procedure, benzo[*e*][1,2,4]triazine **2g** (46.1 mg, 99% yield) was obtained from 50.0 mg (0.220 mmol) of *N*-oxide **5g** as a yellow oil. ^1^H NMR (CDCl_3_, 400 MHz) δ 8.18 (d, *J* = 8.2 Hz, 1H), 7.66 (ddd, *J*_1_ = 8.3 Hz, *J*_2_ = 7.0 Hz, *J*_3_ = 1.3 Hz, 1H), 7.54 (d, *J* = 8.5 Hz, 1H), 7.34 (ddd, *J*_1_ = 8.1 Hz, *J*_2_ = 7.0 Hz, *J*_3_ = 1.1 Hz, 1H), 4.05 (t, *J* = 3.3 Hz, 4H), 1.75–1.69 (m, 6H). ^13^C{^1^H} NMR (CDCl_3_, 100 MHz) δ 158.7, 142.6, 142.2, 135.4, 129.8, 126.5, 124.6, 45.0, 25.9, 24.9. ESI(+)-MS, *m*/*z* 215 (100, [M + H]^+^). HRMS (ESI+-TOF) *m*/*z* [M + H]^+^ calcd for C_12_H_15_N_4_ 215.1297, found 215.1299.

#### 3-(Pyrrolidin-1-yl)benzo[e][1,2,4]triazine (2h)

Following the general procedure, benzo[*e*][1,2,4]triazine **2h** (106 mg, 95%) was obtained from 120 mg (0.556 mmol) of *N*-oxide **5h**. Recrystallization from *n*-heptane gave analytically pure product. mp 83–84 °C (*n*-heptane). ^1^H NMR (CDCl_3_, 400 MHz) δ 8.22 (dd, *J*_1_ = 8.4 Hz, *J*_2_ = 0.9 Hz, 1H), 7.68 (ddd, *J*_1_ = 8.3 Hz, *J*_2_ = 6.7 Hz, *J*_3_ = 1.4 Hz, 1H), 7.60 (d, *J* = 8.1 Hz, 1H), 7.36 (ddd, *J*_1_ = 8.2 Hz, *J*_2_ = 6.7 Hz, *J*_3_ = 1.4 Hz, 1H), 3.84 (bs, 4H), 2.10 (t, *J* = 8.1 Hz, 4H). ^13^C{^1^H} NMR (CDCl_3_, 100 MHz) δ 157.4, 142.9, 142.4, 135.4, 130.0, 126.5, 124.4, 47.0, 25.6. ESI(+)-MS, *m*/*z* 201 (100, [M + H]^+^). HRMS (ESI+-TOF) *m*/*z* [M + H]^+^ calcd for C_11_H_13_N_4_ 201.1140, found 201.1136. Anal. Calcd for C_11_H_12_N_4_: C, 65.98; H, 6.04; N, 27.98. Found: C, 65.71; H, 5.93; N, 27.84%.

#### 3-(*N*-Methyl-*N*-phenylamino)benzo[e][1,2,4]triazine (2i)

Following the general procedure, benzo[*e*][1,2,4]triazine **2i** (98.0 mg, 99% yield) was obtained from 106.0 mg (0.421 mmol) of *N*-oxide **5i** as a yellow solid. Recrystallization from *n*-heptane gave analytically pure product. mp 98–99 °C (*n*-heptane). ^1^H NMR (CDCl_3_, 400 MHz) δ 8.25 (dd, *J*_1_ = 8.4 Hz, *J*_2_ = 0.9 Hz, 1H), 7.73 (ddd, *J*_1_ = 8.3 Hz, *J*_2_ = 6.7 Hz, *J*_3_ = 1.4 Hz, 1H), 7.64 (d, *J* = 8.1 Hz, 1H), 7.48–7.41 (m, 5H), 7.32–7.30 (m, 1H), 3.74 (s, 3H). ^13^C{^1^H} NMR (CDCl_3_, 100 MHz) δ 159.0, 144.8, 143.0, 142.1, 135.6, 129.9, 129.5, 127.0, 126.5, 126.4, 125.5, 39.1. ESI(+)-MS, *m*/*z* 237 (100, [M + H]^+^). HRMS (ESI+-TOF) *m*/*z* [M + H]^+^ calcd for C_14_H_13_N_4_ 237.1140, found 237.1134. Anal. Calcd for C_14_H_12_N_4_: C, 71.17; H, 5.12; N, 23.71. Found: C, 70.84; H, 4.95; N, 23.40%.

#### 3-(*tert*-Butyl)benzo[e][1,2,4]triazine (2k)^71^

Following the general procedure, benzo[*e*][1,2,4]triazine **2k** (84.2 mg, 99% yield) was obtained from 92.3 mg (0.454 mmol) of *N*-oxide **5k** as a yellow solid. The analytical data was identical to that reported previously.^67^

#### 3-(Cyclohexyl)benzo[e][1,2,4]triazine (2l)

Following the general procedure, benzo[*e*][1,2,4]triazine **2l** (95.5 mg, 99% yield) was obtained from 103.07 mg (0.452 mmol) of *N*-oxide **5l** as a yellow solid. Recrystallization from *n*-heptane gave analytically pure product. mp 62–63 °C (*n*-heptane). ^1^H NMR (CDCl_3_, 400 MHz) δ 8.49 (dd, *J*_1_ = 8.5 Hz, *J*_2_ = 0.5 Hz, 1H), 8.00 (d, *J* = 8.3 Hz, 1H), 7.93 (ddd, *J*_1_ = 8.8 Hz, *J*_2_ = 7.1 Hz, *J*_3_ = 1.3 Hz, 1H), 7.80 (ddd, *J*_1_ = 8.2 Hz, *J*_2_ = 7.0 Hz, *J*_3_ = 1.2 Hz, 1H), 3.40 (tt, *J*_1_ = 11.7 Hz, *J*_2_ = 3.4 Hz, 1H), 2.17 (d, *J* = 14.1 Hz, 2H), 1.98–1.76 (m, 5H), 1.52 (qt, *J*_1_ = 12.5 Hz, *J*_3_ = 3.1 Hz, 2H), 1.40 (tt, *J*_1_ = 12.0 Hz, *J*_2_ = 3.3 Hz, 1H). ^13^C{^1^H} NMR (CDCl_3_, 100 MHz) δ 169.7, 146.5, 141.1, 135.3, 129.9, 129.7, 128.8, 46.2, 32.1, 26.4, 26.0. ESI(+)-MS, *m*/*z* 214 (100, [M + H]^+^). HRMS (ESI+-TOF) *m*/*z* [M + H]^+^ calcd for C_13_H_16_N_3_ 214.1344, found 214.1350. Anal. Calcd for C_13_H_15_N_3_: C, 73.21; H, 7.09; N, 19.70. Found: C, 73.22; H, 7.13; N, 19.71%.

#### 3-(Cyclopropyl)benzo[e][1,2,4]triazine (2m)

Following the general procedure, benzo[*e*][1,2,4]triazine **2m** (90.6 mg, 99% yield) was obtained from 100.0 mg (0.535 mmol) of *N*-oxide **5m** as a yellow solid. Recrystallization from *n*-heptane gave analytically pure product. mp 62–64 °C (*n*-heptane). ^1^H NMR (CDCl_3_, 400 MHz) δ 8.43 (d, *J* = 8.5 Hz, 1H), 7.89–7.88 (m, 2H), 7.75–7.71 (m, 1H), 2.70 (tt, *J*_1_ = 8.2 Hz, *J*_2_ = 4.8 Hz, 1H), 1.44–1.40 (m, 2H), 1.31–1.26 (m, 2H). ^13^C{^1^H} NMR (CDCl_3_, 100 MHz) δ 167.5, 146.5, 141.1, 135.4, 129.7, 129.3, 128.3, 17.3, 12.0. ESI(+)-MS, *m*/*z* 172 (100, [M + H]^+^). HRMS (ESI+-TOF) *m*/*z* [M + H]^+^ calcd for C_10_H_10_N_3_ 172.0875, found 172.0876. Anal. Calcd for C_10_H_9_N_3_: C, 70.16; H, 5.30; N, 24.54. Found: C, 70.14; H, 5.28; N, 24.52%.

#### 3-Phenylbenzo[e][1,2,4]triazine-1-oxide (5a)^50^

A mixture of substituted amidine hydrochloride **7a·HCl** (700.0 mg, 4.47 mmol, 1 equiv) and MeONa (4.47 mmol, 1 equiv) in dry MeOH was stirred under N_2_ conditions for 30 min at rt. The resulting precipitated inorganic salt was filtered through a syringe filter under N_2_ athomphere. After evaporation of the solvent, the free amidine was dried under vacuum and dissolved in dry MeOH (5 mL). 1-Fluoro-2-nitrobenzene (**8**, 105.1 mg, 0.078 mL, 0.745 mmol, 0.17 equiv) was added, and the resulting mixture was refluxed overnight. Additional amounts of MeONa (0.745 mmol, 0.17 equiv) were added, and the stirring was continued under reflux for 2 h. After cooling to rt, the reaction mixture was placed in a refrigerator for 2 h, and the resulting white crystalline product was collected giving 101.2 mg (61% yield) of 3-phenylbenzo[*e*][1,2,4]triazine-1-oxide (**5a**). Recrystallization from MeOH gave analytically pure product. mp 126–128 °C (MeOH; lit.^50^ mp 118–119 °C). ^1^H NMR (CDCl_3_, 400 MHz) δ 8.54–6.46 (m, 3H), 8.10–8.06 (m, 1H), 7.97–7.91 (m, 1H), 7.72–7.67 (m, 1H), 7.58–7.50 (m, 3H). ^13^C{^1^H} NMR (CDCl_3_, 100 MHz) δ 160.8, 147.9, 135.8, 134.2, 133.6, 132.1, 130.2, 129.5, 128.9, 128.6, 120.4. ESI(+)-MS, *m*/*z* 224 (100, [M + H]^+^). HRMS (ESI+-TOF) *m*/*z* [M + H]^+^ calcd for C_13_H_10_N_3_O 224.0824, found 224.0824. Anal. Calcd for C_13_H_9_N_3_O: C, 69.95; H, 4.06; N, 18.82. Found: C, 70.01; H, 3.98; N, 18.77%.

### Preparation of *N*-Oxides 5c, 5g–5i. General Procedure

A mixture of the appropriate guanidine hydrochloride **6c·HCl**, **6g·HCl–6i·HCl** (6 mmol, 6 equiv) and EtONa (6 mmol, 6 equiv) in dry EtOH (0.9 mL, 1.11 M) was stirred under N_2_ conditions for 30 min at rt. The resulting precipitated inorganic salt was filtered through a syringe filter under N_2_ atmosphere. After evaporation of the solvent, the free guanidine **6** was dried under vacuum and dissolved in dry MeCN (0.8 mL/1 mmol). 1-Fluoro-2-nitrobenzene (**8**, 1 mmol, 1 equiv) was added, and the resulting mixture was stirred overnight at 78 °C. *t*-BuOK (1.5 mmol, 1.5 equiv) was added, and the stirring was continued at 78 °C. After 1 h an additional portion of *t*-BuOK (1.5 mmol, 1.5 equiv) was added, and the reaction was continued for 3 h. The solvent was evaporated, and the residue was dissolved in AcOEt (10 mL) and washed with water (2 × 10 mL); the solvents were evaporated, and the residue was purified by column chromatography (pet. ether/AcOEt, 4:1) giving pure 3-substituted benzo[*e*][1,2,4]triazine-1-oxide **5**.

#### 3-(Morpholin-4-yl)benzo[e][1,2,4]triazine-1-oxide (5c)

Following the general procedure, *N*-oxide **5c** (49.8 mg, 72% yield) was obtained as a yellow solid starting from morpholine-4-carboxamidine hydrochloride (**6c·HCl**, 300 mg, 1.80 mmol) and 1-fluoro-2-nitrobenzene (**8**, 42.3 mg, 31.2 mL, 0.30 mmol). Recrystallization from ethanol gave analytically pure product. mp 172–174 °C (EtOH). ^1^H NMR (DMSO-*d*_6_, 400 MHz) δ 8.15 (d, *J* = 8.6 Hz, 1H), 7.82 (ddd, *J*_1_ = 8.4 Hz, *J*_2_ = 7.1 Hz, *J*_3_ = 1.4 Hz, 1H), 7.60 (d, *J* = 8.5 Hz, 1H), 7.39 (ddd, *J*_1_ = 8.4 Hz, *J*_2_ = 7.1 Hz, *J*_3_ = 1.1 Hz, 1H), 3.73–3.75 (m, 4H), 3.69–3.71 (m, 4H). ^13^C{^1^H} NMR (DMSO-*d*_6,_ 100 MHz) δ 157.9, 148.2, 136.1, 129.6, 126.3, 125.5, 119.9, 65.8, 44.1. IR ν 1549, 1430, 1346, 1233, 1114, 999, 864, 759 cm^–1^. ESI(+)-MS, *m*/*z* 233 (100, [M + H]^+^). HRMS (ESI+-TOF) *m*/*z* [M + H]^+^ calcd for C_11_H_13_N_4_O_2_ 233.1039, found 233.1038. Anal. Calcd for C_11_H_12_N_4_O_2_: C, 56.89; H, 5.21; N, 24.12. Found: C, 56.87; H, 5.19; N, 24.08%.

#### 3-(Piperidin-1-yl)benzo[e][1,2,4]triazine-1-oxide (5g)

Following the general procedure, *N*-oxide **5g** (90.8 mg, 54% yield) was obtained as a yellow solid starting from piperidine-1-carboxamidine hydrochloride (**6g·HCl**, 717 mg, 4.38 mmol) and 1-fluoro-2-nitrobenzene (**8**, 103 mg, 76.9 mL, 0.73 mmol). Recrystallization from *n*-heptane gave analytically pure product. mp 104–106 °C (*n*-heptane). ^1^H NMR (DMSO-*d*_6_, 400 MHz) δ 8.13 (dd, *J*_1_ = 8.6 Hz, *J*_2_ = 0.7 Hz, 1H), 7.79 (ddd, *J*_1_ = 8.4 Hz, *J*_2_ = 6.9 Hz, *J*_3_ = 1.3 Hz, 1H), 7.57 (d, *J* = 8.5 Hz, 1H), 7.24 (ddd, *J*_1_ = 8.3 Hz, *J*_2_ = 6.9 Hz, *J*_3_ = 1.0 Hz, 1H), 3.77 (t, *J* = 5.1 Hz, 4H), 1.66–1.65 (m, 2H), 1.59–1.58 (m, 4H). ^13^C{^1^H} NMR (DMSO-*d*_6_, 100 MHz) δ 157.7, 148.5, 136.0, 129.2, 126.2, 124.9, 120.0, 44.6, 25.2, 24.1. IR ν 1544, 1414, 1340, 1277, 1229, 1131, 992, 851, 768 cm^–1^. ESI(+)-MS, *m*/*z* 231 (100, [M + H]^+^). HRMS (ESI+-TOF) *m*/*z* [M + H]^+^ calcd for C_12_H_15_N_4_O 231.1246, found 231.1245. Anal. Calcd for C_12_H_14_N_4_O: C, 62.59; H, 6.13; N, 24.33. Found: C, 62.65; H, 6.09; N, 24.28%.

#### 3-(Pyrrolidin-1-yl)benzo[e][1,2,4]triazine-1-oxide (5h)

Derivative **5h** was obtained following the general procedure without the use of *t*-BuOK. Thus, using pyrrolidine-1-carboxamidine hydrochloride (**6h·HCl**, 1.32 g, 8.82 mmol) and 1-fluoro-2-nitrobenzene (**8**, 133 mg, 0.99 mL, 0.942 mmol), 3-(pyrrolidin-1-yl)benzo[*e*][1,2,4]triazine-1-oxide (**5h**) was isolated in 70% yield (142 mg) by column chromatography (pet. ether/AcOEt, 3:1). Recrystallization from EtOH gave analytically pure product. mp 180–182 °C (EtOH). ^1^H NMR (DMSO-*d*_6_, 400 MHz) δ 8.15 (dd, *J*_1_ = 8.6 Hz, *J*_2_ = 1.4 Hz, 1H), 7.88 (ddd, *J*_1_ = 8.4 Hz, *J*_2_ = 6.9 Hz, *J*_3_ = 1.5 Hz, 1H), 7.60 (dd, *J*_1_ = 8.5 Hz, *J*_2_ = 0.7 Hz, 1H), 7.33 (ddd, *J*_1_ = 8.4 Hz, *J*_2_ = 6.9 Hz, *J*_3_ = 1.3 Hz, 1H), 3.55 (bs, 4H), 1.96–1.98 (m, 4H). ^13^C{^1^H} NMR (DMSO-*d*_6_, 100 MHz) δ 156.7, 148.7, 135.8, 129.3, 126.1, 124.6, 120.0, 46.6, 24.8. ESI(+)-MS, *m*/*z* 217 (100, [M + H]^+^). HRMS (ESI+-TOF) *m*/*z* [M + H]^+^ calcd for C_11_H_13_N_4_O 217.1089, found 217.1085. Anal. Calcd for C_11_H_12_N_4_O: C, 61.10; H, 5.59; N, 25.91. Found: C, 61.12; H, 5.63; N, 26.03%.

#### 3-(*N*-Methyl-*N*-phenylamino)benzo[e][1,2,4]triazine-1-oxide (5i)

Following the general procedure *N*-oxide **5i** (110 mg, 63% yield) was obtained from *N*-methyl-*N*-phenyl-carboxamidine hydrochloride (**6i·HCl**, 1.01 g, 5.44 mmol) and 1-fluoro-2-nitrobenzene (**8**, 98.0 mg, 73.0 mL, 0.692 mmol). Product **5i** was isolated as the first fraction in column chromatography, which was followed by uncyclized intermediate **12i** (fraction 2). Recrystallization from ethanol gave analytically pure **5i** as a yellow solid. mp 120–122 °C (EtOH). ^1^H NMR (DMSO-*d*_6,_ 400 MHz) δ 8.16 (dd, *J*_1_ = 8.6 Hz, *J*_2_ = 0.8 Hz, 1H), 7.84 (ddd, *J*_1_ = 8.4 Hz, *J*_2_ = 6.8 Hz, *J*_3_ = 1.3 Hz, 1H), 7.65 (d, *J* = 8.5 Hz, 1H), 7.48–7.40 (m, 5H), 7.32 (t, *J* = 7.0 Hz, 1H), 3.52 (s, 3H). ^13^C{^1^H} NMR (DMSO-*d*_6_, 100 MHz) δ 158.2, 148.1, 144.1, 136.1, 130.0, 129.3, 126.7, 126.6, 126.4, 125.4, 125.8, 120.0, 38.8. IR ν 1534, 1421, 1361, 1173, 1104, 757, 694 cm^–1^. ESI(+)-MS, *m*/*z* 253 (100, [M + H]^+^). HRMS (ESI+-TOF) *m*/*z* [M + H]^+^ calcd for C_14_H_13_N_4_O 253.1089, found 253.1089. Anal. Calcd for C_14_H_12_N_4_O: C, 66.65; H, 4.79; N, 22.21. Found: C, 66.38; H, 4.72; N, 22.19%.

### Preparation of *N*-Oxides 5k–5m. General Procedure

To a solution of the appropriate *N*-(2-nitrophenyl)-alkylcarboxamidine **12** (1 mmol, 1 equiv) dissolved in MeOH (0.8 mL, 1.25 M) was added MeONa (1.5 mmol, 1.5 equiv), and the resulting reaction mixture was refluxed overnight. The solvent was evaporated, and the residue was purified by column chromatography (hexane/AcOEt, 4:1) giving pure 3-alkyl-substituted benzo[*e*][1,2,4]triazine-1-oxide **5**.

#### 3-(*tert*-Butyl)benzo[e][1,2,4]triazine-1-oxide (5k)

Following the general procedure, *N*-oxide **5k** (129.9 mg, 25% yield) was obtained as a pale yellow solid starting from *N-*(2-nitrophenyl)amidine **12k** (560.4 mg, 2.533 mmol). Recrystallization from *n*-heptane gave analytically pure product. mp 85–86 °C (*n*-heptane). ^1^H NMR (CDCl_3_, 400 MHz) δ 8.45 (d, *J* = 8.6 Hz, 1H), 8.00 (d, *J* = 8.4 Hz, 1H), 7.90 (td, *J*_1_ = 7.4 Hz, *J*_2_ = 1.0 Hz, 1H), 7.67 (td, *J*_1_ = 7.4 Hz, *J*_2_ = 1.0 Hz, 1H), 1.49 (s, 9H). ^13^C{^1^H} NMR (CDCl_3_, 100 MHz) δ 173.2, 147.5, 135.3, 132.9, 130.0, 129.2, 120.2, 39.1, 29.3. ESI(+)-MS, *m*/*z* 204 (100, [M + H]^+^). HRMS (ESI+-TOF) *m*/*z* [M + H]^+^ calcd for C_11_H_14_N_3_O 204.1137, found 204.1135. Anal. Calcd for C_11_H_13_N_3_O: C, 65.01; H, 6.45; N, 20.68. Found: C, 65.03; H, 6.47; N, 20.71%.

#### 3-(Cyclohexyl)benzo[e][1,2,4]triazine-1-oxide (5l)

Following the general procedure, *N*-oxide **5l** (44.9 mg, 22% yield) was obtained as a pale yellow solid starting from *N-*(2-nitrophenyl)amidine **12l** (218.6 mg, 0.884 mmol). Recrystallization from *n*-heptane gave analytically pure product. mp 68–70 °C (*n*-heptane). ^1^H NMR (CDCl_3_, 400 MHz) δ 8.44 (d, *J* = 8.7 Hz, 1H), 7.97 (d, *J* = 8.4 Hz, 1H), 7.90 (td, *J*_1_ = 7.8 Hz, *J*_2_ = 1.2 Hz, 1H), 7.67 (td, *J*_1_ = 7.8 Hz, *J*_2_ = 1.2 Hz, 1H), 2.96 (tt, *J*_1_*=* 8.4 Hz, *J*_2_ = 3.5 Hz, 1H), 2.08 (d, *J* = 11.8 Hz, 1H), 1.91–1.87 (m, 2H), 1.79–1.72 (m, 3H), 1.47–1.29 (m, 3H). ^13^C{^1^H} NMR (CDCl_3_, 100 MHz) δ 170.6, 147.8, 135.5, 133.5, 129.9, 129.0, 120.2, 46.0, 31.5, 26.2, 25.9. ESI(+)-MS, *m*/*z* 230 (100, [M + H]^+^). HRMS (ESI+-TOF) *m*/*z* [M + H]^+^ calcd for C_13_H_16_N_3_O 230.1293, found 230.1299. Anal. Calcd for C_13_H_15_N_3_O: C, 68.10; H, 6.59; N, 18.33. Found: C, 68.11; H, 6.64; N, 18.32%.

#### 3-(Cyclopropyl)benzo[e][1,2,4]triazine-1-oxide (5m)^72^

Following the general procedure, *N*-oxide **5m** (12.1 mg, 17% yield) was obtained as a pale yellow solid starting from *N-*(2-nitrophenyl)amidine **12m** (53.1 mg, 0.259 mmol). Recrystallization from *n*-heptane gave analytically pure product. mp 119–120 °C (*n*-heptane). ^1^H NMR (CDCl_3_, 400 MHz) δ 8.41 (d, *J* = 8.5 Hz, 1H), 7.91–7.85 (m, 2H), 7.62 (ddd, *J*_1_ = 8.5 Hz, *J*_2_ = 6.3 Hz, *J*_3_ = 2.2 Hz, 1H), 2.31 (tt, *J*_1_ = 8.2 Hz, *J*_2_ = 4.8 Hz, 1H), 1.34–1.30 (m, 2H), 1.22–1.17 (m, 2H). ^13^C{^1^H} NMR (CDCl_3_, 100 MHz) δ 168.5, 147.8, 135.6, 133.5, 129.3, 128.5, 120.3, 16.9, 11.3. ESI(+)-MS, *m*/*z* 188 (100, [M + H]^+^). HRMS (ESI+-TOF) *m*/*z* [M + H]^+^ calcd for C_10_H_10_N_3_O 188.0824, found 188.0823. Anal. Calcd for C_10_H_9_N_3_O: C, 64.16; H, 4.85; N, 22.45. Found: C, 64.43; H, 4.96; N, 22.77%.

### Attempted Preparation of 3-(Cyclopropyl)benzo[e][1,2,4]triazine-1-oxide (5m) by a Two-Step Cyclization of 12m

*N*-(2-Nitrophenyl) amidine **12m** (100 mg, 0.49 mmol) was dissolved in EtOH (3 mL), and the mixture was stirred overnight with 10% Pd/C (5.2 mg, 0.049 mmol) under H_2_ atmosphere (balloon). The mixture was filtered through a diatomaceous earth pad, which was washed with EtOH, and the filtrate was evaporated. The residue was purified by column chromatography (SiO_2_, AcOEt/MeOH, gradient up to 100% MeOH) giving benzimidazoles **15** (12.1 mg, 16% yield) and **16** (69.5 mg, 82% yield).

### Preparation of Guanidine Hydrochlorides 6·HCl. General Procedures. Method A

Following a modified literature procedure,^59^ a solution of 2-methyl-2-thiopseudourea sulfate (**9**, 1 mmol, 1 equiv) and an appropriate amine (1 mmol, 1 equiv) in water (4 mL, 0.25 M) was heated overnight under reflux. A solution of BaCl_2_ (1 mmol, 1 equiv) in water (2.5 mL, 0.4 M) was added dropwise over 30 min, and the resulting mixture was refluxed for 1 h. After cooling to rt, the resulting precipitate was filtered, and the filtrate was concentrated leaving a viscous syrup, which was dissolved in EtOH. The resulting solution was evaporated, and the residue was dried in vacuum. The obtained solid was recrystallized from a MeOH/acetone mixture (1:2) giving analytically pure salt **6·HCl**.

### Method B

Following a modified literature procedure,^60^ to a solution of appropriate amine (1 mmol, 1 equiv) in EtOH (1.5 mL, 0.67 M) was added conc. HCl (0.1 mL, 10 M) followed by a 50% aqueous solution of cyanamide (0.13 mL, 1.5 mmol, 1.5 equiv). The reaction mixture was refluxed overnight, then cooled to 0 °C followed by addition of diethyl ether. The mixture was refrigerated overnight, and the resulting solid was filtered giving the analytically pure product **6·HCl**.

### Method C

Following a modified literature procedure,^61^ to a mixture of the appropriate amine hydrochloride (1 mmol, 1 equiv) and cyanamide (1.5 mmol, 1.5 equiv) in water (1 mL, 1 M) some drops of the free amine were added until pH 8–9 was reached. The mixture was refluxed overnight. After cooling to rt the mixture was acidified with HCl to pH 4. Then water was removed in vacuum to give the guanidine salt **6·HCl**, which was recrystallized from a MeOH/acetone mixture (1:2) giving analytically pure product **6·HCl**.

#### Morpholine-4-carboxamidine Hydrochloride (6c·HCl)^59^

Following Method A, 2.32 g (90% yield) of guanidinium salt **6c·HCl** was obtained as a white solid starting from morpholine (1.74 g, 19.6 mmol) and 2-methyl-2-thiopseudourea sulfate (**9**, 2.80 g, 20.1 mmol). mp 166–168 °C (MeOH/acetone; lit.^59^ mp 138–139 °C). ^1^H NMR (DMSO-*d*_6_, 400 MHz) δ 7.74 (s, 4H), 3.62 (s, 4H), 3.44 (s, 4H). ^13^C{^1^H} NMR (DMSO-*d*_6_, 100 MHz) δ 156.7, 65.3, 45.1. ESI(+)-MS, *m*/*z* 130 (100, [M – Cl]^+^; HRMS (ESI+-TOF) *m*/*z* [M + H]^+^ calcd for C_5_H_12_N_3_O 130.0980, found 130.0983.

#### Piperidine-1-carboximidamine Hydrochloride (6g·HCl)^59^

Following Method C, 8.71 g (91% yield) of guanidinium salt **6g·HCl** was obtained as a white solid starting from 7.14 g of piperidine hydrochloride (7.14 g, 58.8 mmol) and cyanamide (3.70 g, 88.2 mmol). An analytical sample of the product could not be obtained by recrystallization, and crude product was used in the condensation reaction. ^1^H NMR (DMSO-*d*_6_, 400 MHz) δ major signals 7.52 (s, 4H), 3.38 (t, *J* = 5.5 Hz, 4H), 1.61–1.45 (m, 6H). ^13^C{^1^H} NMR (DMSO-*d*_6_, 100 MHz) δ major signals 155.8, 46.1, 25.0, 23.3. ESI(+)-MS, *m*/*z* 128 (100, [M – Cl]^+^); HRMS (ESI+-TOF) *m*/*z* [M – Cl]^+^ calcd for C_6_H_14_N_3_ 128.1188, found 128.1185.

#### Pyrrolidine-1-carboxamidine Hydrochloride (6h·HCl)

Following Method C, 1.92 g (86% yield) of guanidinium salt **6h·HCl** was obtained as a white solid starting from of pyrrolidine hydrochloride (1.61 g, 14.9 mmol) and cyanamide (0.942 g, 22.4 mmol). mp 77–79 °C (MeOH/acetone). ^1^H NMR (DMSO-*d*_6_, 400 MHz) δ 7.37 (bs, 4H), 3.31 (t, *J* = 6.2 Hz, 4H), 1.92–1.87 (m, 4H). ^13^C{^1^H} NMR (DMSO-*d*_6_, 100 MHz) δ 154.7, 47.1, 24.8. ESI(+)-MS, *m*/*z* 114 [100, [(M – HCl) + H]^+^. HRMS (ESI-TOF) *m*/*z* [M + H]^+^ calcd for C_5_H_12_N_3_ 114.1031, found 114.1035.

#### *N*-Methyl-*N*-phenylguanidine Hydrochloride (6i·HCl)

Following Method B, 2.75 g (74% yield) of guanidinium salt **6i** was obtained as a white solid starting from *N*-methylaniline (2.15 g, 20.1 mmol) and cyanamide (1.26 g, 30.0 mmol). mp 180–183 °C (Et_2_O). ^1^H NMR (DMSO-*d*_6_, 400 MHz) δ 7.52 (t, *J* = 7.7 Hz, 2H), 7.44 (t, *J* = 7.3 Hz, 1H), 7.38 (d, *J* = 7.3 Hz, 2H), 7.32 (bs, 2H) 3.27 (s, 3H). ^13^C{^1^H} NMR (DMSO-*d*_6_, 100 MHz) δ 156.9, 141.0, 130.2, 128.6, 127.1 (Me under the solvent peak). ESI(+)-MS, *m*/*z* 150 [100, [(M – HCl) + H]^+^; HRMS (ESI+-TOF) *m*/*z* [M + H]^+^ calcd for C_8_H_12_N_3_ 150.1031, found 150.1036.

#### Imidazole-1-carboxamidine Hydrochloride (6j·HCl)

Following Method C, 2.09 g (48% yield) of guanidine salt **6j** was obtained as a white solid starting from imidazole hydrochloride (3.07 g, 29.2 mmol) and cyanamide (1.85 g, 43.8 mmol). ^1^H NMR (DMSO-*d*_6_, 400 MHz) δ 14.7 (bs, 1H), 9.13 (s, 1H), 7.68 (s, 2H), 6.71 (s, 2H). ^13^C{^1^H} NMR (DMSO-*d*_6_, 100 MHz) δ 163.0, 134.1, 119.2, 118.5. ESI(+)-MS, *m*/*z* 111 [100, [(M – HCl) + H]^+^. HRMS (ESI+-TOF) *m*/*z* [(M – HCl) + H]^+^ calcd for C_4_H_7_N_4_ 111.0671, found 111.0673.

### Preparation of Amidine Hydrochlorides 7·HCl

Following a modified literature procedure,^57^ an oven-dried three-necked round-bottom flask under Ar, equipped with a stirring bar, a gas inlet, and a reflux condenser, was charged with the appropriate nitrile **10** (1 mmol) and dry EtOH (2 mL, 0.5 M). The reaction was cooled in an ice-bath, before HCl gas was bubbled through the stirred reaction mixture for 4 h. The resulting mixture was stirred at rt overnight. Subsequently, the solvent was evaporated under reduced pressure, and the resulting solid was suspended in Et_2_O and filtered; the solid was rinsed with Et_2_O and dried giving a white solid. The precipitate was then dissolved in dry EtOH under Ar, and NH_3_ gas was bubbled through the solution for 3 h. The reaction mixture was left to stir overnight at rt. Then, the solvent was evaporated under reduced pressure, and the resulting sticky solid was dried. Recrystallization from a MeOH/acetone mixture gave the desired amidine hydrochloride **7·HCl** as white crystals.

#### *tert*-Butylcarboxamidine Hydrochloride (7k·HCl)^57^

Following the general procedure, 6.66 g (48.7 mmol, 81% yield) of amidine salt **7k·HCl** was obtained from 5.00 g (6.65 mL, 60.2 mmol) of pivalonitrile (**10k**) as a white solid. ^1^H NMR (DMSO-*d*_6_, 400 MHz) δ 7.3 (bs, 4H), 1.23 (s, 9H). ^13^C{^1^H} NMR (DMSO-*d*_6_, 100 MHz) δ 177.5, 36.3, 26.9. ESI(+)-MS, *m*/*z* 101 [100, [(M–Cl]^+^]. HRMS (ESI+-TOF) *m*/*z* [M – Cl]^+^ calcd for C_5_H_13_N_2_ 101.1079, found 101.1075.

#### Cyclohexanecarboxamidine Hydrochloride (7l·HCl)^57^

Following the general procedure, 6.71 g (41.2 mmol, 90% yield) of amidine salt **7l·HCl** was obtained from 5.00 g (5.44 mL, 45.8 mmol) of cyclohexanecarbonitrile (**10l**) as a white solid. mp 217–219 °C. ^1^H NMR (DMSO-*d*_6_, 400 MHz) δ 8.87 (bs, 4H), 2.44 (t, *J* = 12.1 Hz, 1H), 1.75 (bd, *J* = 8.1 Hz, 4H), 1.65 (d, *J* = 9.6 Hz, 1H), 1.55–1.45 (m, 2H), 1.26–1.13 (m, 3H). ^13^C{^1^H} NMR (DMSO-*d*_6_, 100 MHz) δ 174.3, 41.4, 28.8, 25.1, 24.8. ESI(+)-MS, *m*/*z* 127 [100, (M – Cl)^+^]. HRMS (ESI+-TOF) *m*/*z* [M – Cl]^+^ calcd for C_7_H_15_N_2_ 127.1235, found 127.1233.

#### N′-Methyl-N-(2-Nitrophenyl)-N′-phenylguanidine (12i)

The guanidine **12i** (16 mg, 6% yield) was obtained as an unreacted intermediate in the one-pot preparation of *N*-oxide **5i** and isolated as the second fraction by column chromatography. ^1^H NMR (DMSO-*d*_6_, 400 MHz) δ 7.76 (d, *J* = 8.1 Hz, 1H), 7.44 (t, *J* = 7.8 Hz, 1H), 7.37 (t, *J* = 7.3 Hz, 2H), 7.29 (d, *J* = 7.9 Hz, 2H), 7.17 (t, *J* = 7.2 Hz, 1H), 6.99–6.94 (m, 2H), 5.49 (bs, 2H), 3.23 (s, 3H). ^13^C{^1^H} NMR (DMSO-*d*_6_, 100 MHz) δ 152.5, 145.9, 145.3, 143.0, 133.5, 129.2, 126.2, 125.9, 125.1, 124.6, 120.2. ESI(+)-MS, *m*/*z* 271 (100, [M + H]^+^). HRMS (ESI+-TOF) *m*/*z* [M + H]^+^ calcd for C_14_H_15_N_4_O_2_ 271.1195, found 271.1191.

### Preparation of *N*-Aryl Carboxamidines 12k–12m. General Procedure

A mixture of the appropriate amidine hydrochloride **7k–7m·HCl** (6 mmol, 6 equiv) and EtONa (6 mmol, 6 equiv) in dry EtOH (0.9 mL, 1.11 M) was stirred under N_2_ atmosphere for 30 min at rt. The resulting precipitated inorganic salt was filtered through a syringe filter under N_2_ atmosphere. After evaporation of the solvent, the free amidine was dried under vacuum and dissolved in dry MeCN (0.8 mL, 1.25 M). 1-Fluoro-2-nitrobenzene (**8**, 1 mmol, 1 equiv) was added, and the resulting mixture was stirred overnight at 78 °C. The solvent was evaporated, and the residue was dissolved in AcOEt (10 mL), washed with water (2 × 10 mL), and dried; the solvents were evaporated to dryness and purified by column chromatography (pet. ether/AcOEt, 2:1) giving N-substituted amidine **12**.

#### N-(2-Nitrophenyl)-*tert*-butylcarboxamidine (12k)

Following the general procedure, **12k** (558.9 mg, 88% yield) was obtained as a yellow oil starting from *tert*-butylcarboxamidine hydrochloride (**7k·HCl**, 1.72 g, 17.2 mmol) and 1-fluoro-2-nitrobenzene (**8**, 405.0 mg, 2.87 mmol). ^1^H NMR (CDCl_3_, 400 MHz) δ 7.90 (d, *J* = 8.0 Hz, 1H), 7.46 (t, *J* = 7.8 Hz, 1H), 7.04 (t, *J* = 8.1 Hz, 2H), 4.50 (bs, 2H), 1.29 (s, 9H). ^13^C{^1^H} NMR (CDCl_3_, 100 MHz) δ 165.4, 144.9, 141.8, 134.2, 125.4, 124.6, 122.4, 37.2, 28.3. ESI(+)-MS, *m*/*z* 222 (100, [M + H]^+^). HRMS (ESI+-TOF) *m*/*z* [M + H]^+^ calcd for C_11_H_16_N_3_O_2_ 222.1243, found 222.1236.

#### N-(2-Nitrophenyl)cyclohexanecarboxamidine (12l)

Following the general procedure, **12l** (98.3 mg, 75% yield) was obtained from cyclohexanecarboxamidine hydrochloride (**7l·HCl**, 500 mg, 3.10 mmol) and 1-fluoro-2-nitrobenzene (**8**, 74.8 mg, 55.8 mL, 0.53 mmol) as a yellow oil. ^1^H NMR (CDCl_3_, 400 MHz) δ 7.83 (dd, *J*_1_ = 8.3 Hz, *J*_1_ = 1.2 Hz, 1H), 7.41 (ddd, *J*_1_ = 8.4 Hz, *J*_2_ = 7.0 Hz, *J*_3_ = 1.5 Hz, 1H), 7.00 (ddd, *J*_1_ = 8.3 Hz, *J*_2_ = 7.1 Hz, *J*_3_ = 1.2 Hz, 2H), 4.65 (bs, 2H), 2.15 (tt, *J*_1_ = 11.9 Hz, *J*_2_ = 3.4 Hz, 1H), 1.92 (dd, *J*_1_ = 11.7 Hz, *J*_2_ = 3.0 Hz, 2H), 1.78**–**1.75 (m, 2H), 1.66–1.63 (m, 1H), 1.39 (qd, *J*_1_ = 9.8 Hz, *J*_2_ = 2.6 Hz, 2H), 1.30–1.17 (m, 3H). ^13^C{^1^H} NMR (CDCl_3_, 100 MHz) δ 163.3, 144.6, 141.8, 134.0, 125.2, 124.8, 122.4, 44.6, 30.6, 25.9, 25.8. ESI(+)-MS, *m*/*z* 248 (100, [M + H]^+^). HRMS (ESI+-TOF) *m*/*z* [M + H]^+^ calcd for C_13_H_18_N_3_O_2_ 248.1399, found 248.1397.

#### N-(2-Nitrophenyl)cyclopropanecarboxamidine (12m)

Following the general procedure, **12m** (716.5 mg, 94% yield) was obtained from cyclopropanecarboxamidine hydrochloride (**7m·HCl**, 2.71 g, 22.5 mmol) and 1-fluoro-2-nitrobenzene (**8**, 522 mg, 0.39 mL, 3.7 mmol) as a yellow oil. ^1^H NMR (CDCl_3_, 400 MHz) δ 7.82 (d, *J* = 8.1 Hz, 1H), 7.42 (t, *J* = 8.0 Hz, 1H), 7.02 (ddd, *J*_1_ = 8.3 Hz, *J*_2_ = 7.2 Hz, *J*_3_ = 1.1 Hz, 1H), 6.96 (bs, 1H), 4.7 (bs, 2H), 1.48–1.39 (m, 1H), 0.99 (d, *J* = 2.2 Hz, 2H), 0.82–0.79 (m, 2 H). ^13^C{^1^H} NMR (CDCl_3_, 100 MHz) δ 160.6, 144.6, 142.2, 133.9, 125.2, 125.0, 122.4, 14.6, 7.4. ESI(+)-MS, *m*/*z* 206 (100, [M + H]^+^). HRMS (ESI+-TOF) *m*/*z* [M + H]^+^ calcd for C_10_H_12_N_3_O_2_ 206.0930, found 206.0928.

#### (1-Pyrrolidin-1-yl)-2-nitrobenzene (13h)^73^

Compound **13h** (9 mg, 7% yield) was obtained as a byproduct in preparation of *N*-oxide **5h** and isolated as the first fraction by column chromatography as a pale, yellow oil. ^1^H NMR (DMSO-*d*_6_, 400 MHz) δ 7.71 (dd, *J*_1_ = 8.2 Hz, *J*_2_ = 1.6 Hz, 1H), 7.43 (ddd, *J*_1_ = 8.6 Hz, *J*_2_ = 7.0 Hz, *J*_3_ = 1.7 Hz, 1H), 7.04 (dd, *J*_1_ = 8.6 Hz, *J*_2_ = 0.9 Hz, 1H), 6.75 (ddd, *J*_1_ = 8.2 Hz, *J*_2_ = 7.1 Hz, *J*_3_ = 1.1 Hz, 1H), 3.13–3.10 (m, 4H), 1.92–1.89 (m, 4H). ^13^C{^1^H} NMR (DMSO-*d*_6_, 100 MHz) δ 142.3, 136.4, 133.3, 126.2, 116.4, 115.4, 50.1, 25.3. Affinity purification (AP)(+)-MS, *m*/*z* 193 (61, [M + H]^+^). HRMS (AP+-TOF) *m*/*z* [M + H]^+^ calcd for C_10_H_12_N_2_O_2_ 193.0977, found 193.0978.

### 1-(Imidazol-1-yl)-2-nitrobenzene (13j).^74^ Attempted Preparation of 3-(imidazol-1-yl)benzo[*e*][1,2,4]triazine-1-oxide (5j)

Reaction of imidazole-1-carboxamidine hydrochloride (**6j·HCl**, 716 mg, 4.90 mmol) using the general procedure for preparation of *N*-oxides **5** gave 1-(imidazol-1-yl)-2-nitrobenzene (**13j**) isolated by column chromatography (pet. ether/AcOEt, 1:1) in 68% yield (102 mg) as a yellow solid. mp 98–99 °C (heptane/AcOEt; lit.^74^ mp 97–98 °C). ^1^H NMR (DMSO-*d*_6_, 400 MHz) δ 8.17 (dd, *J*_1_ = 8.1 Hz, *J*_2_ = 1.2 Hz, 1H), 7.92 (s, 1H), 7.87 (ddd, *J*_1_ = 7.8 Hz, *J*_2_ = 6.4 Hz, *J*_3_ = 1.4 Hz, 1H), 7.76–7.60 (m, 2H), 7.43 (s, 1H), 7.10 (s, 1H). ^13^C{^1^H} NMR (DMSO-*d*_6_, 100 MHz) δ 144.6, 137.6, 134.5, 130.2, 129.9, 129.5, 128.9, 125.4, 120.7. ESI(+)-MS, *m*/*z* 190 (100, [M + H]^+^). HRMS (ESI+-TOF) *m*/*z* [M + H]^+^ calcd for C_9_H_8_N_3_O_2_ 190.0617, found 190.0616. Anal. Calcd for C_9_H_7_N_3_O_2_: C, 57.14; H, 3.73; N, 22.21. Found: C, 57.11; H, 3.89; N, 22.18%.

### 2-Nitroaniline (14)

*N*-(2-Nitrophenyl) amidine **12m** (57.1 mg, 0.28 mmol) was dissolved in EtOH (3 mL), and the mixture was stirred overnight with a catalytic amount of HCl (0.1 mL, 1.18 mmol) at 78 °C. The mixture was evaporated to dryness, and the residue was purified by preparative TLC (SiO_2_, hexane/AcOEt 2:1) giving aniline **14** (27.4 mg, 72% yield) as an orange solid. Analytical data was identical to that reported previously.^75^

#### 2-Cyclopropyl-1H-benzimidazole (15)^76^

This was obtained from the attempted preparation of **5m** from **12m**. Analytically pure benzimidazole **15** was obtained as colorless needles after recrystallization from CH_2_Cl_2_. mp 233–234 °C (CH_2_Cl_2_; lit.^76^ mp 227–229 °C). ^1^H NMR (DMSO-*d*_6_, 400 MHz) δ 7.44–7.37 (m, 2H), 7.12–7.05 (m, 2H), 2.10 (tt, *J*_1_ = 8.1 Hz, *J*_2_ = 5.2 Hz, 1H), 1.12–0.96 (m, 4H). ^13^C{^1^H} NMR (DMSO-*d*_6_, 100 MHz) δ 157.0, 138.5, 121.1 (2C), 114.0, 9.4, 8.77 (2C). ESI(+)-MS, *m*/*z* 159 (100, [M + H]^+^). HRMS (ESI+-TOF) *m*/*z* [M + H]^+^ calcd for C_10_H_11_N_2_ 159.0922, found 159.0925. Anal. Calcd for C_10_H_10_N_2_: C, 75.92; H, 6.37; N, 17.71. Found: C, 75.90; H, 6.35; N, 17.72%.

#### 1-Hydroxy-2-cyclopropylbenzimidazole (16)

This was obtained from the attempted preparation of **5m** from **12m**. Analytically pure benzimidazole **16** was obtained as white crystals after recrystallization from EtOH/AcOEt. mp 165–166 °C (EtOH/AcOEt). ^1^H NMR (DMSO-*d*_6_, 400 MHz) δ 11.77 (s, 1H), 7.44 (*J* = 7.9 Hz, 1H), 7.37 (*J* = 8.0 Hz, 1H), 7.16 (td, *J*_1_ = 7.7 Hz, *J*_2_ = 1.2 Hz, 1H), 7.10 (td, *J*_1_ = 7.8 Hz, *J*_2_ = 1.2 Hz, 1H), 2.28 (tt, *J* = 8.2 Hz, 4.9 Hz, 1H), 1.26–0.79 (m, 4H). ^13^C{^1^H} NMR (DMSO-*d*_6_, 100 MHz) δ 153.0, 137.7, 132.6, 121.4, 121.3, 118.3, 108.1, 8.8, 6.3. ESI(+)-MS, *m*/*z* 175 (100, [M + H]^+^). HRMS (ESI+-TOF) *m*/*z* [M + H]^+^ calcd for C_10_H_11_N_2_O 175.0871, found 175.0874. Anal. Calcd for C_10_H_10_N_2_O: C, 68.95; H, 5.79; N, 16.08. Found: C, 68.95; H, 5.68; N, 16.05%.

## Data Availability

The data underlying this study are available in the published article and its online Supporting Information. These and also raw data are available upon request from the corresponding authors.

## Supporting Information Available

NMR, IR, UV–vis, and EPR spectra, electrochemical data, and archive for DFT calculation (PDF)
FAIR data, including the primary NMR FID files, for compounds **2**, **5**, **6·HCl**, **7·HCl**, **12**, **13**, **15**, and **16** (ZIP)
