# Supplementary material for: 3-Substituted Blatter Radicals: Cyclization of N-Arylguanidines and N-Arylamidines to Benzo[e][1,2,4]triazines and PhLi Addition
Source: J Org Chem. 2023 Feb 17;88(5):2999–3011. doi: 10.1021/acs.joc.2c02703 (PMC9990070; doi:10.1021/acs.joc.2c02703)
Supplement: Supplementary file 1 — jo2c02703_si_001.pdf [file jo2c02703_si_001.pdf]

Supporting Information

For

3-Substituted Blatter radicals: Cyclization of *N*-arylguanidines and *N*-arylamidines to benzo[*e*][1,2,4]triazines and PhLi addition

Dominika Pomikło,[†] Agnieszka Bodzioch,[†] and Piotr Kaszyński[†‡§*]

[†] Centre of Molecular and Macromolecular Studies, Polish Academy of Sciences, 90-363 Łódź, Poland.

‡ Faculty of Chemistry, University of Łódź, 91-403 Łódź, Poland

[§] Department of Chemistry, Middle Tennessee State University, Murfreesboro, TN, 37132

E-mail address: piotr.kaszynski@mtsu.edu

Table of contents **Page**

1. NMR spectra ……S2
2. IR spectra ……S30
3. UV vis spectroscopy ……S32
4. Electrochemical results ……S36
5. EPR spectroscopy ……S39
6. Computational details ……S42
7. Archive for DFT calculations ……S51
8. References ……S60

1. NMR spectra

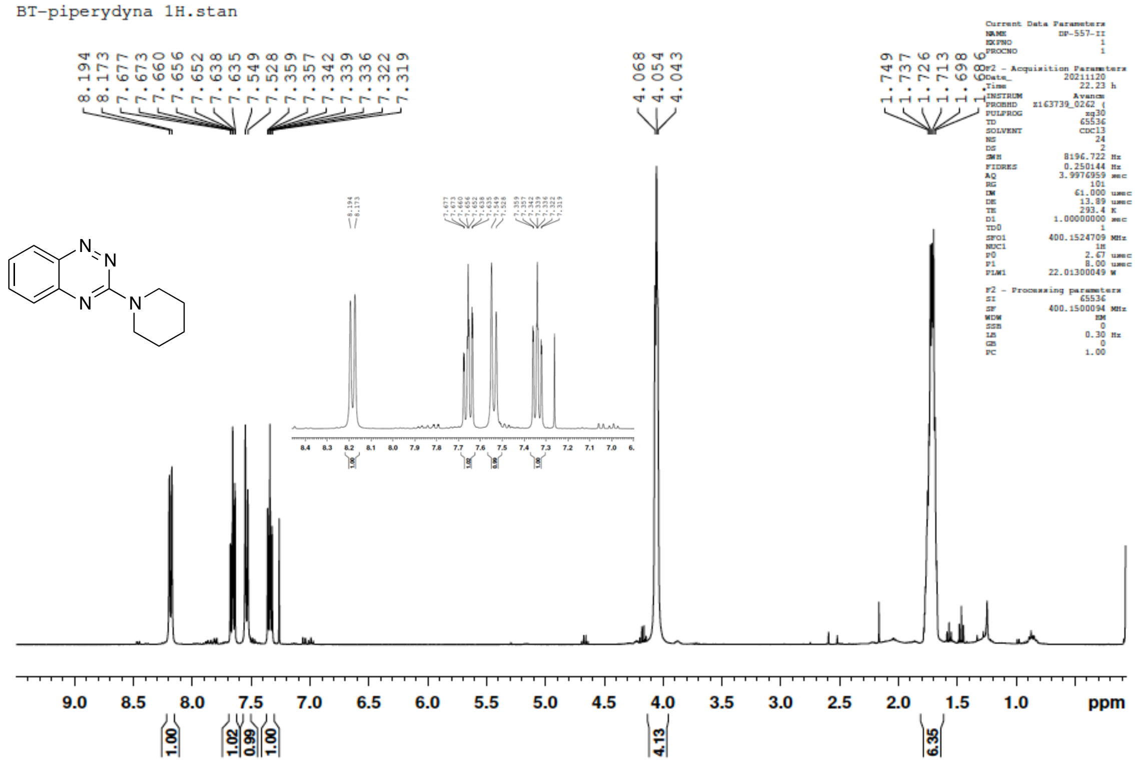

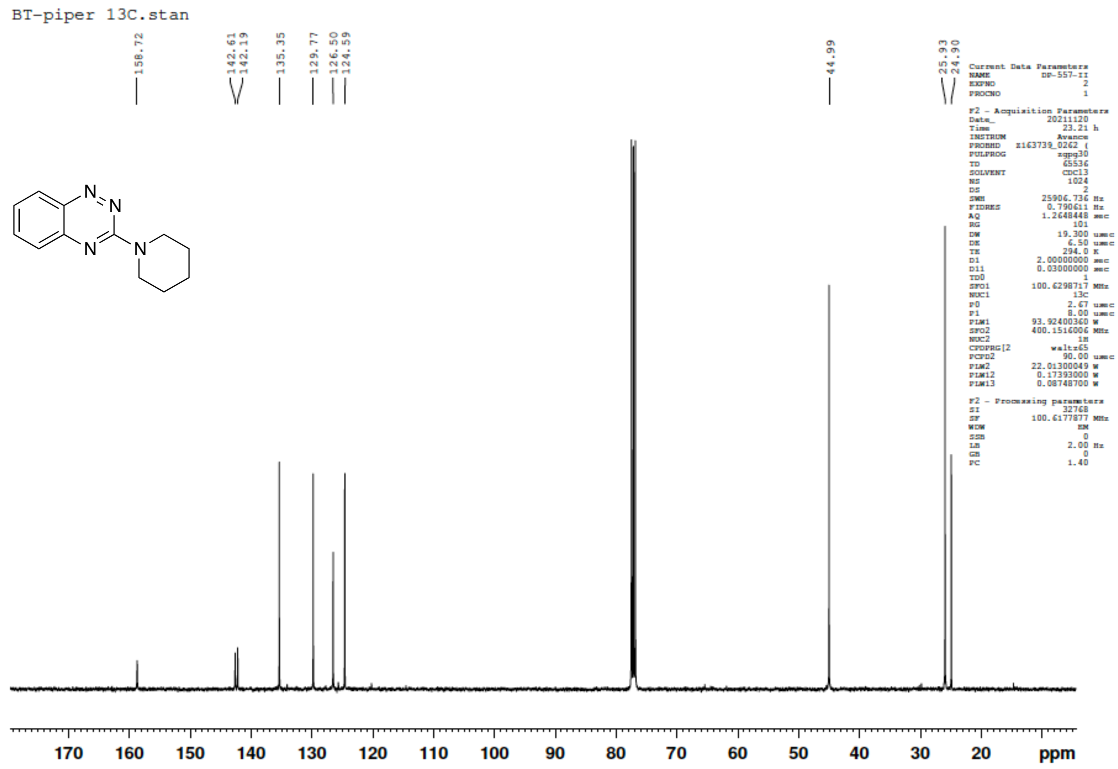

Figure S1.^{1}H NMR (400 MHz) and ^{13}C{^{1}H} NMR (100 MHz) spectra of **2g** ($CDCl_3$).

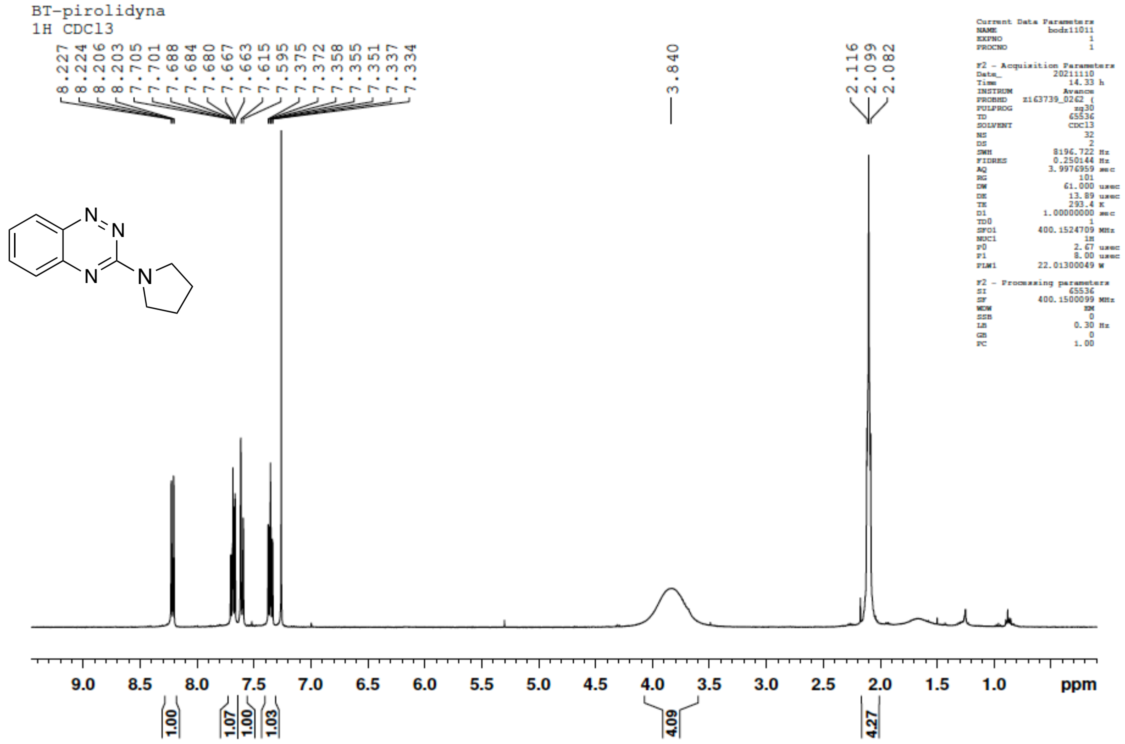

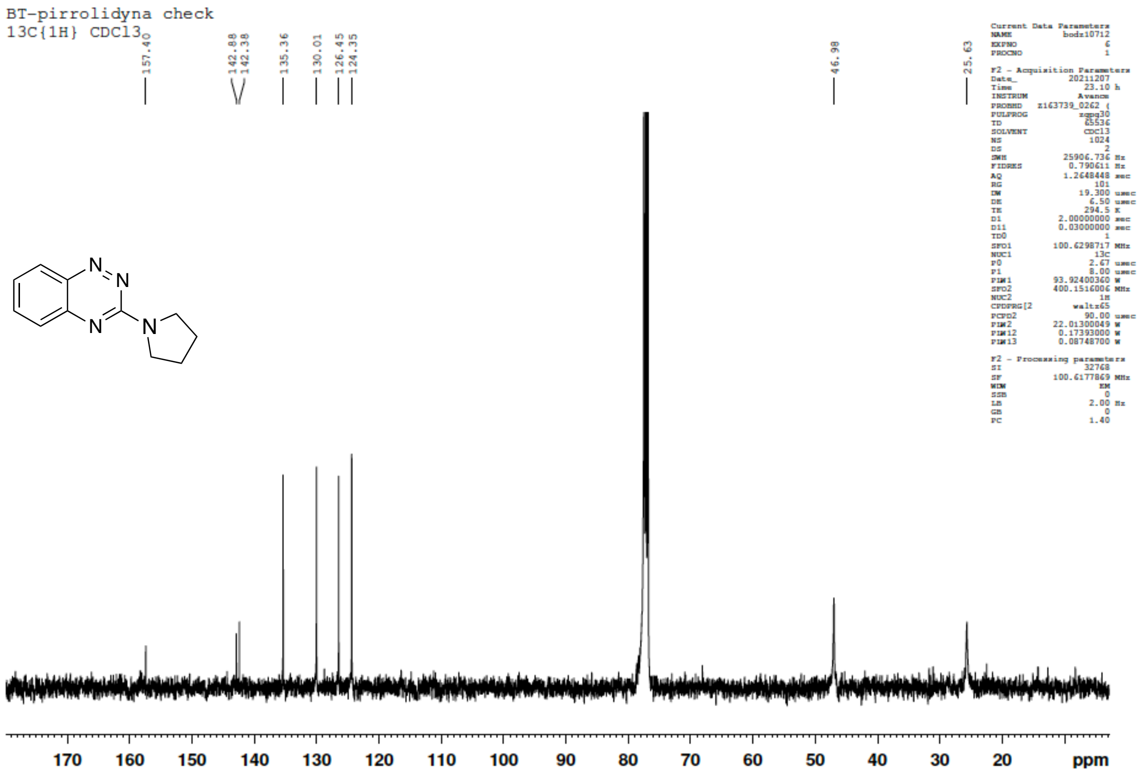

Figure S2. ^{1}H NMR (400 MHz) and $^{13}C\{^{1}H\}$ NMR (100 MHz) spectra of **2h** ($CDCl_3$).

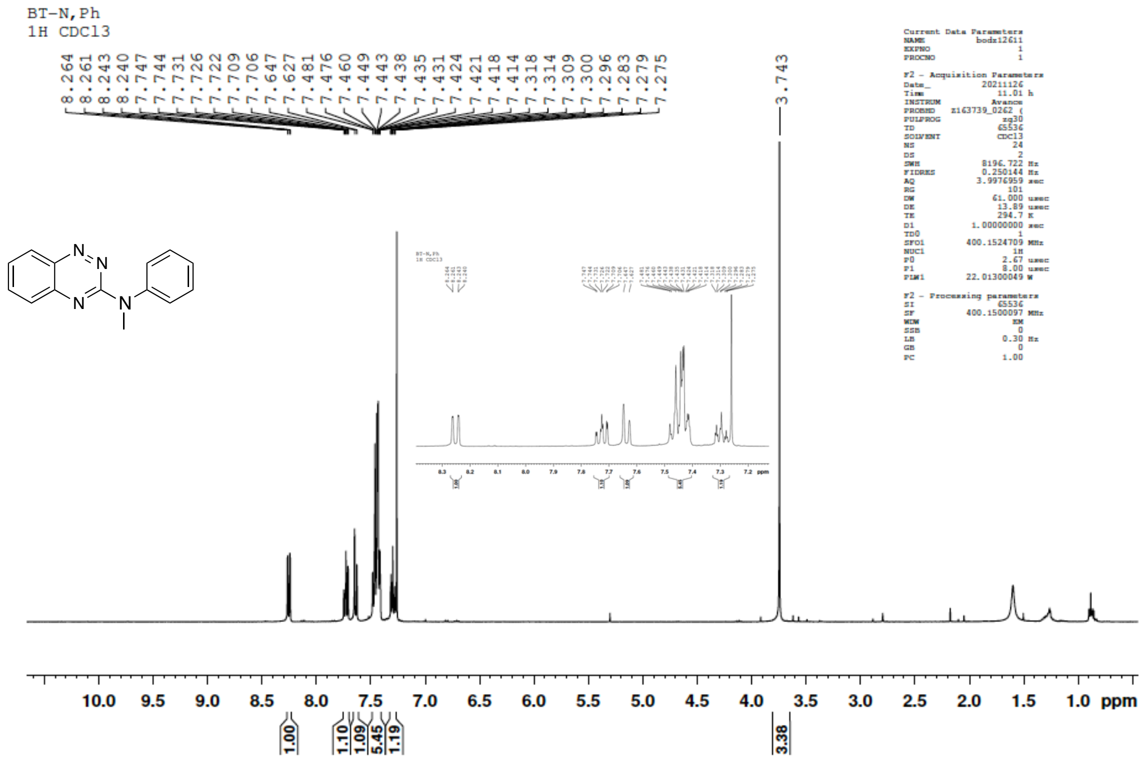

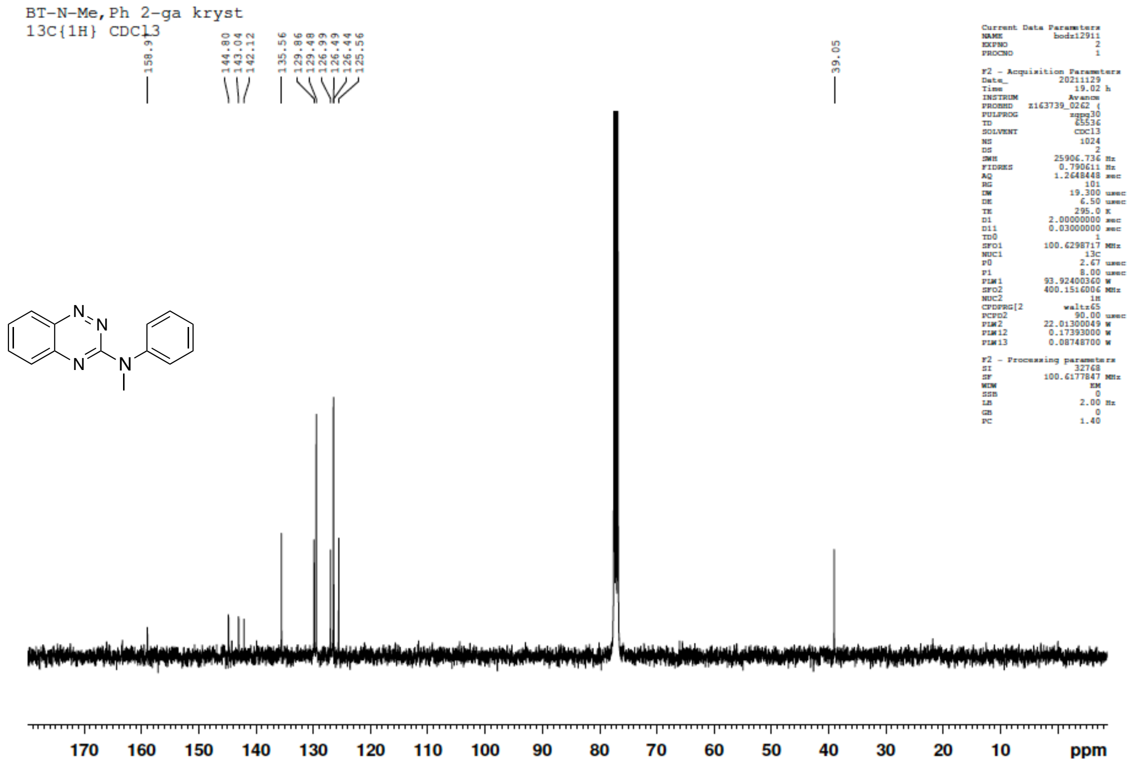

Figure S3.^{1}H NMR (400 MHz) and ^{13}C{^{1}H} NMR (100 MHz) spectra of **2i** ($CDCl_3$).

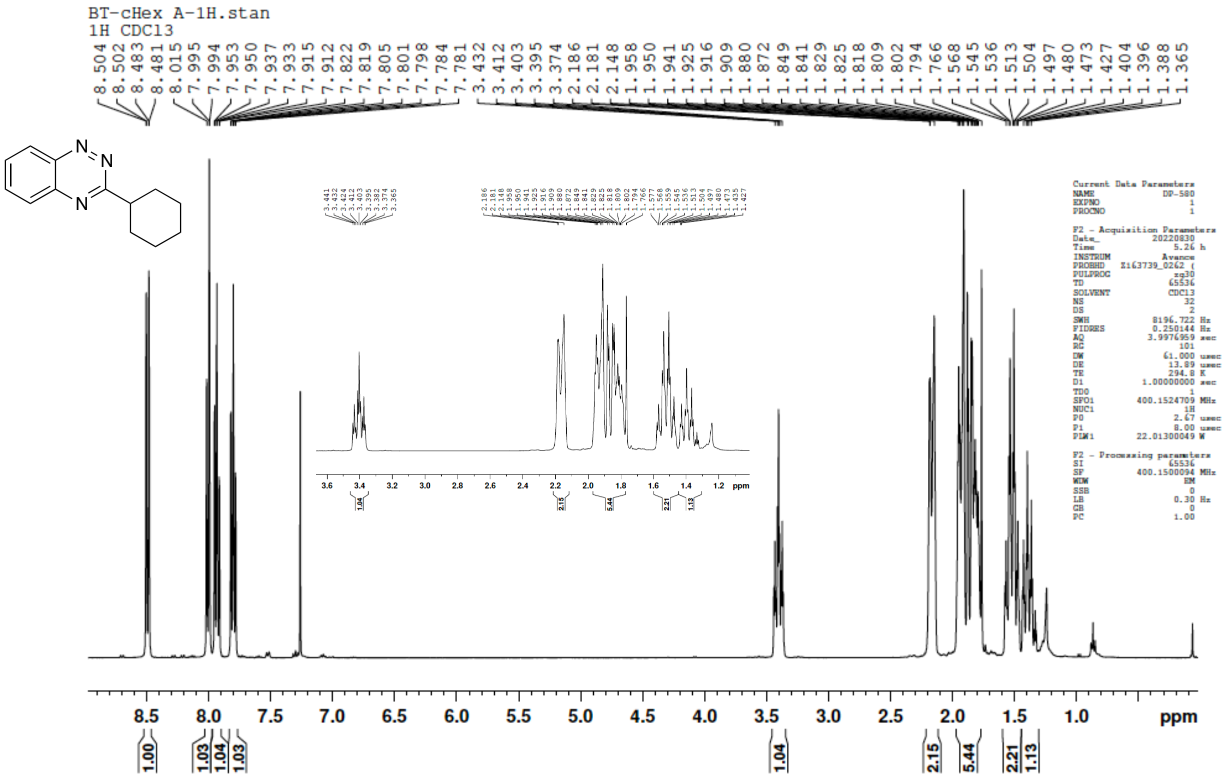

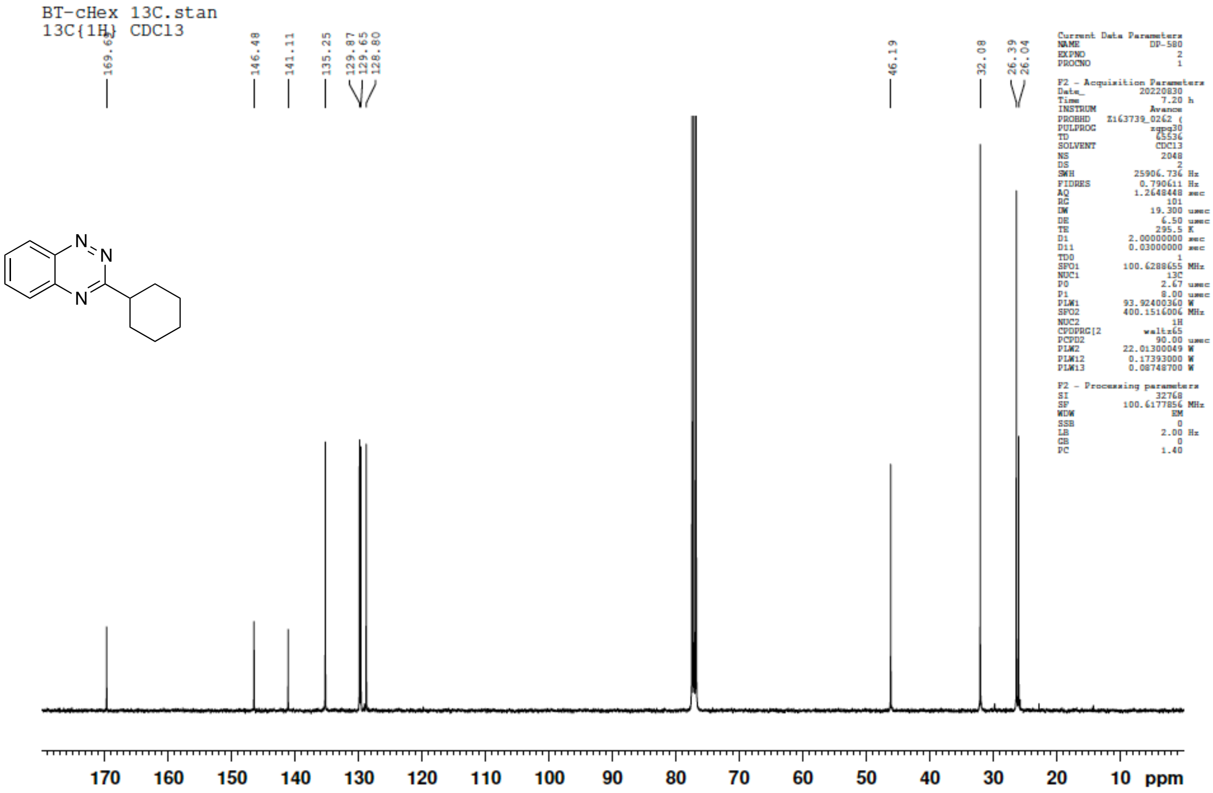

Figure S4.^{1}H NMR (400 MHz) and ^{13}C{^{1}H}NMR (100 MHz) spectra of **2l** ($CDCl_3$).

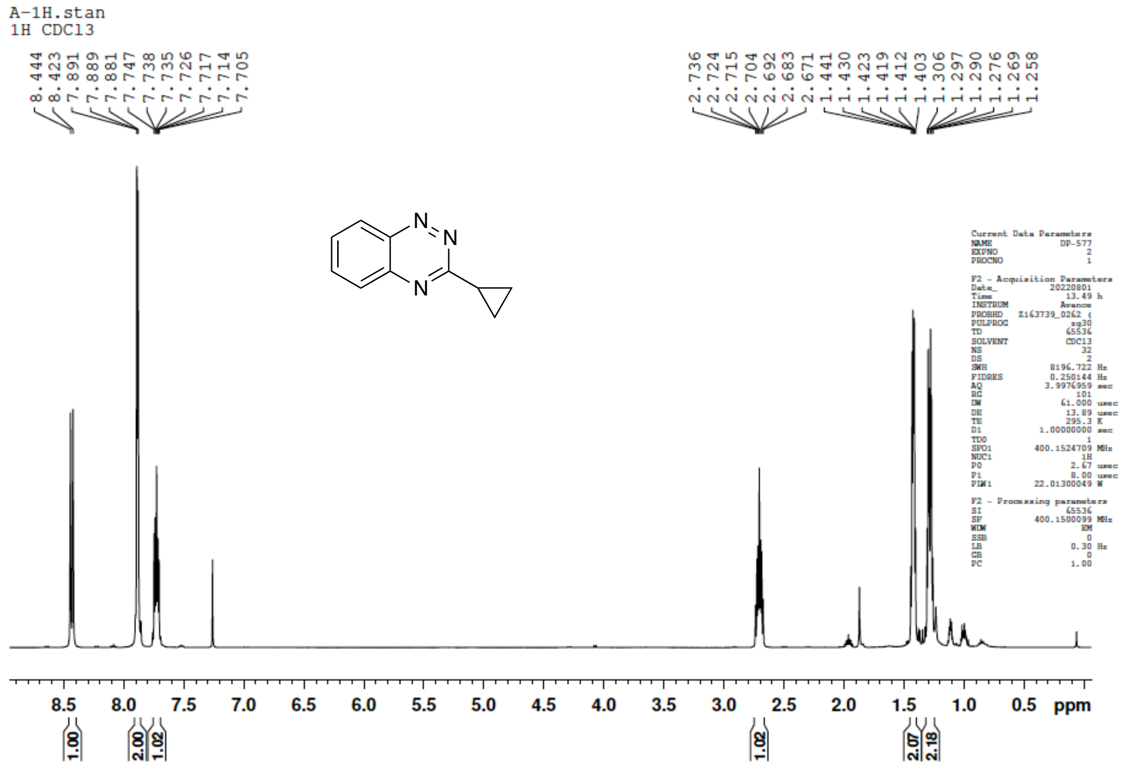

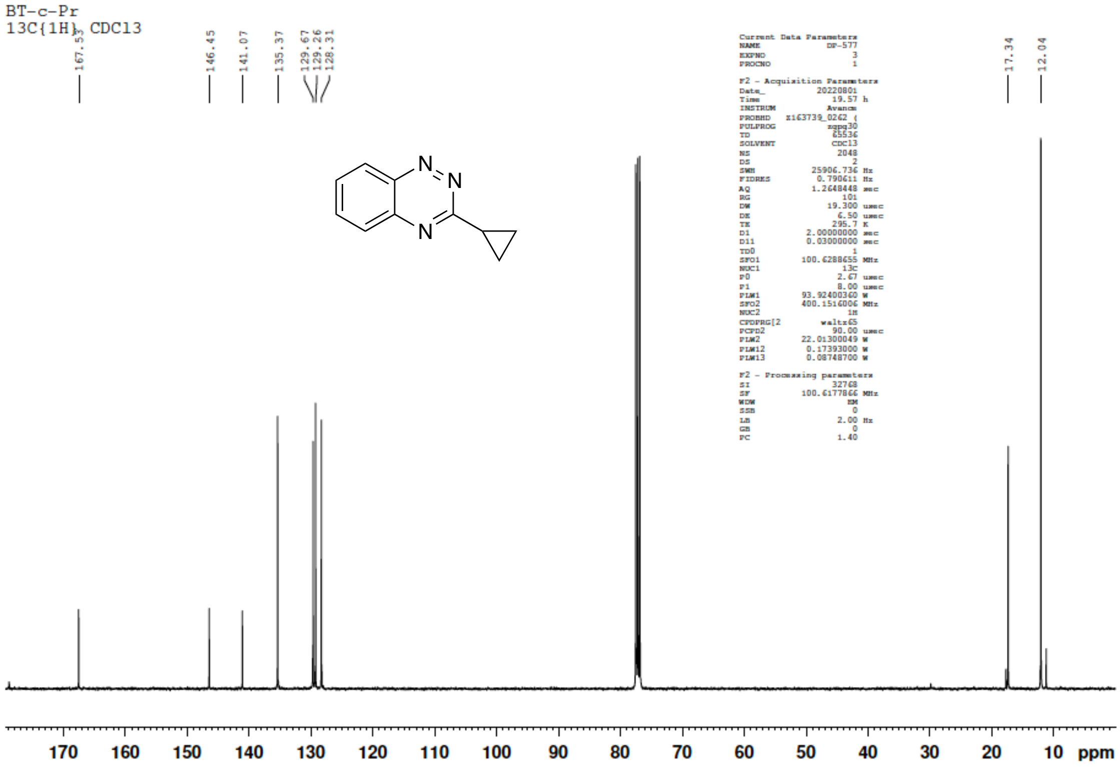

Figure S5.^{1}H NMR (400 MHz) and ^{13}C{^{1}H} NMR (100 MHz) spectra of **2m** ($CDCl_3$).

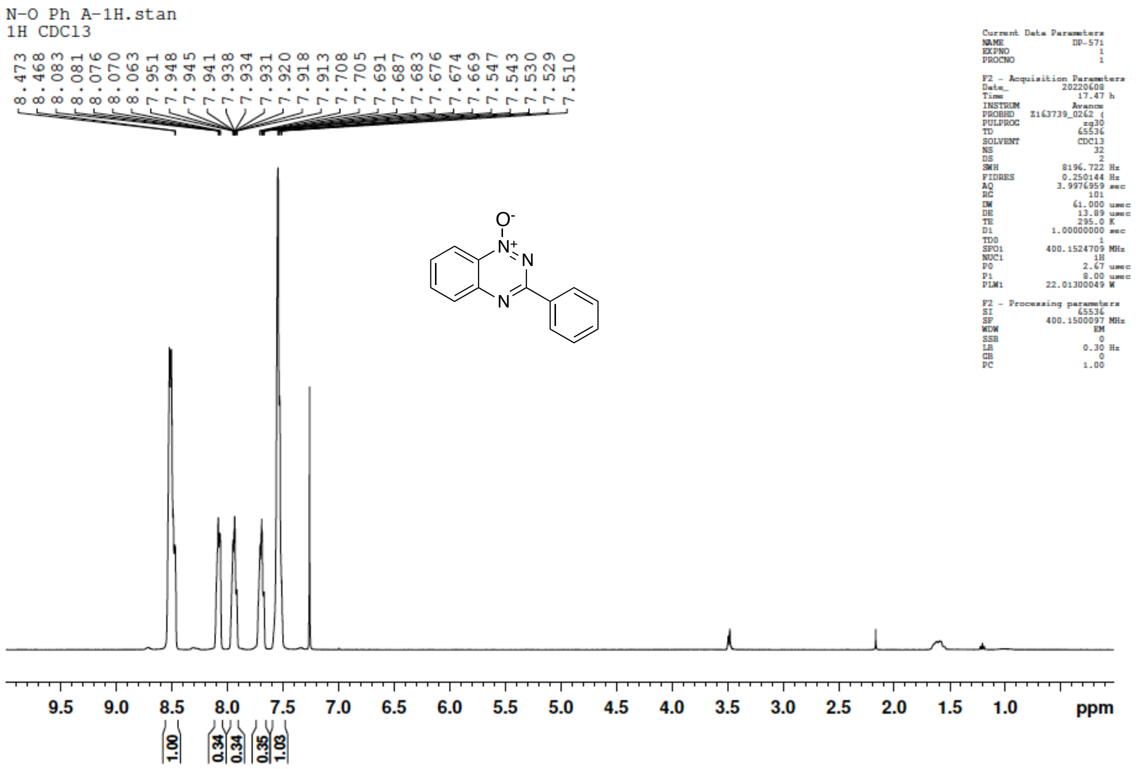

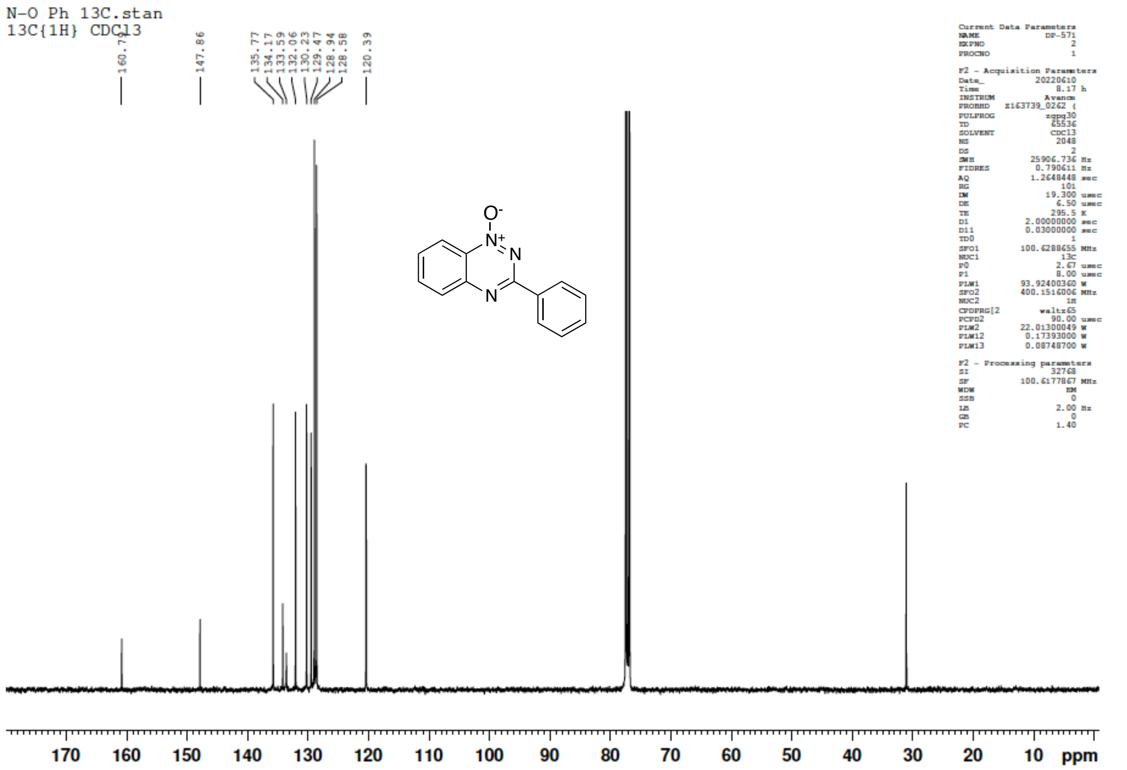

Figure S6.^{1}H NMR (400 MHz) and $^{13}C\{^{1}H\}$ NMR (100 MHz) spectra of **5a** ($CDCl_3$).

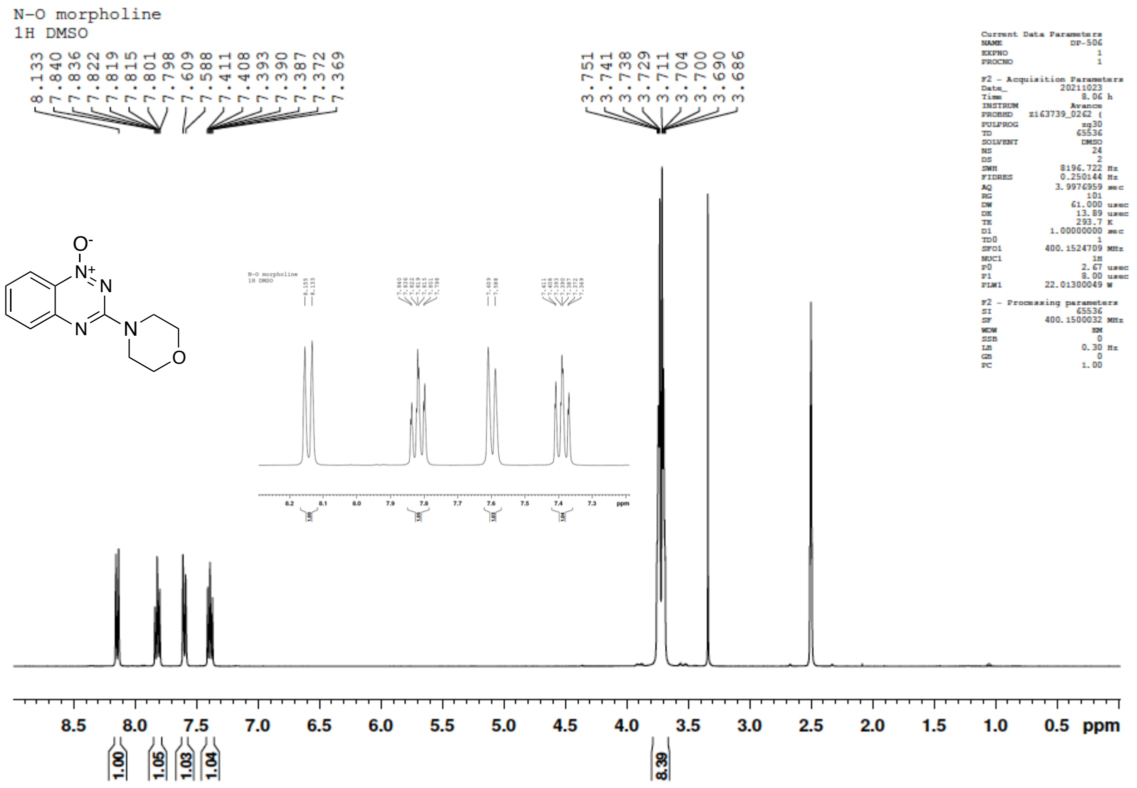

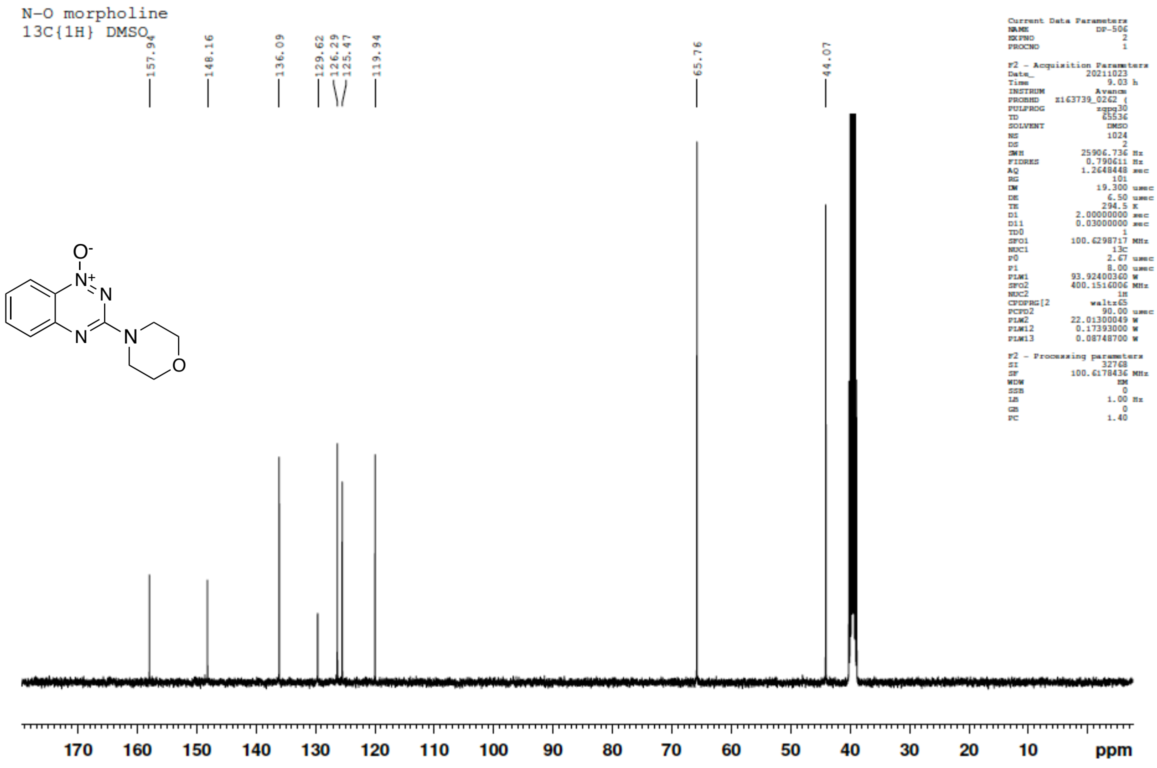

Figure S7. ^{1}H NMR (400 MHz) and ^{13}C{^{1}H} NMR (100 MHz) spectra of **5c** (DMSO-d_6).

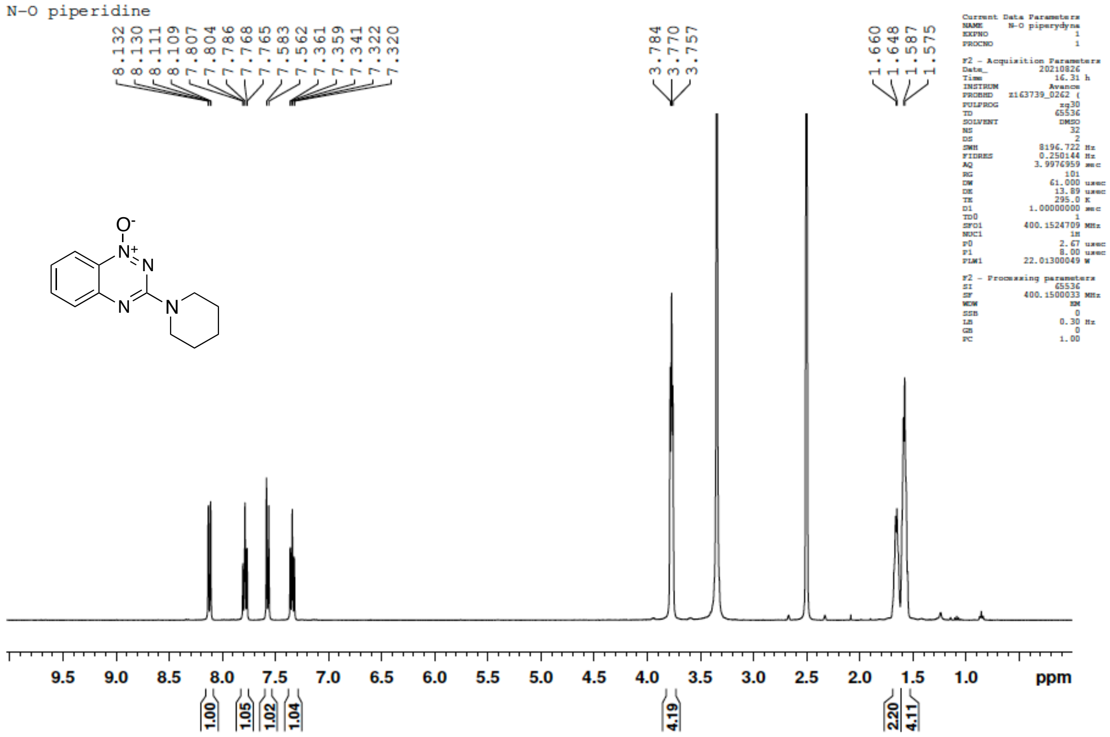

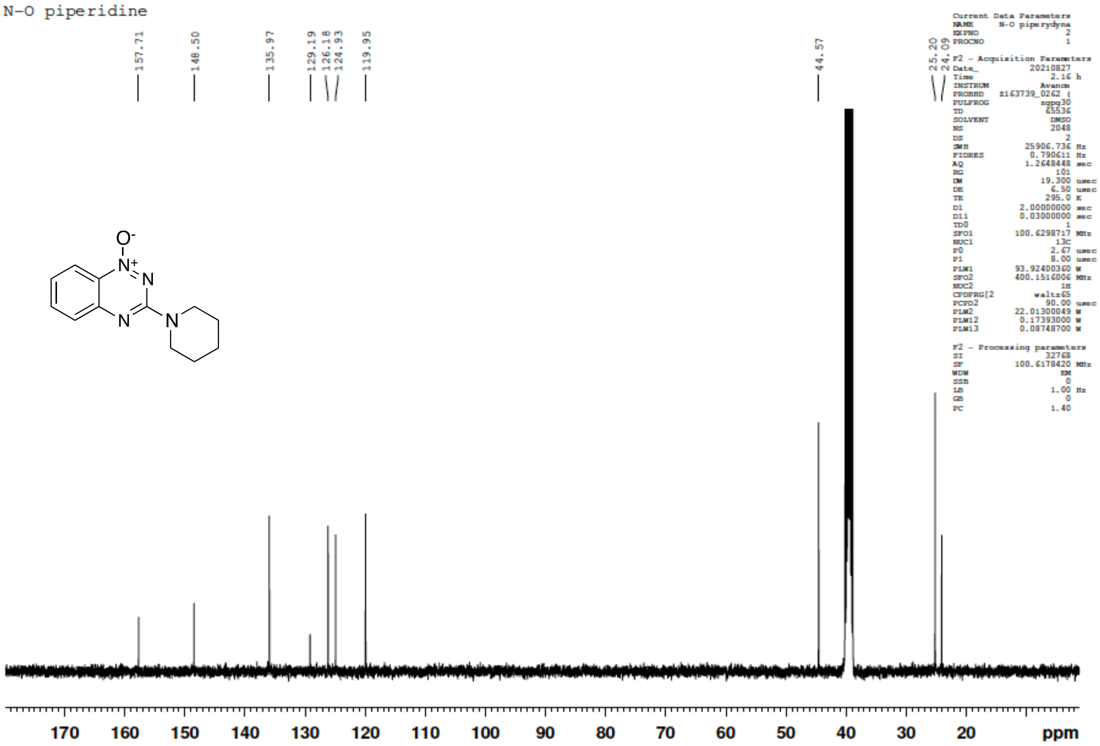

Figure S8.^{1}H NMR (400 MHz) and $^{13}C\{^{1}H\}$ NMR (100 MHz) spectra of **5g** (DMSO-d_6).

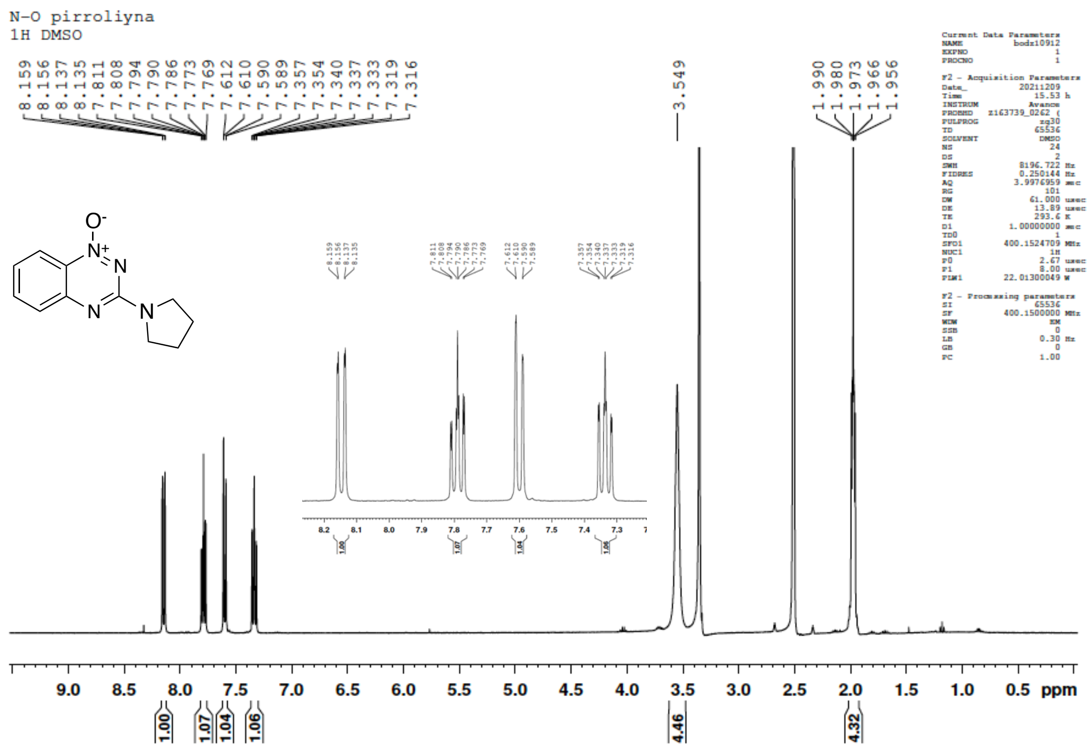

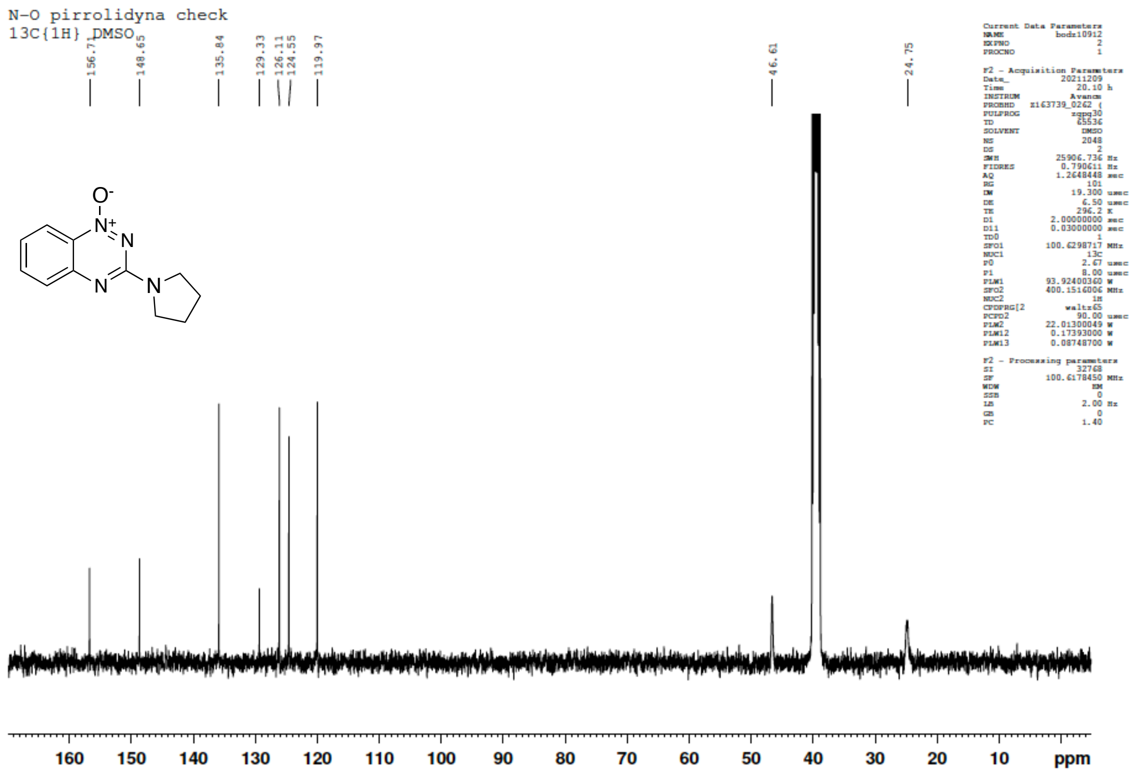

Figure S9. ^{1}H NMR (400 MHz) and ^{13}C{^{1}H} NMR (100 MHz) spectra of **5h** (DMSO-d_6).

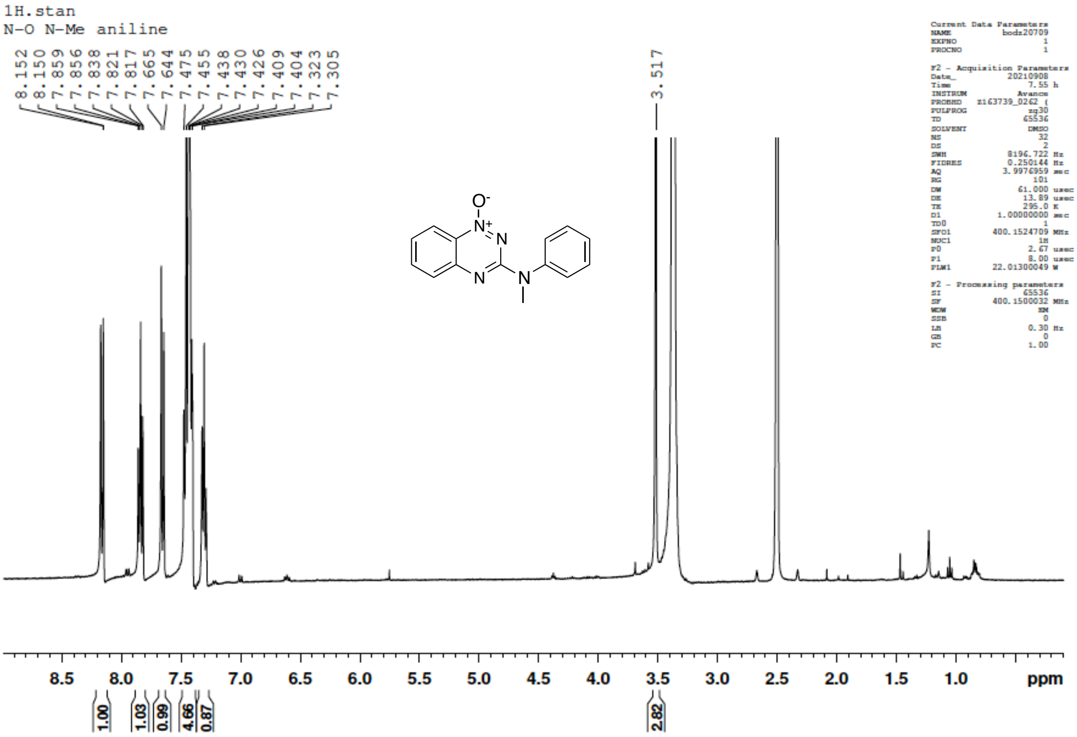

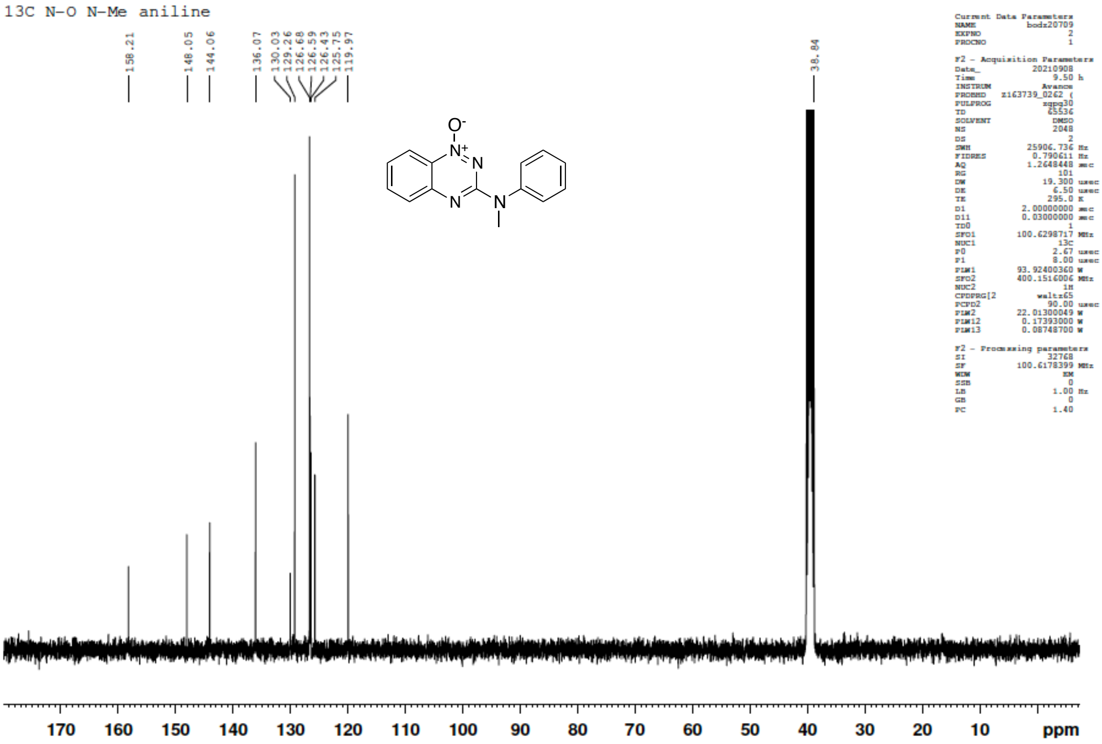

Figure S10. ^{1}H NMR (400 MHz) and ^{13}C{^{1}H} NMR (100 MHz) spectra of **5i** (DMSO-d_6).

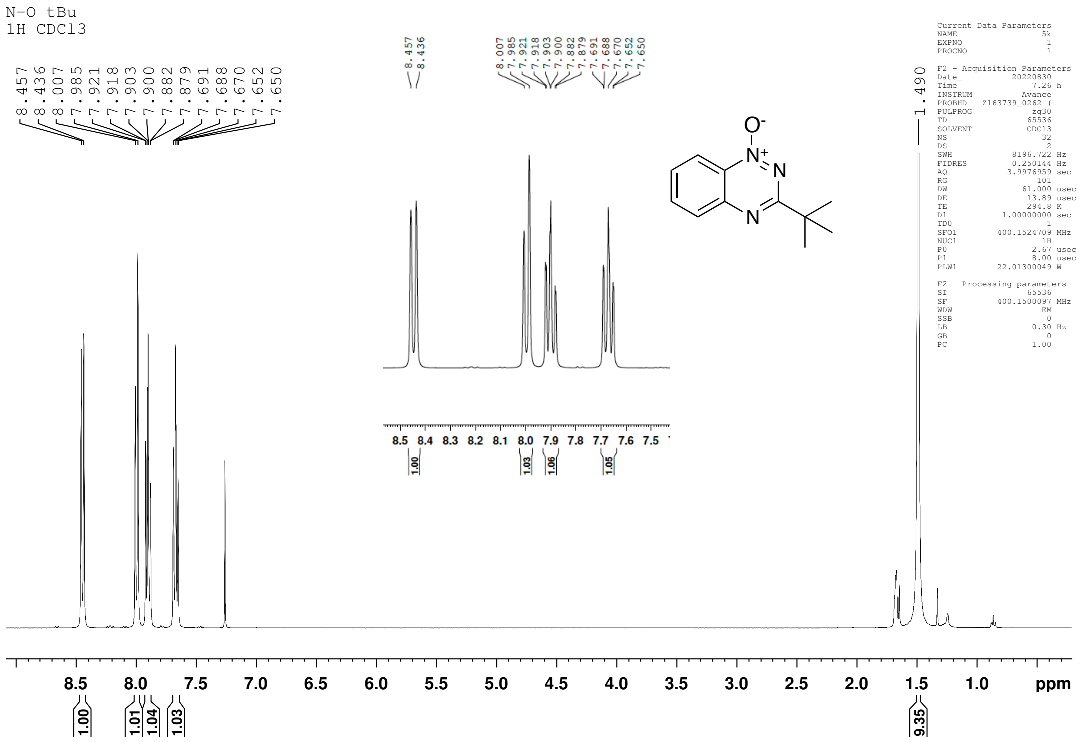

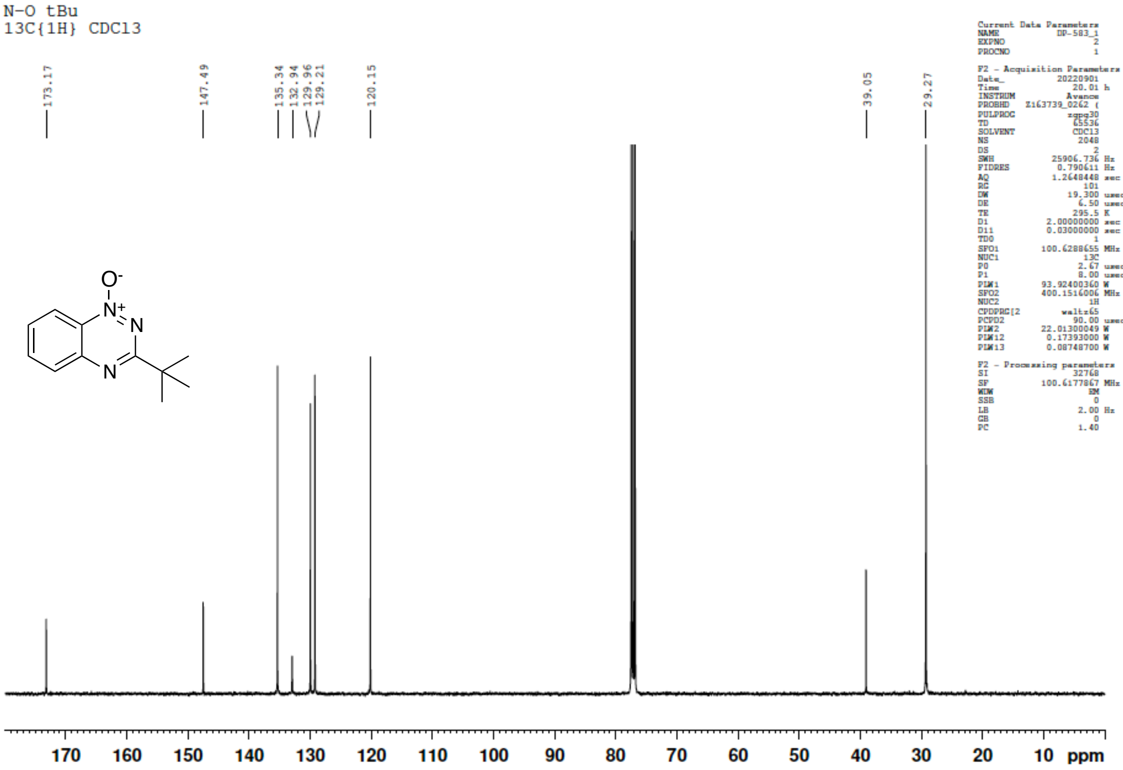

Figure S11. ^{1}H NMR (400 MHz) and ^{13}C{^{1}H} NMR (100 MHz) spectra of **5k** ($CDCl_3$).

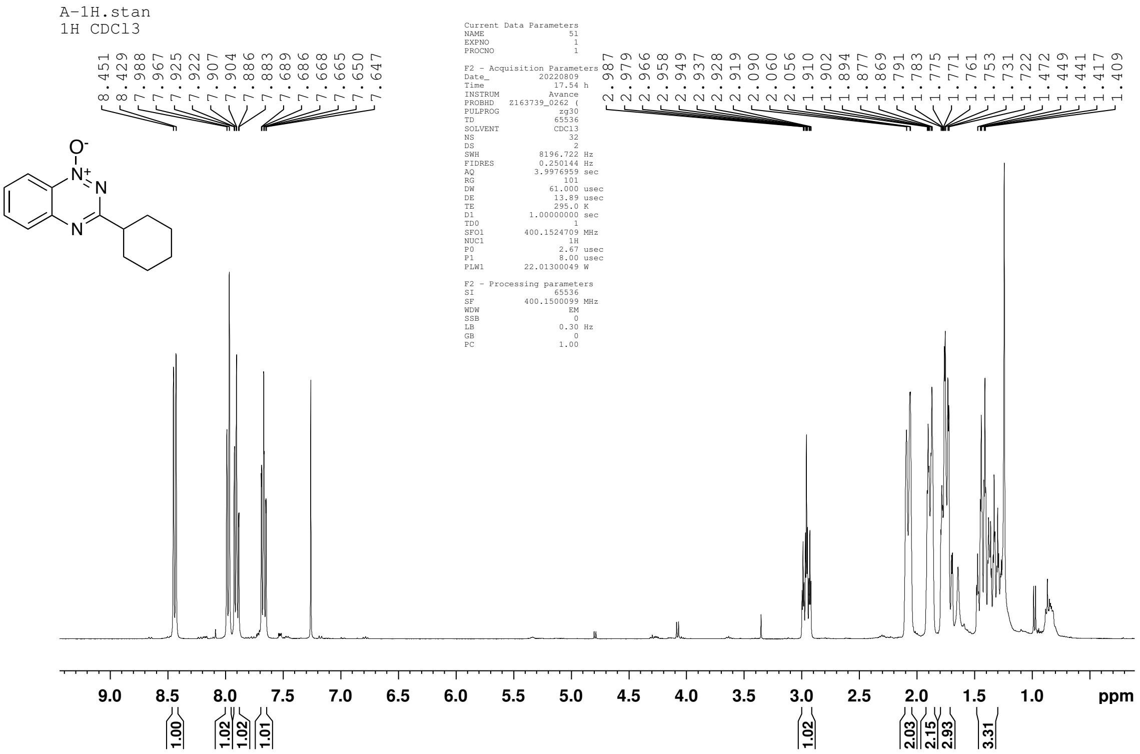

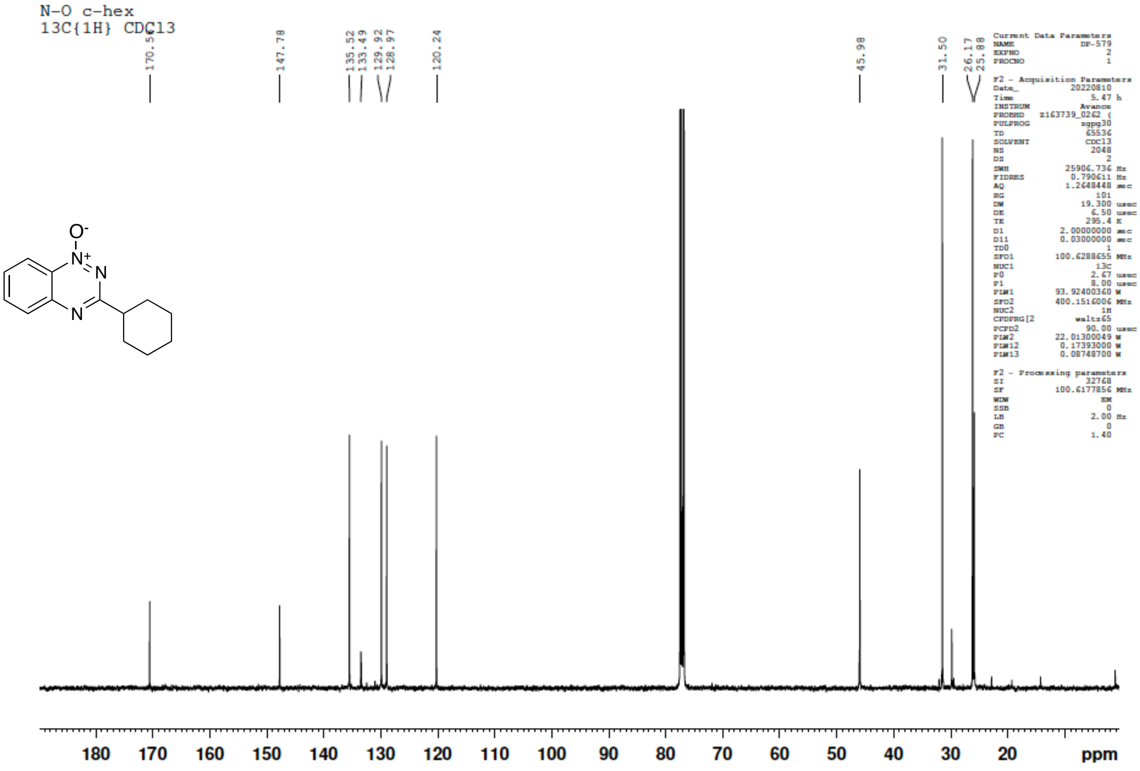

Figure S12. ^{1}H NMR (400 MHz) and $^{13}C\{^{1}H\}$ NMR (100 MHz) spectra of **5l** ($CDCl_3$).

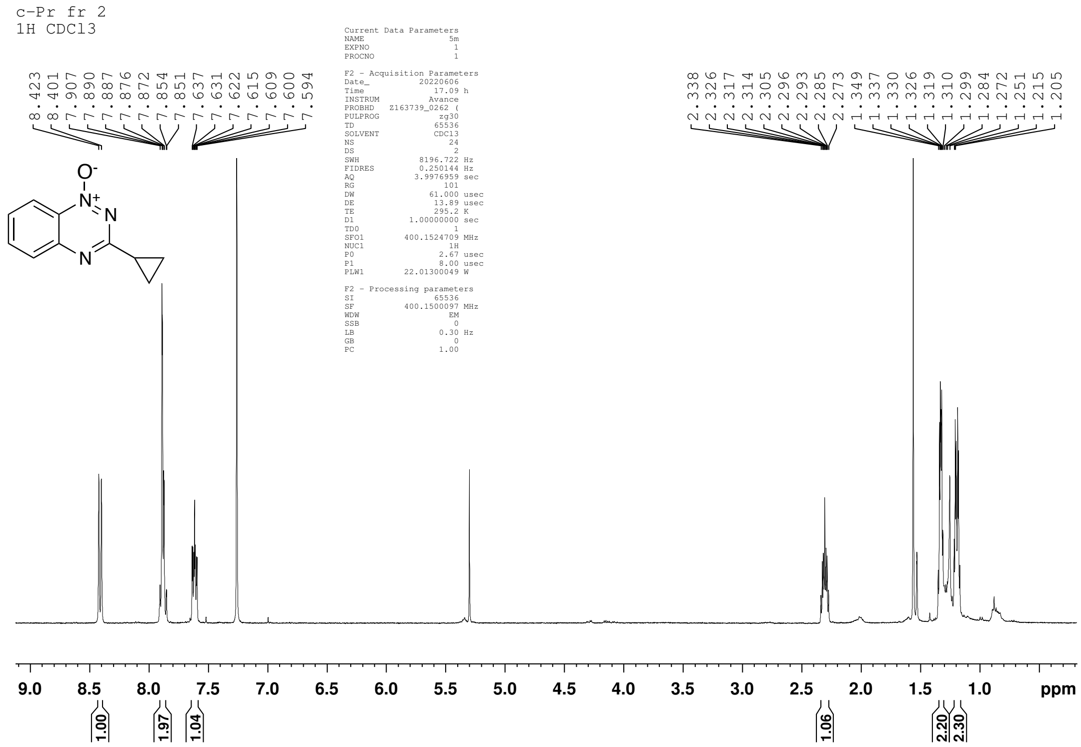

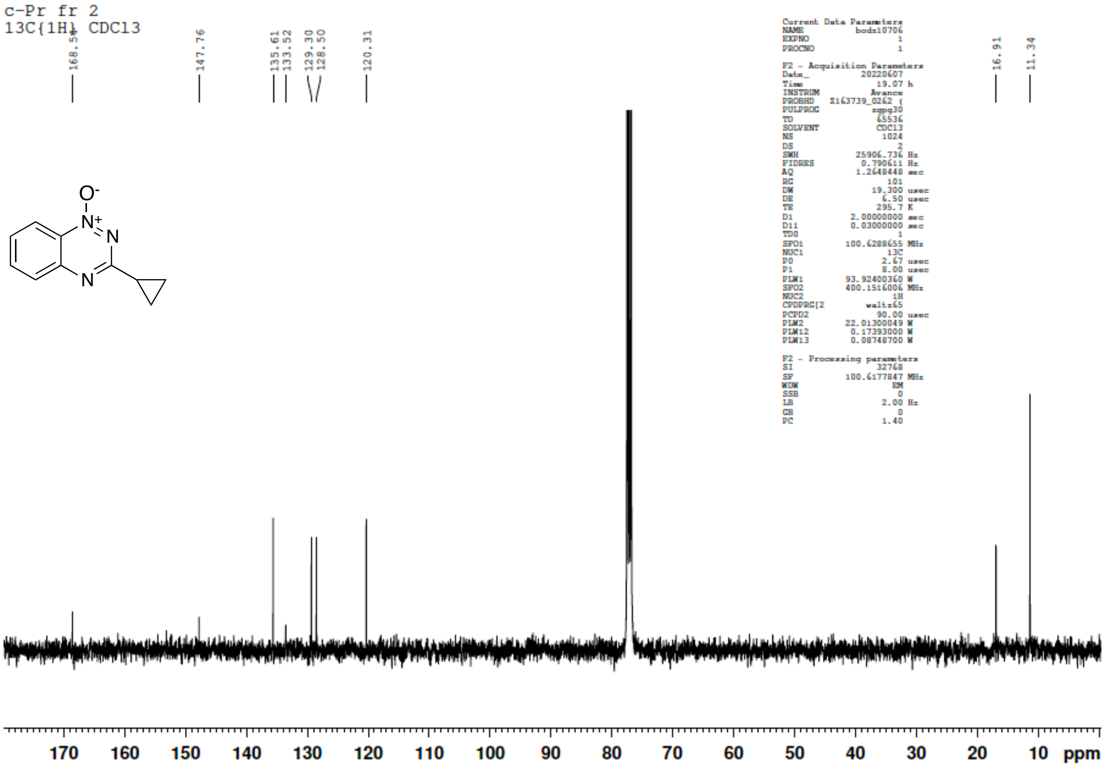

Figure S13. ^{1}H NMR (400 MHz) and ^{13}C{^{1}H} NMR (100 MHz) spectra of **5m** ($CDCl_3$).

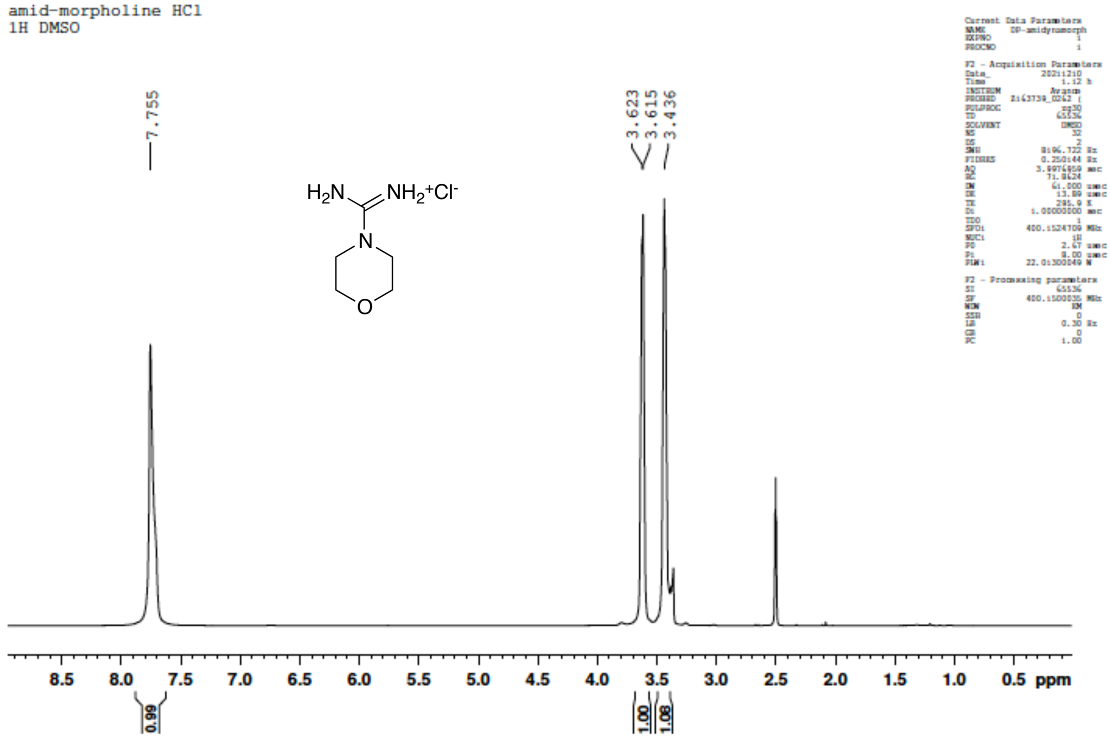

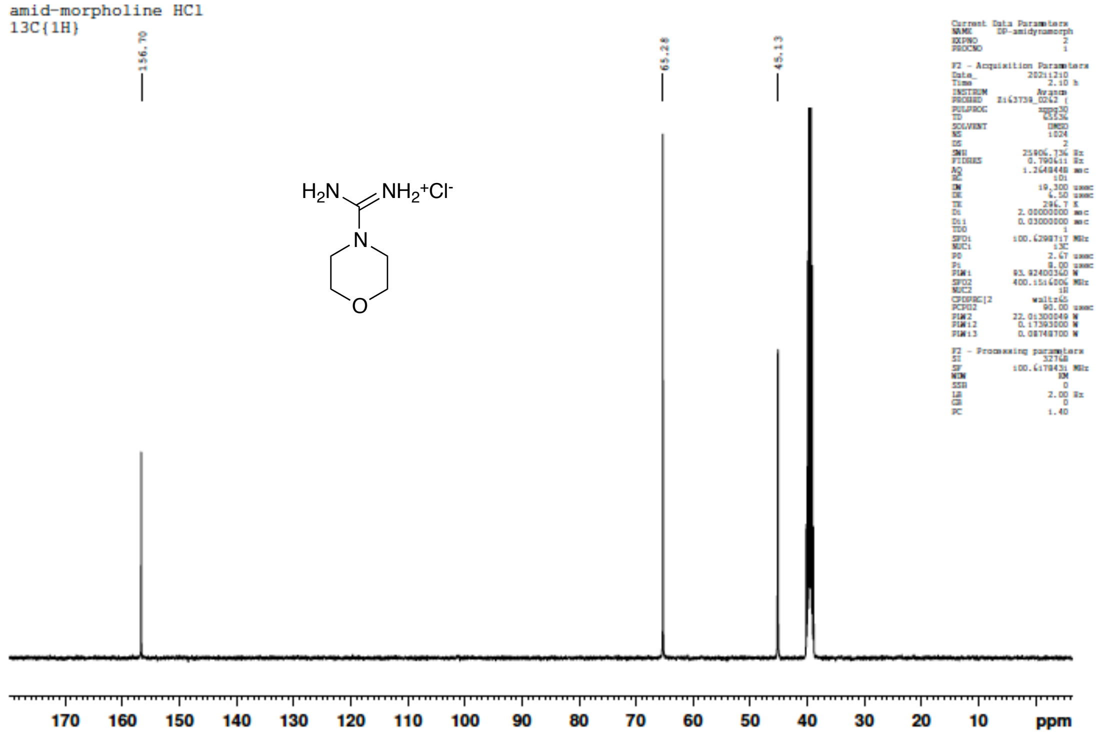

Figure S14. ^{1}H NMR (400 MHz) and ^{13}C{^{1}H} NMR (100 MHz) spectra of **6c** (DMSO-d_6).

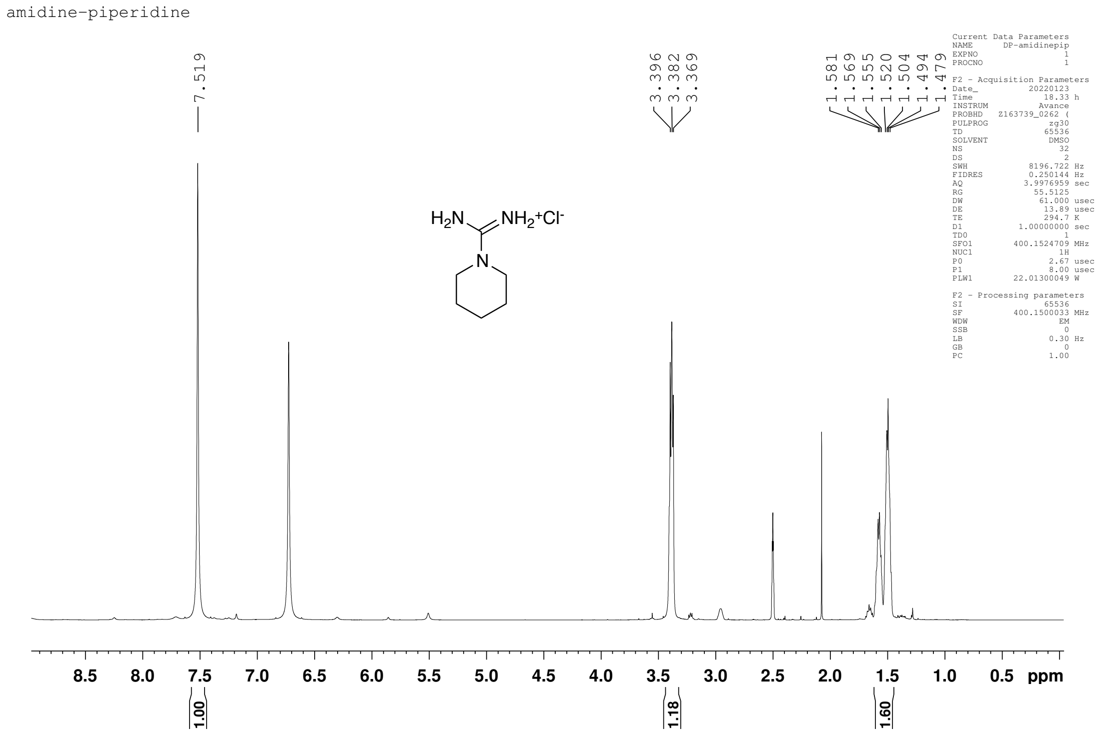

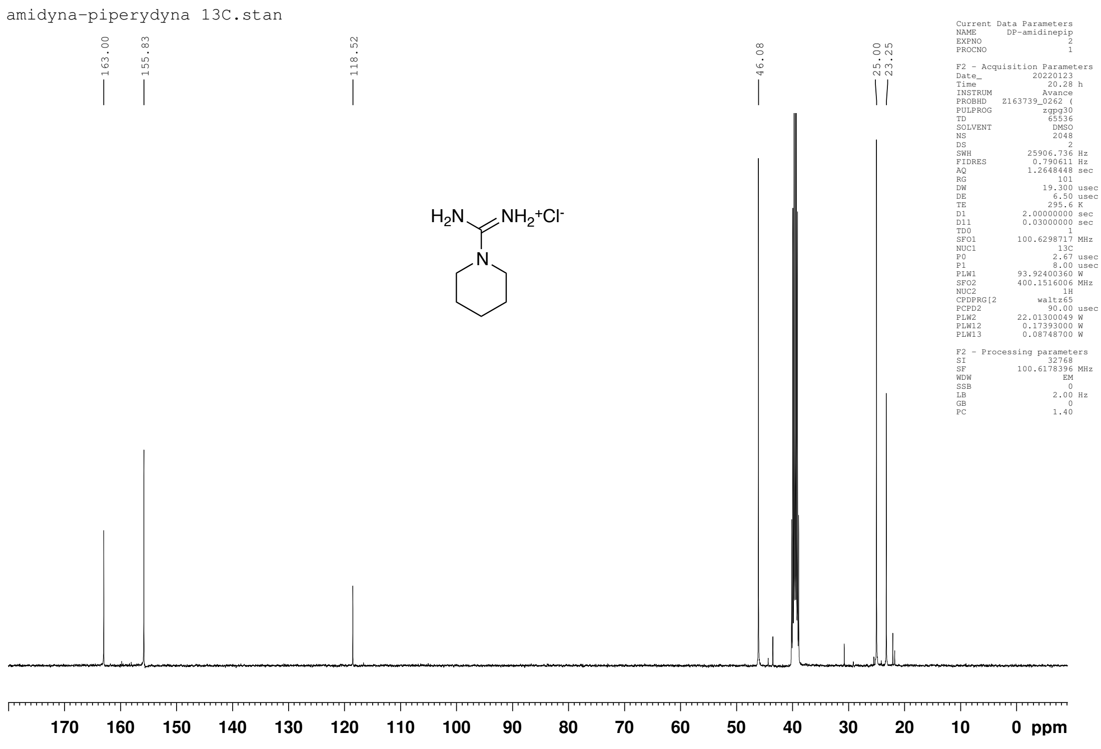

Figure S15.^{1}H NMR (400 MHz) and $^{13}C\{^{1}H\}$ NMR (100 MHz) spectra of **6g** (DMSO-d_6).

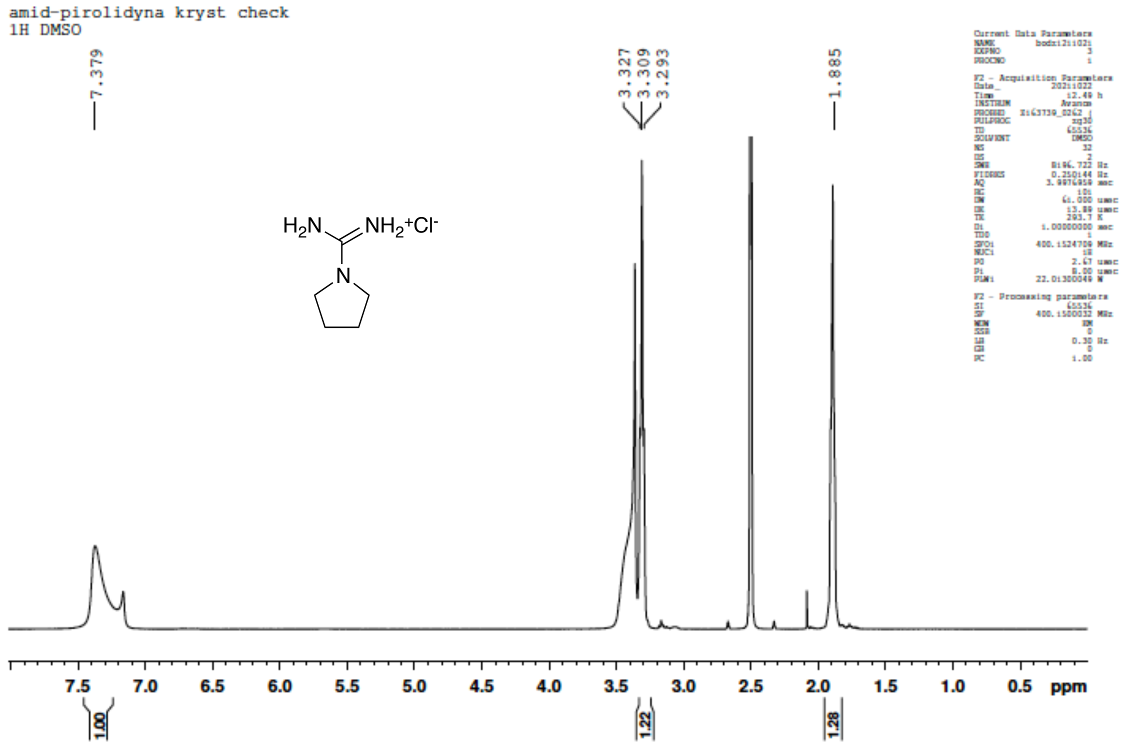

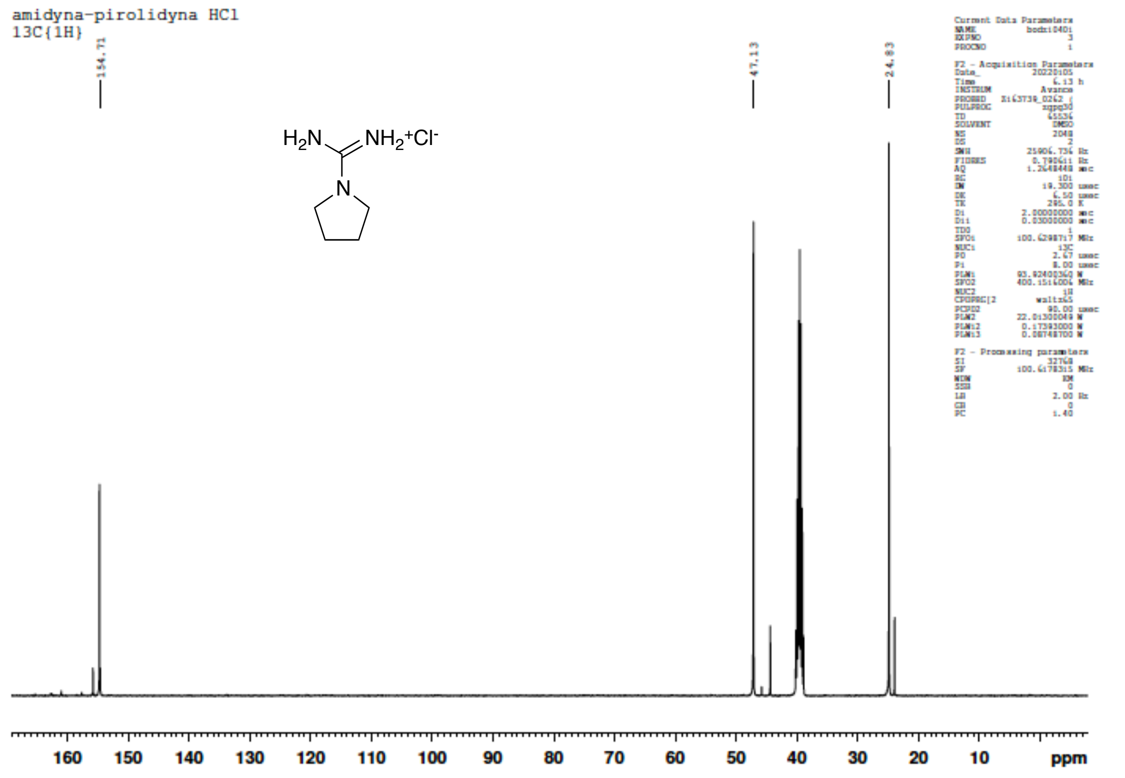

Figure S16. ^{1}H NMR (400 MHz) and $^{13}C\{^1H\}$ NMR (100 MHz) spectra of **6h** (DMSO-d_6).

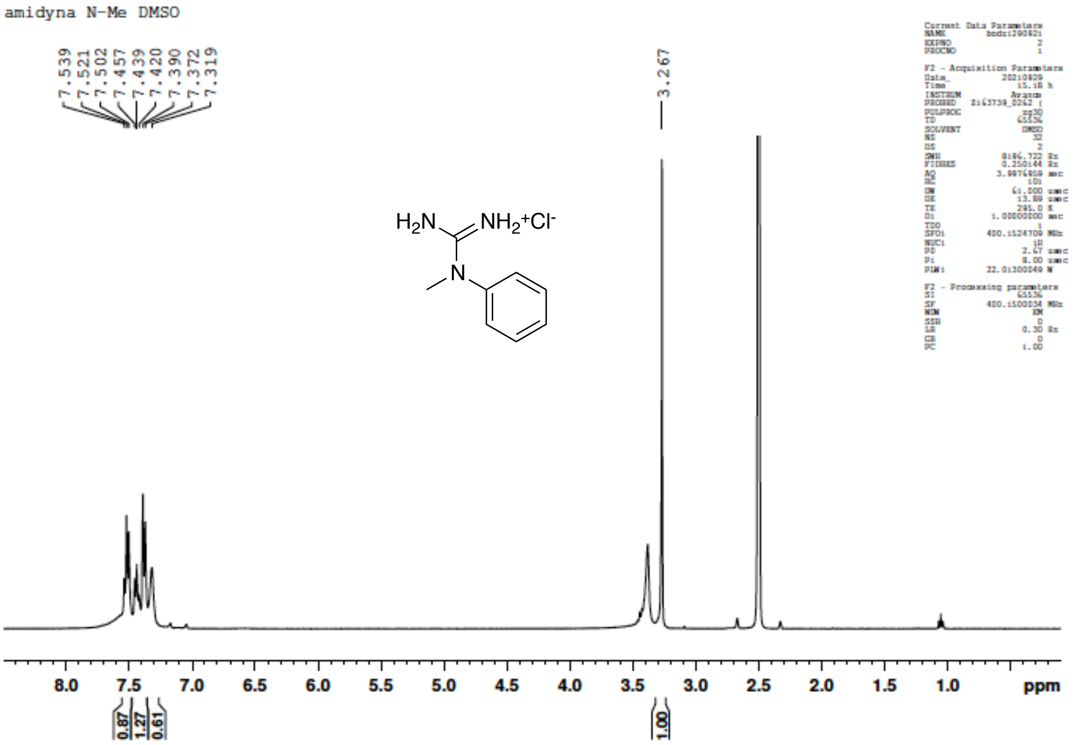

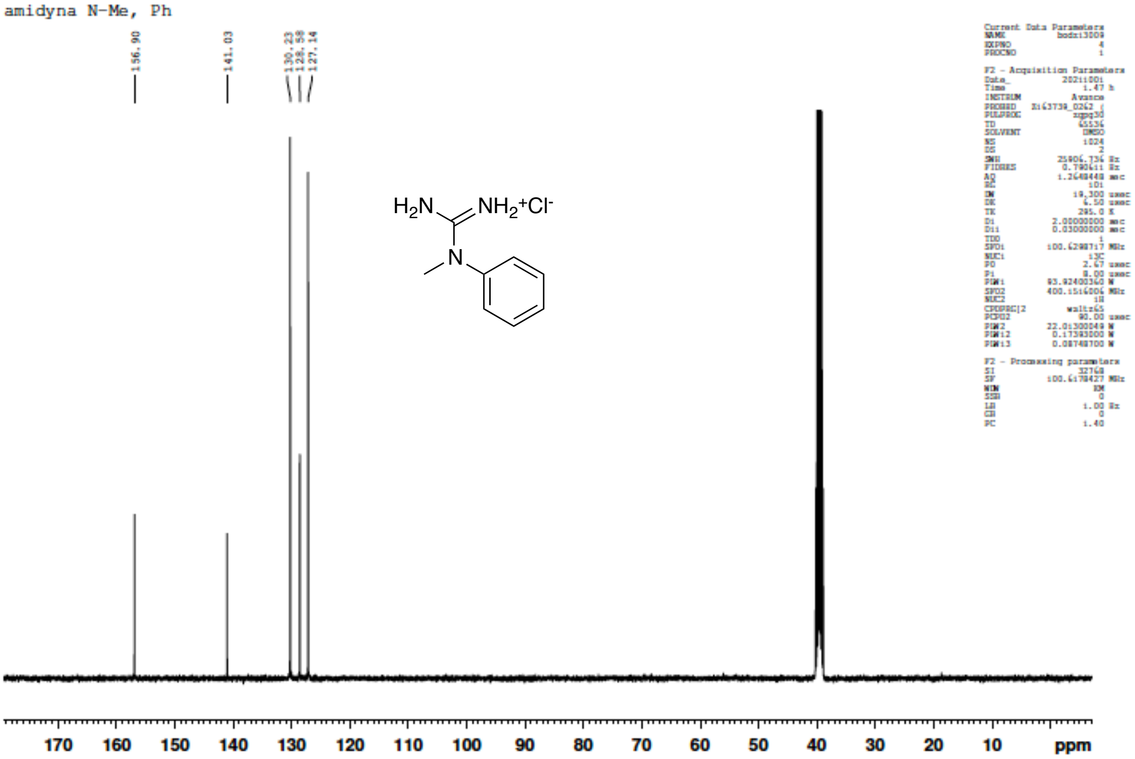

Figure S17. ^{1}H NMR (400 MHz) and $^{13}C\{^{1}H\}$ NMR (100 MHz) spectra of **6i** (DMSO-d_6).

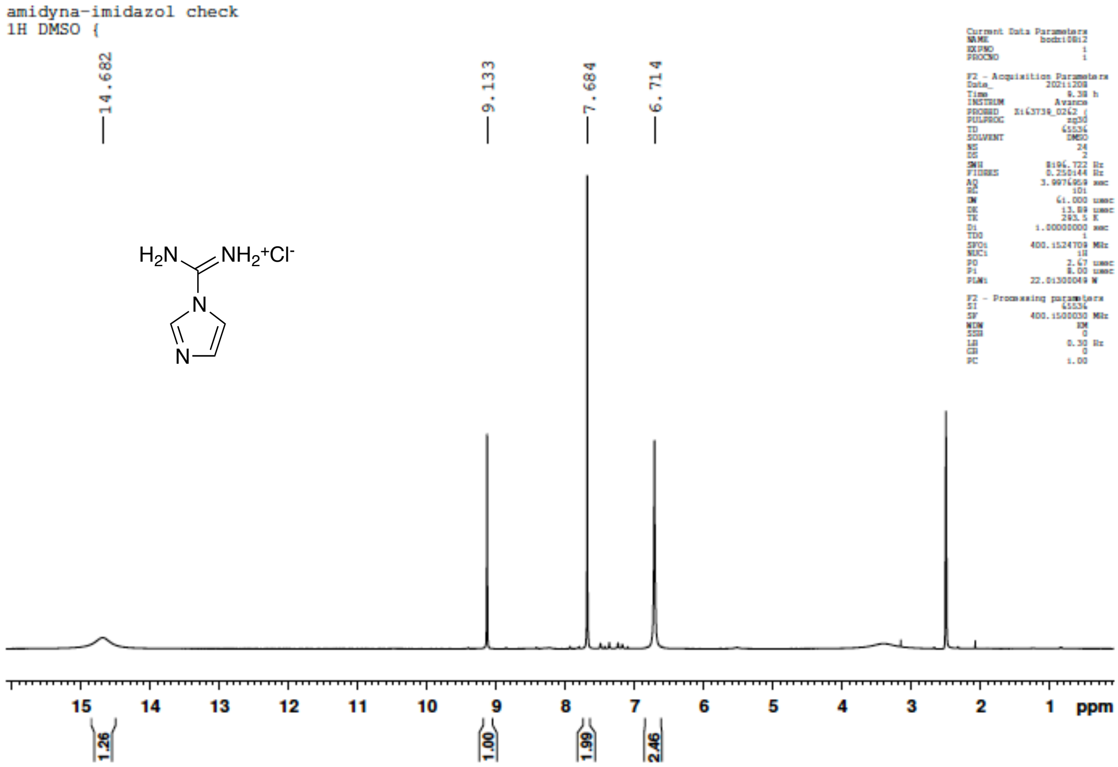

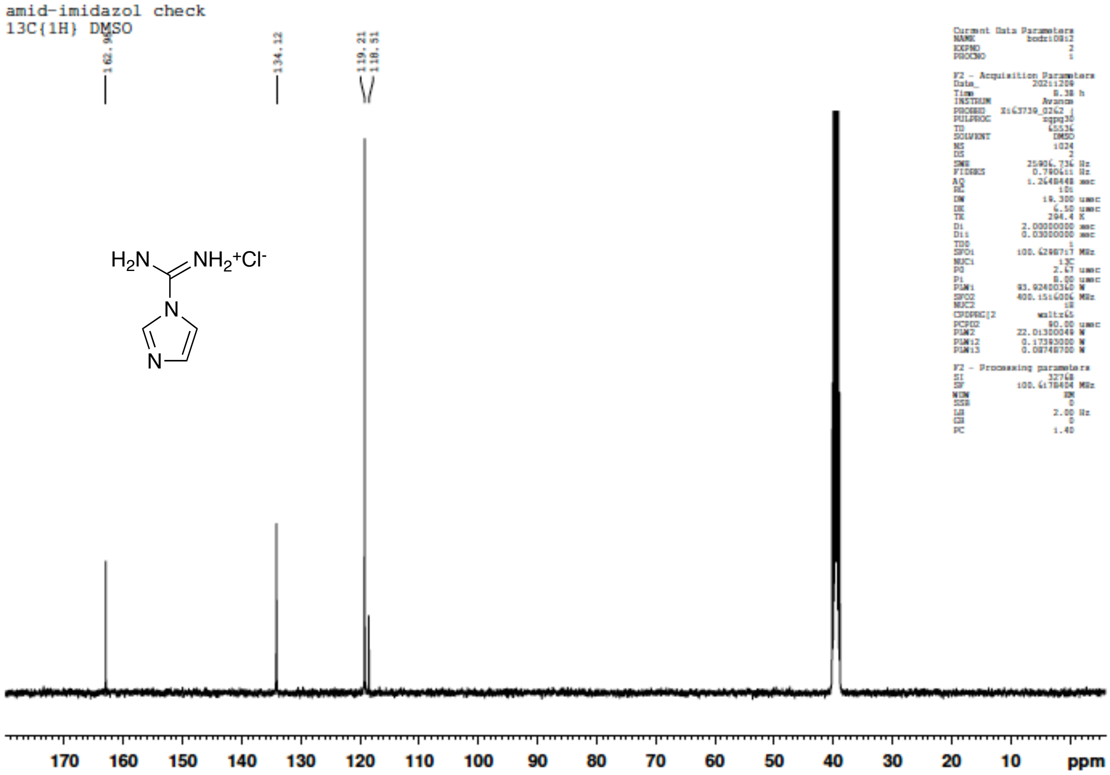

Figure S18. ^{1}H NMR (400 MHz) and $^{13}C\{^{1}H\}$ NMR (100 MHz) spectra of **6j** (DMSO-d_6).

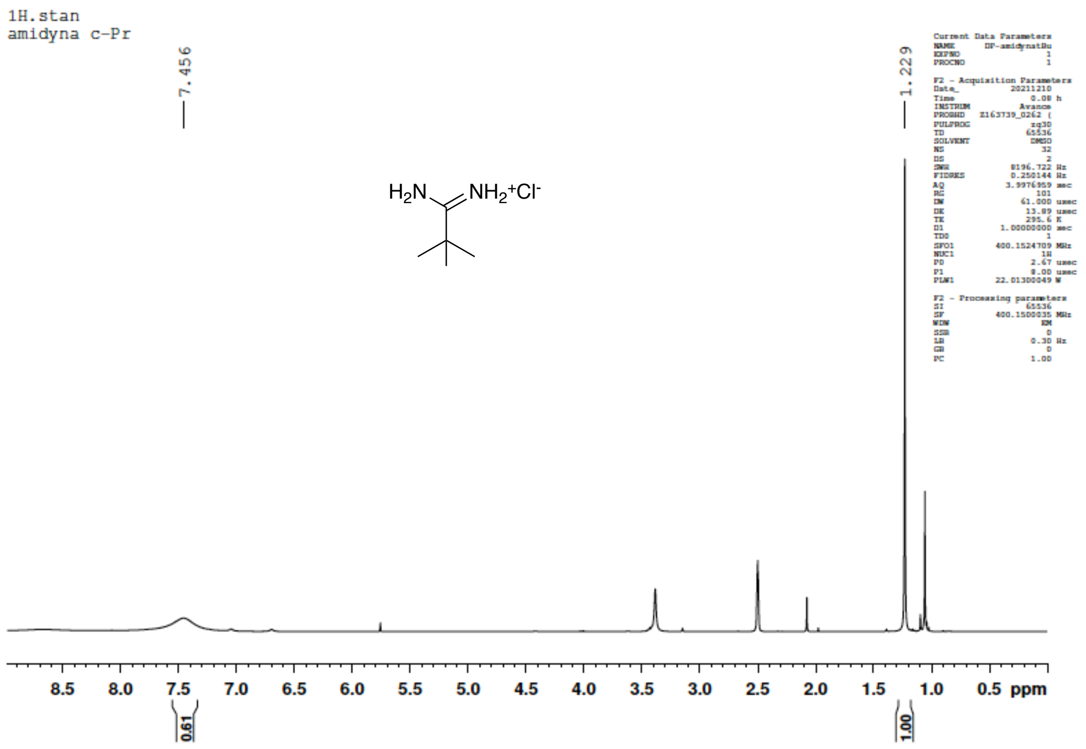

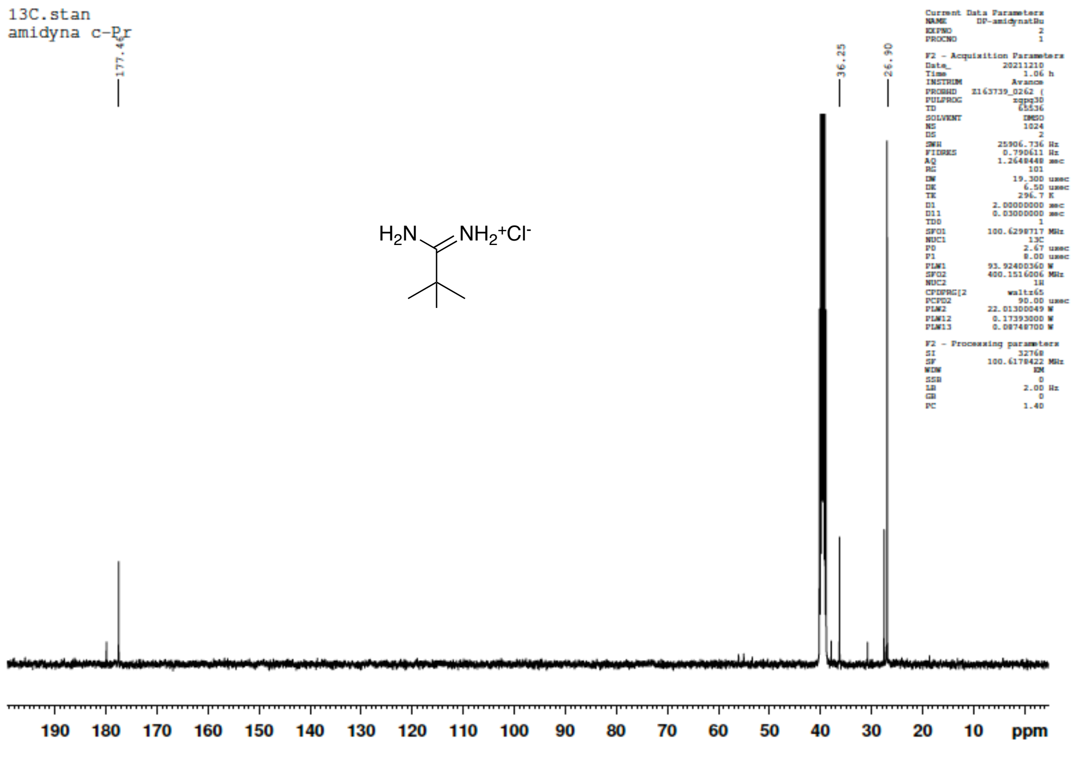

Figure S19. ^{1}H NMR (400 MHz) and ^{13}C{^{1}H} NMR (100 MHz) spectra of **7k** (DMSO-d_6).

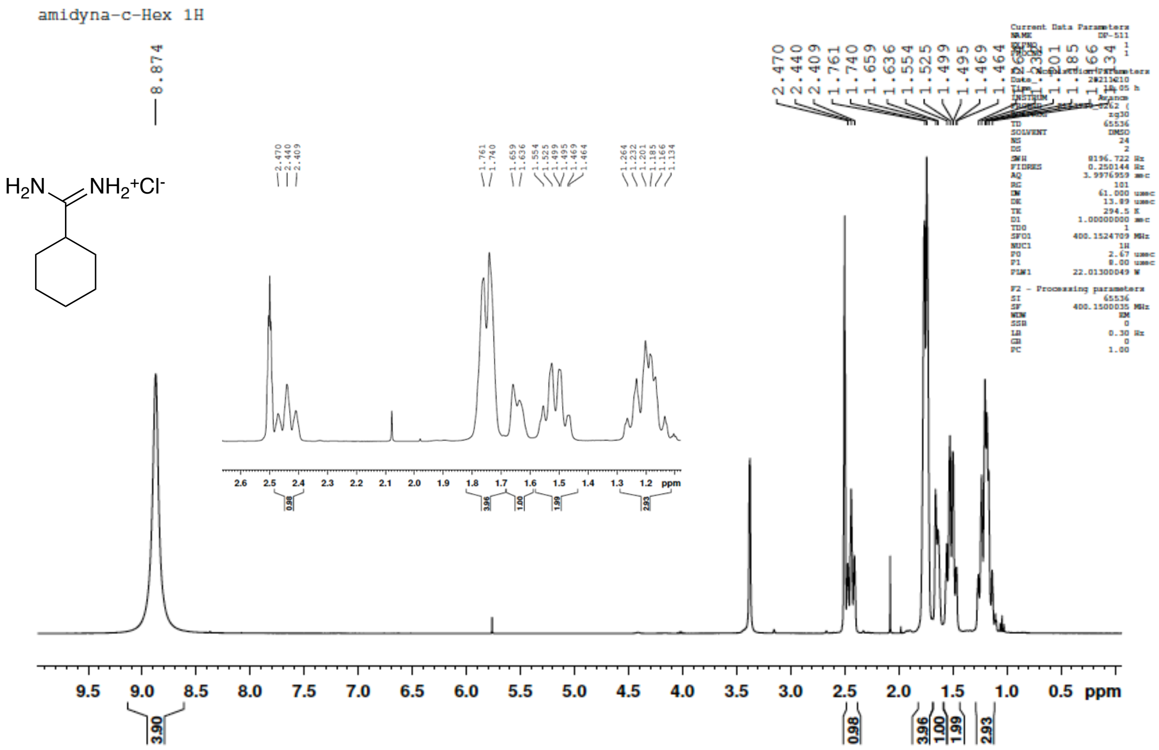

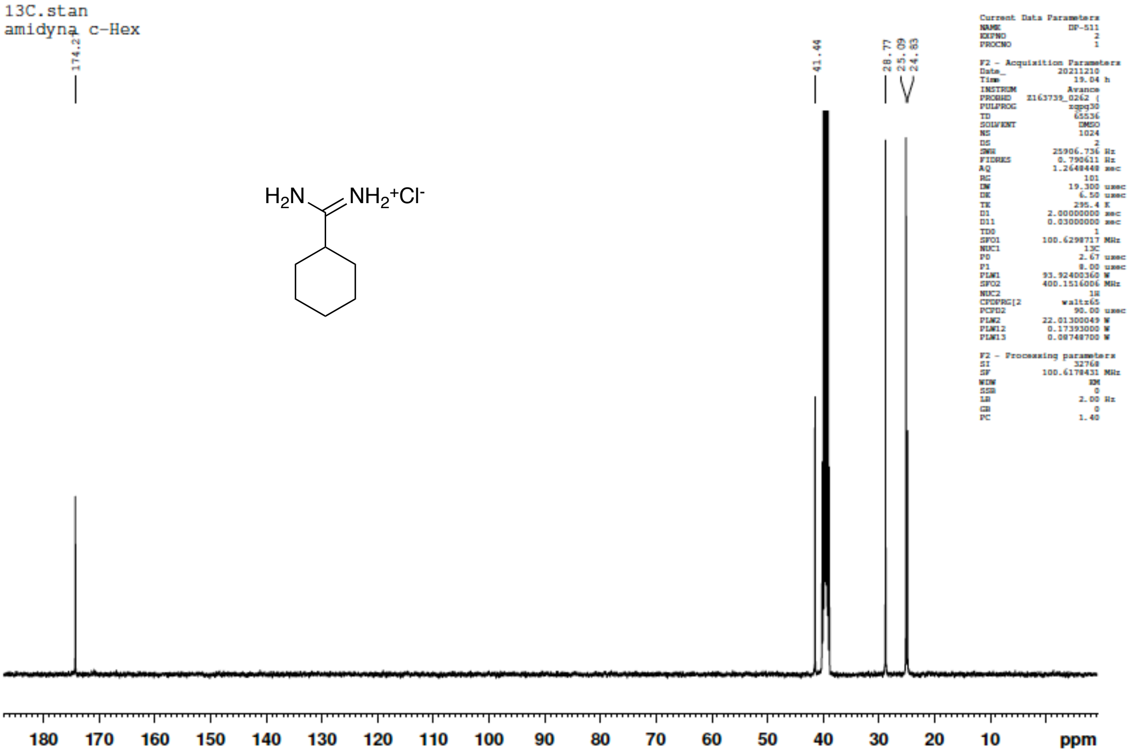

Figure S20. ^{1}H NMR (400 MHz) and $^{13}C\{^{1}H\}$ NMR (100 MHz) spectra of **7l** (DMSO-d_6).

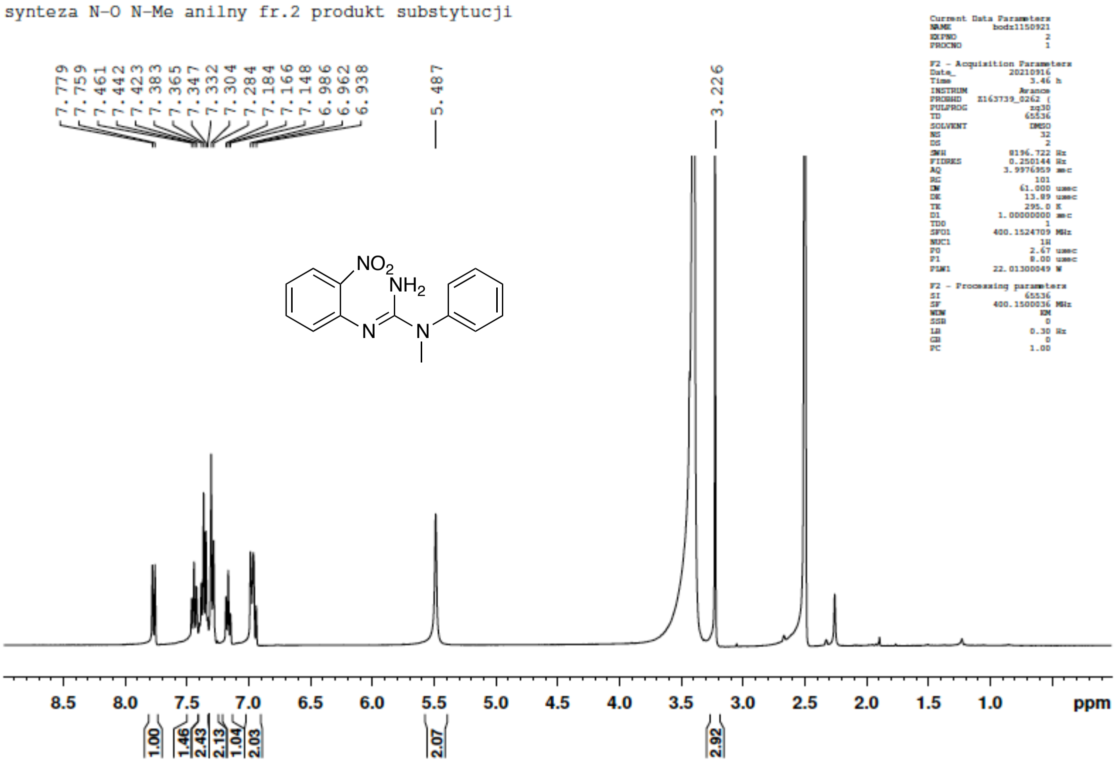

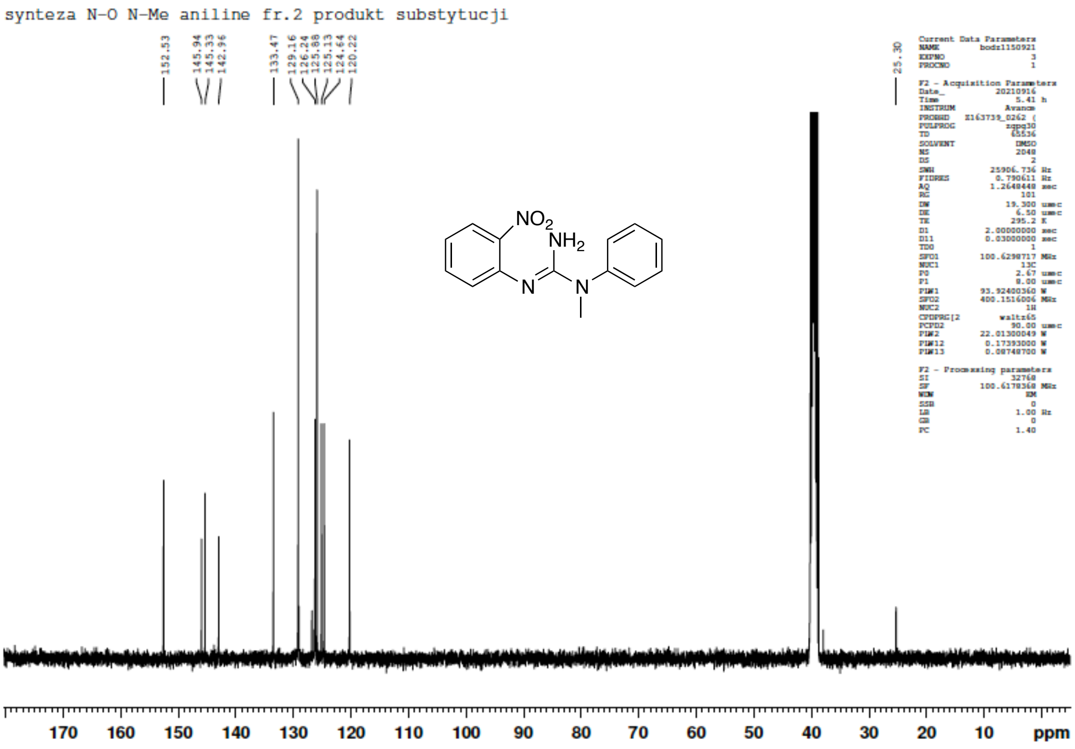

Figure S21. ^{1}H NMR (400 MHz) and ^{13}C{^{1}H} NMR (100 MHz) spectra of **12i** (DMSO-d_6).

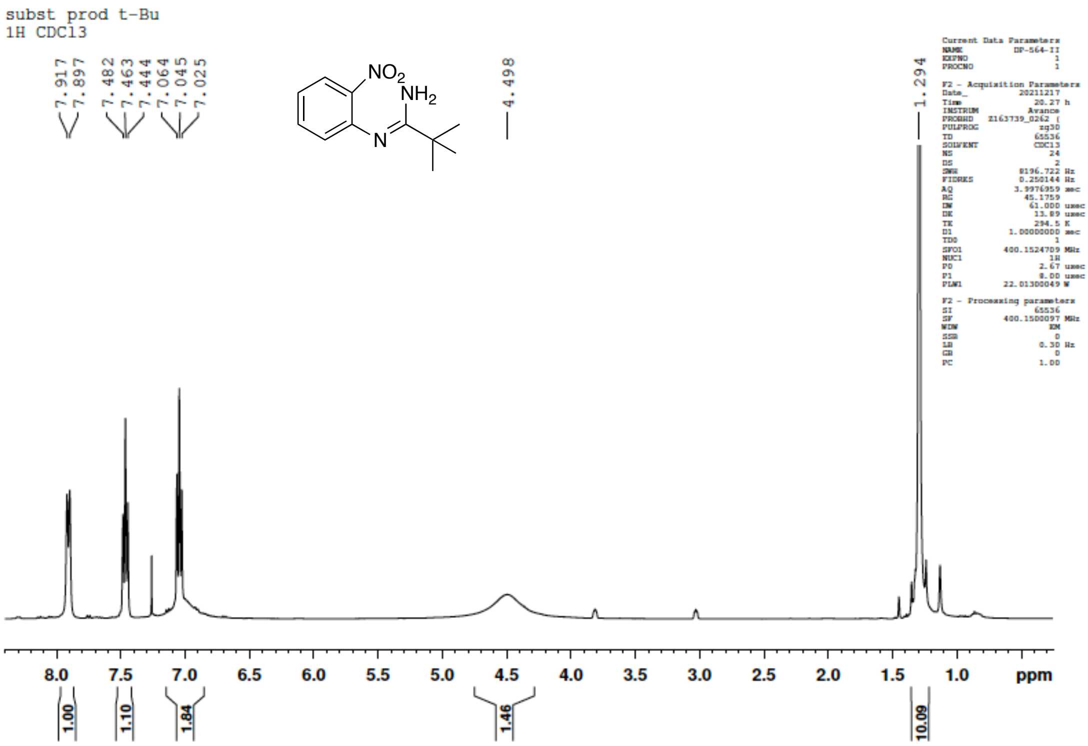

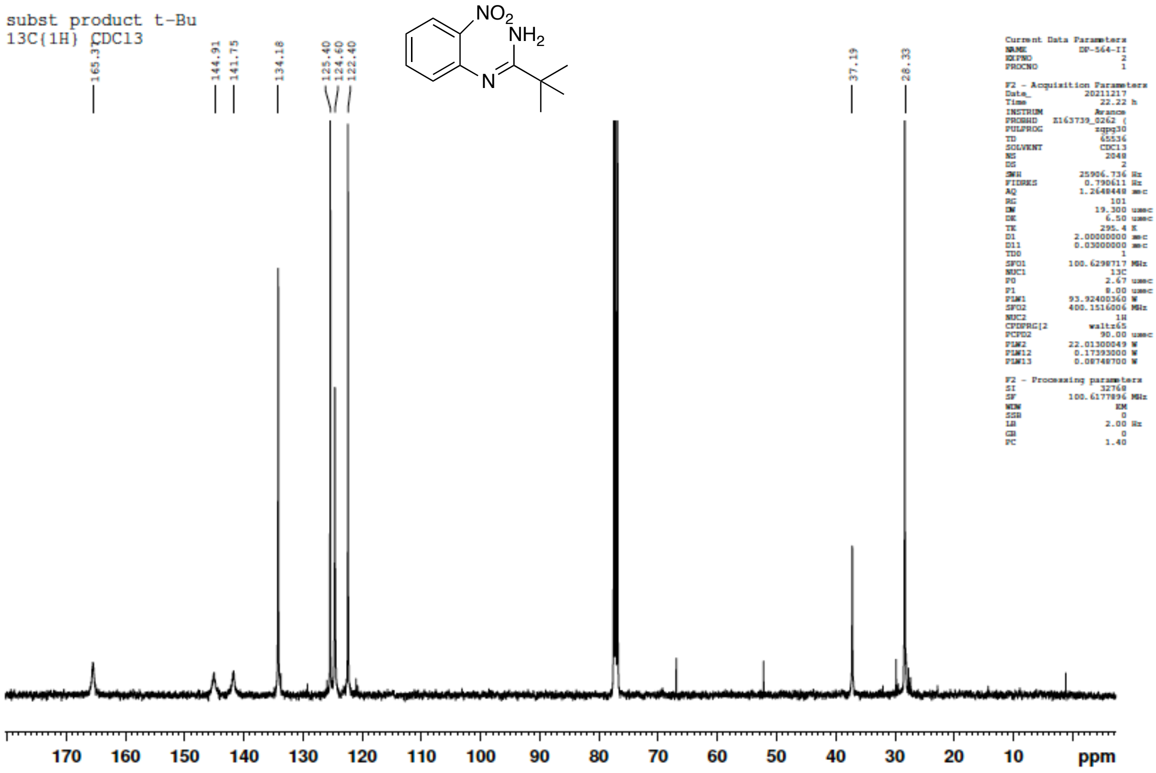

Figure S22. ^{1}H NMR (400 MHz) and ^{13}C{^{1}H} NMR (100 MHz) spectra of **12k** ($CDCl_3$).

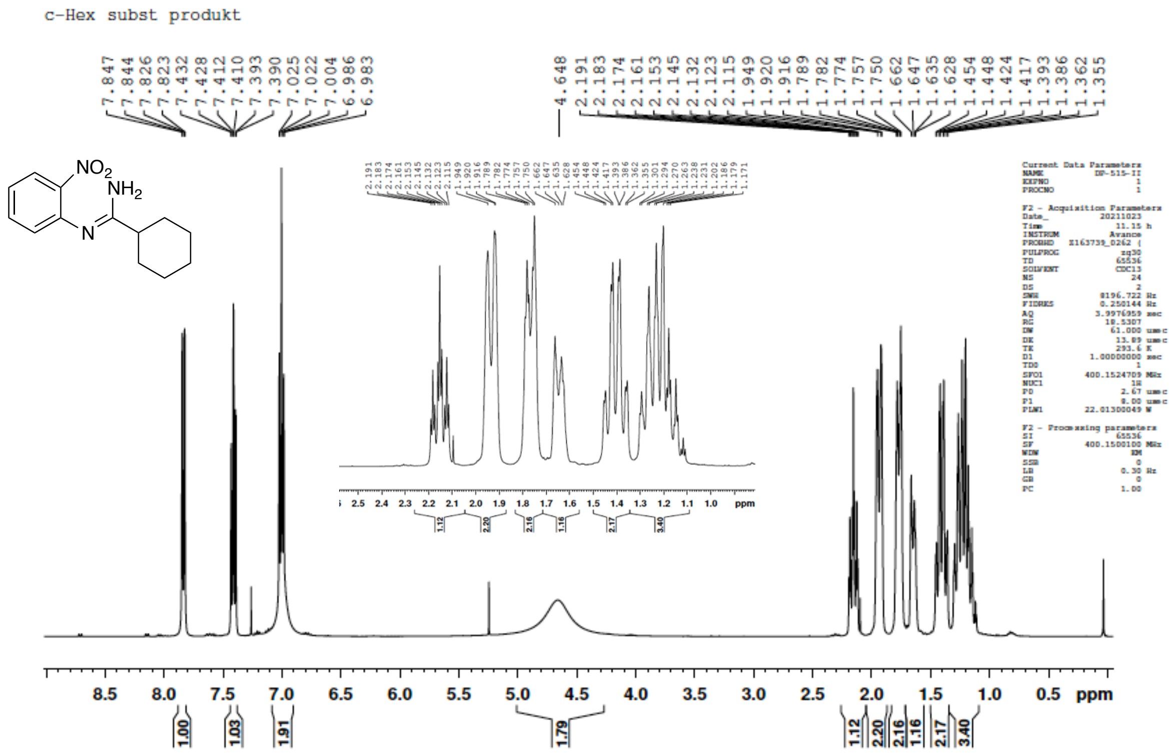

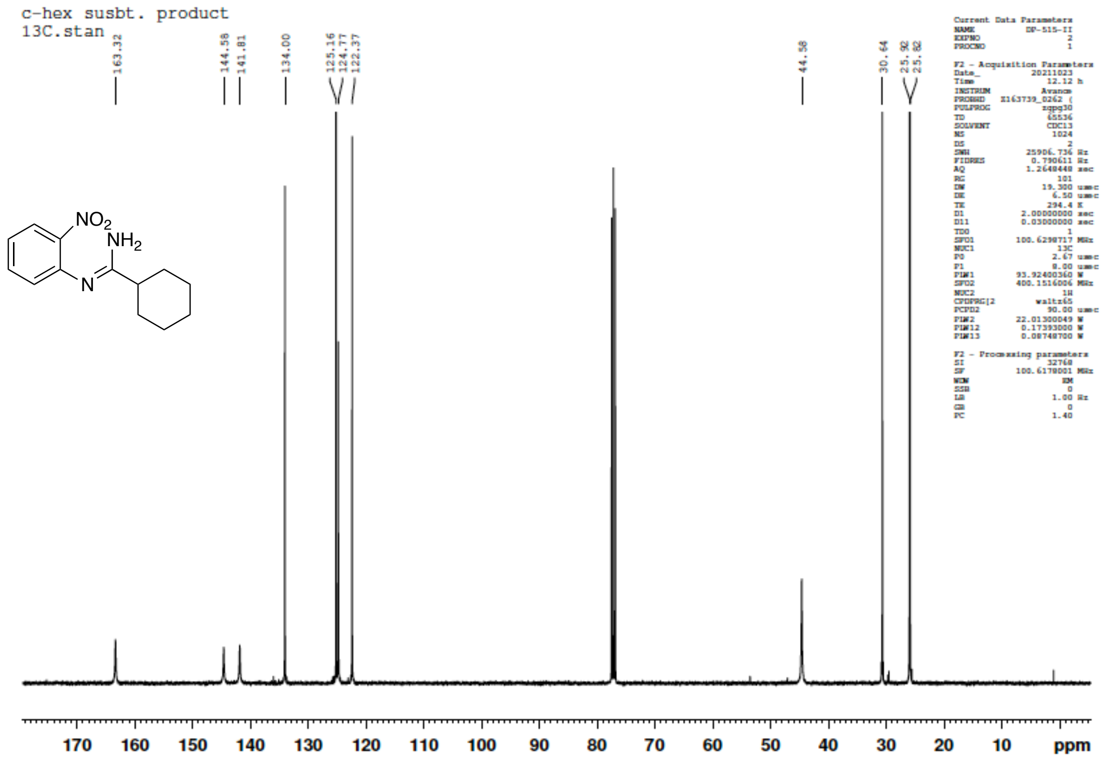

Figure S23. ^{1}H NMR (400 MHz) and ^{13}C{^{1}H} NMR (100 MHz) spectra of **12l** ($CDCl_3$).

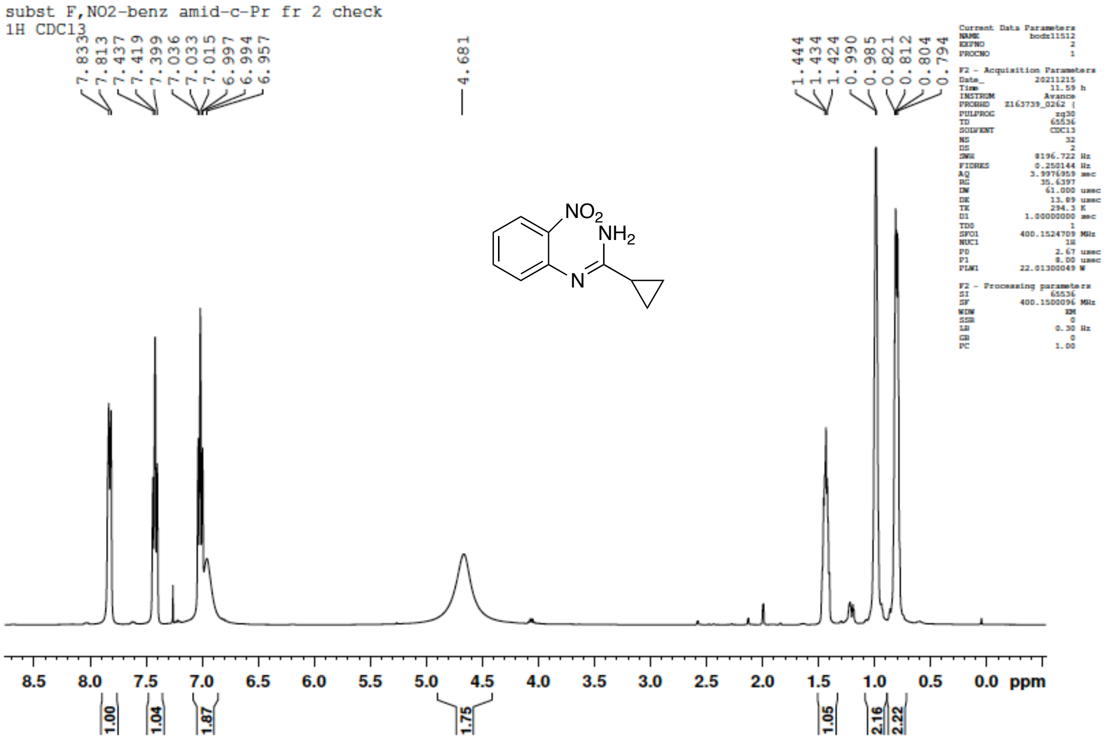

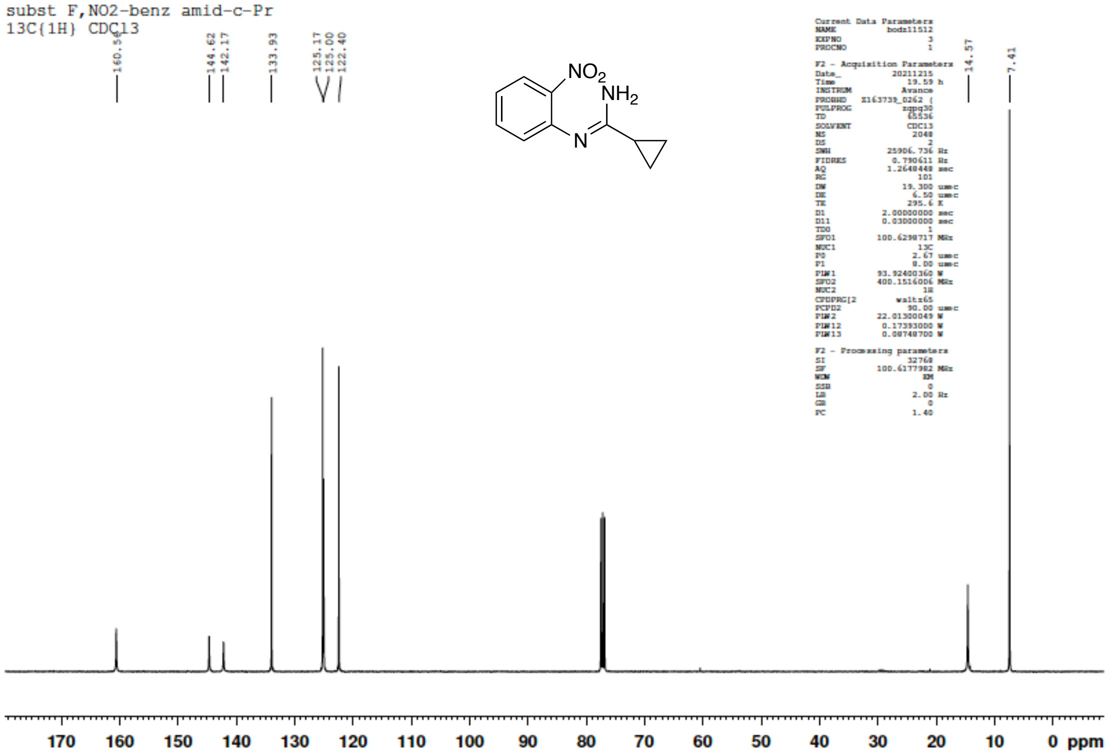

Figure S24. ^{1}H NMR (400 MHz) and $^{13}C\{^{1}H\}$ NMR (100 MHz) spectra of **12m** ($CDCl_3$).

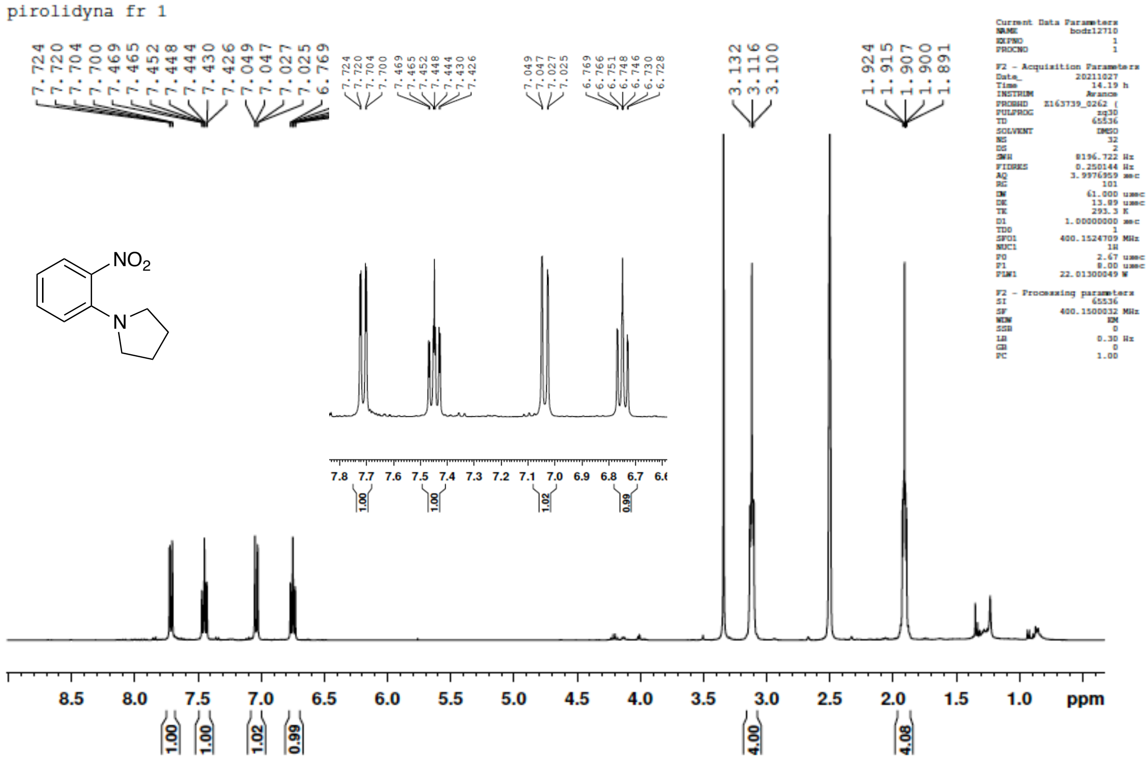

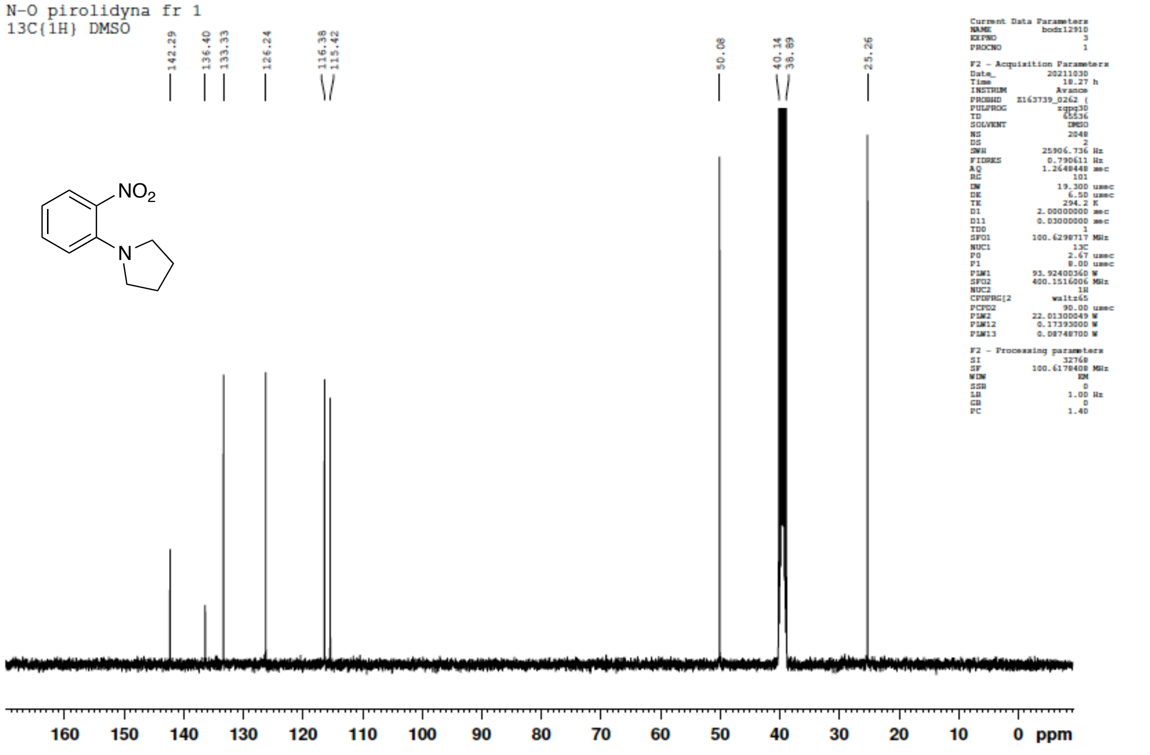

Figure S25. ^{1}H NMR (400 MHz) and ^{13}C{^{1}H} NMR (100 MHz) spectra of **13h** (DMSO-d$_6$).

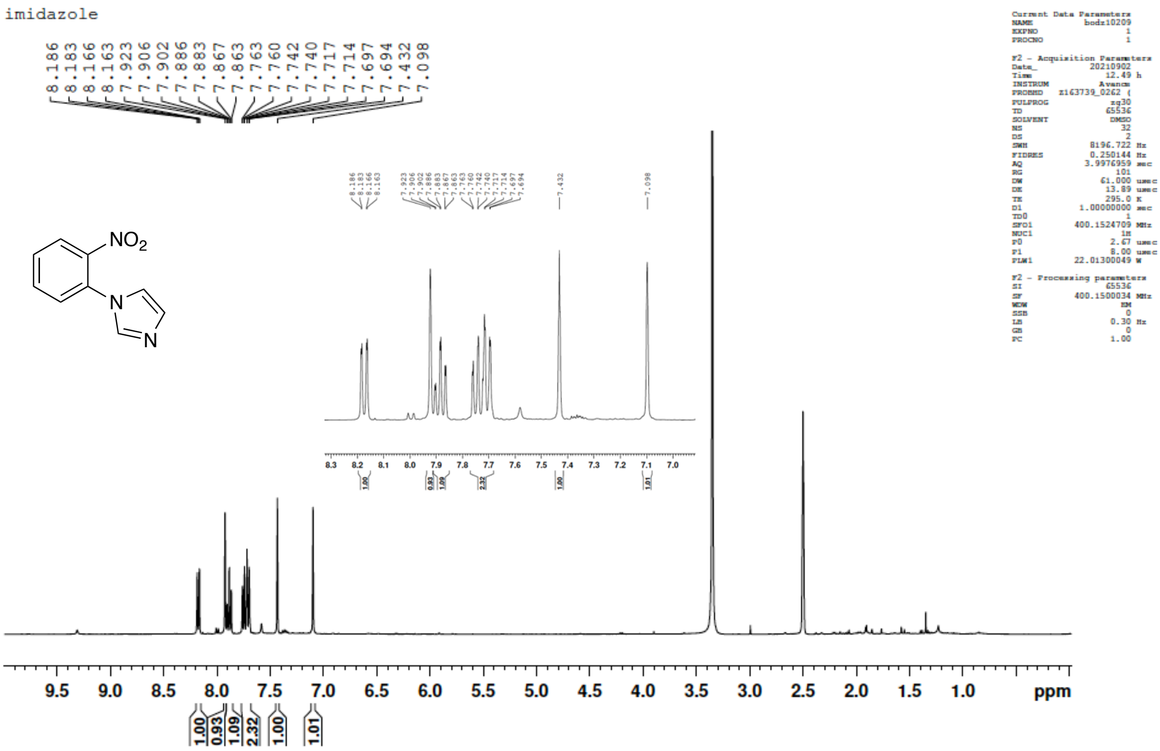

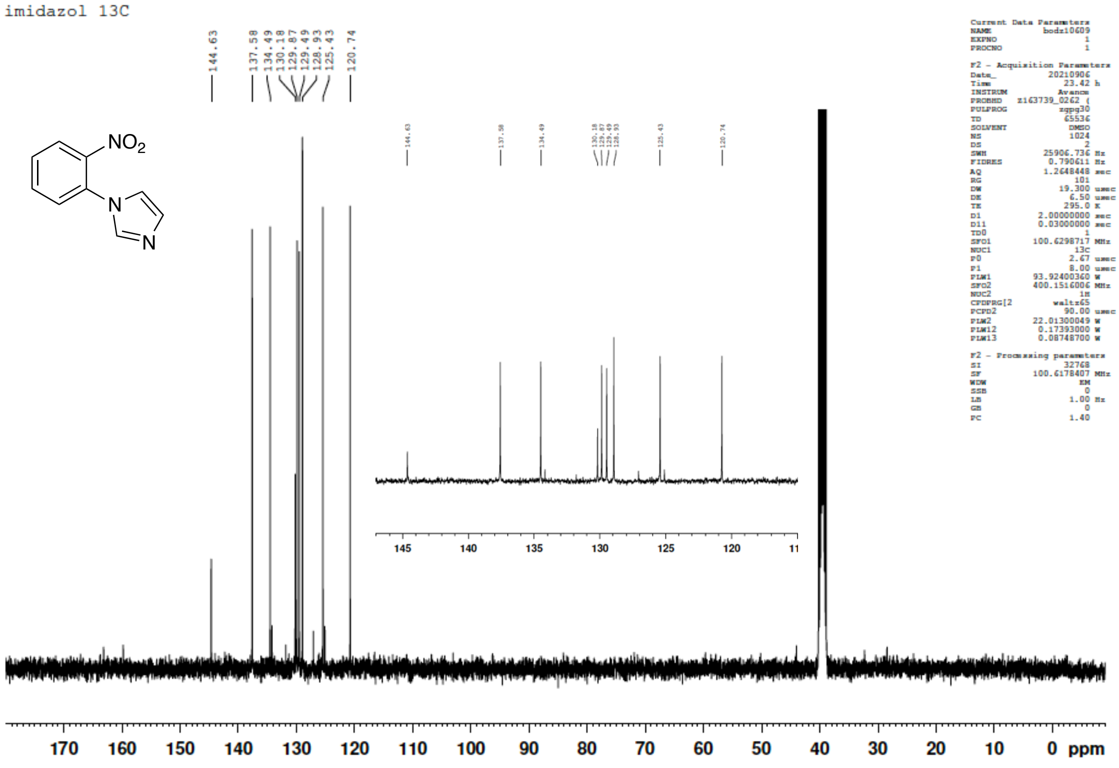

Figure S26. ^{1}H NMR (400 MHz) and $^{13}C\{^{1}H\}$ NMR (100 MHz) spectra of **13j** (DMSO-d_6).

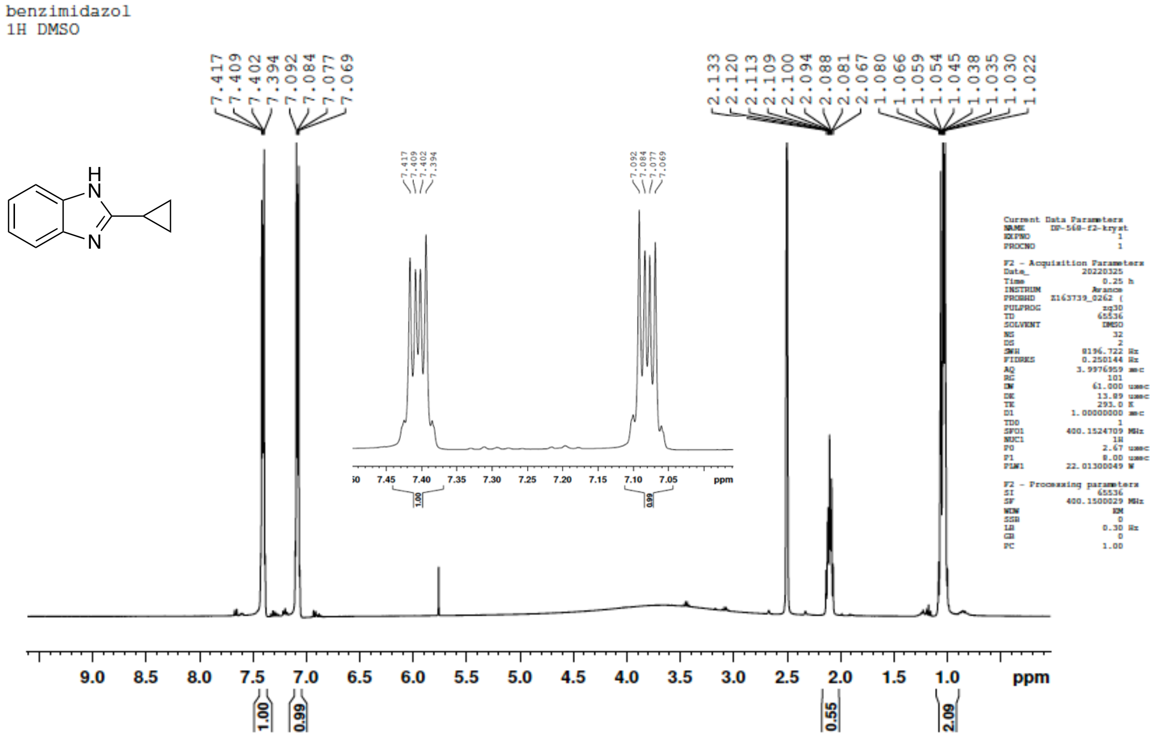

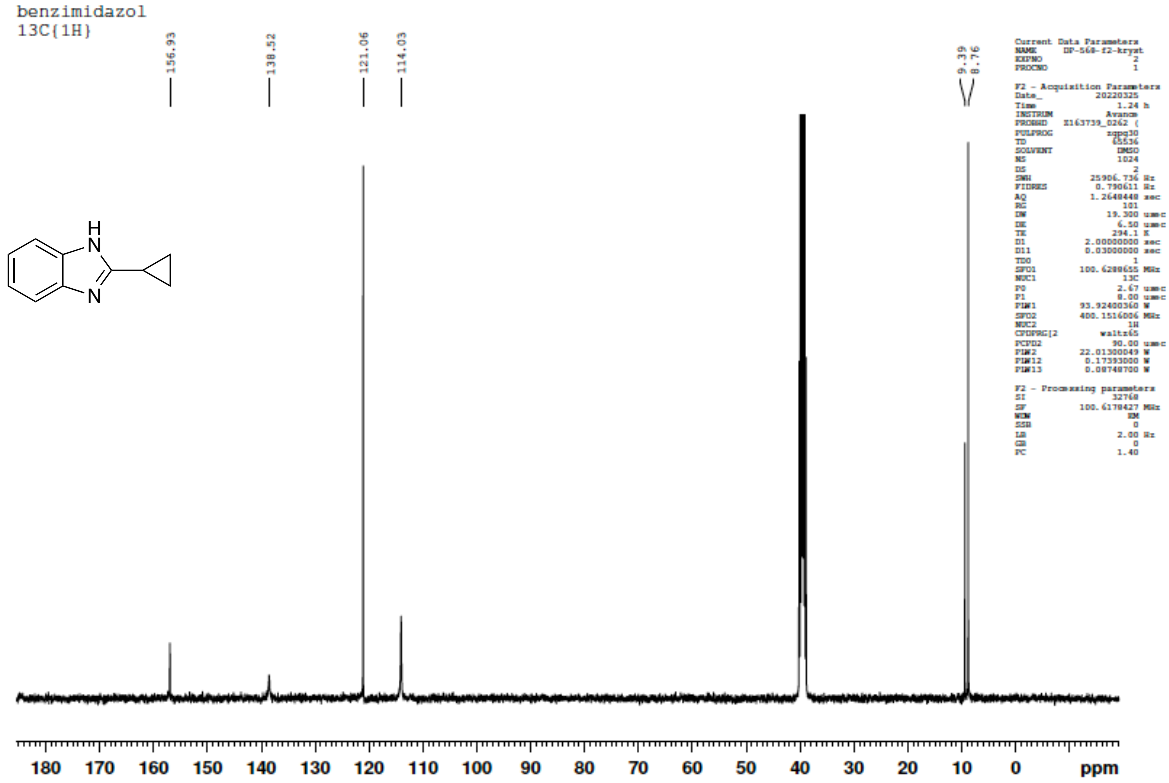

Figure S27. ^{1}H NMR (400 MHz) and ^{13}C{^{1}H} NMR (100 MHz) spectra of **15** (DMSO-d_6).

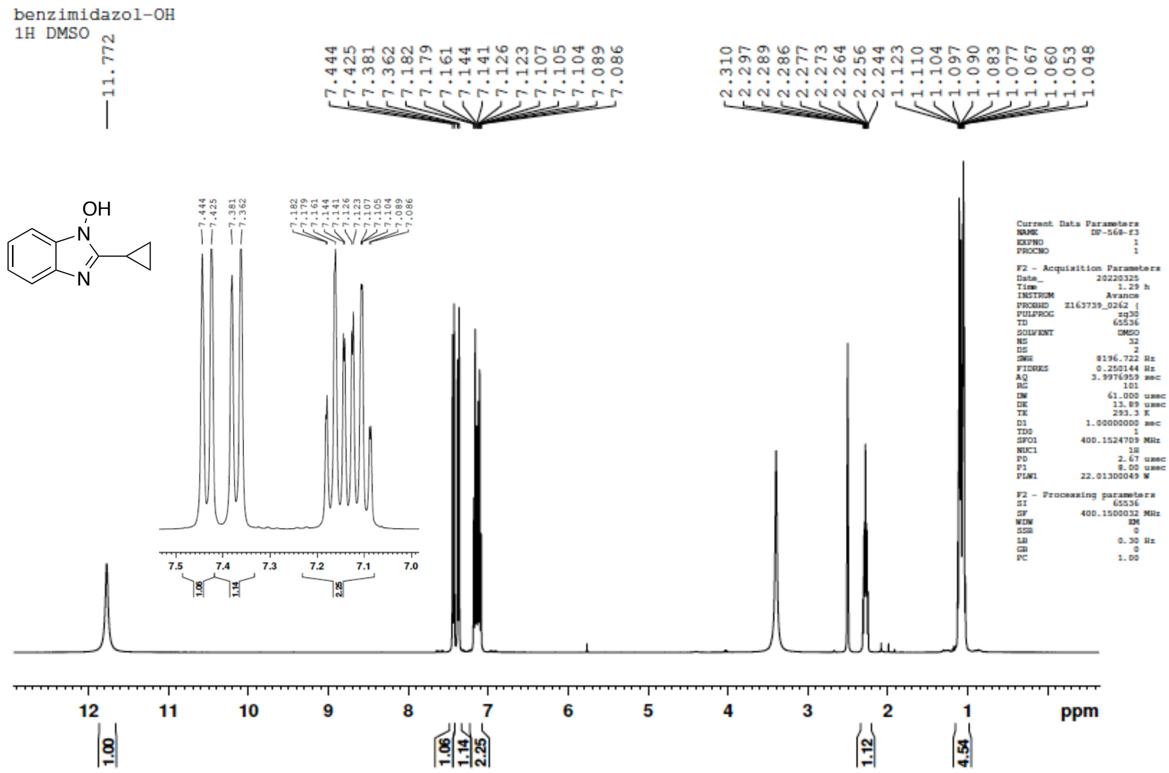

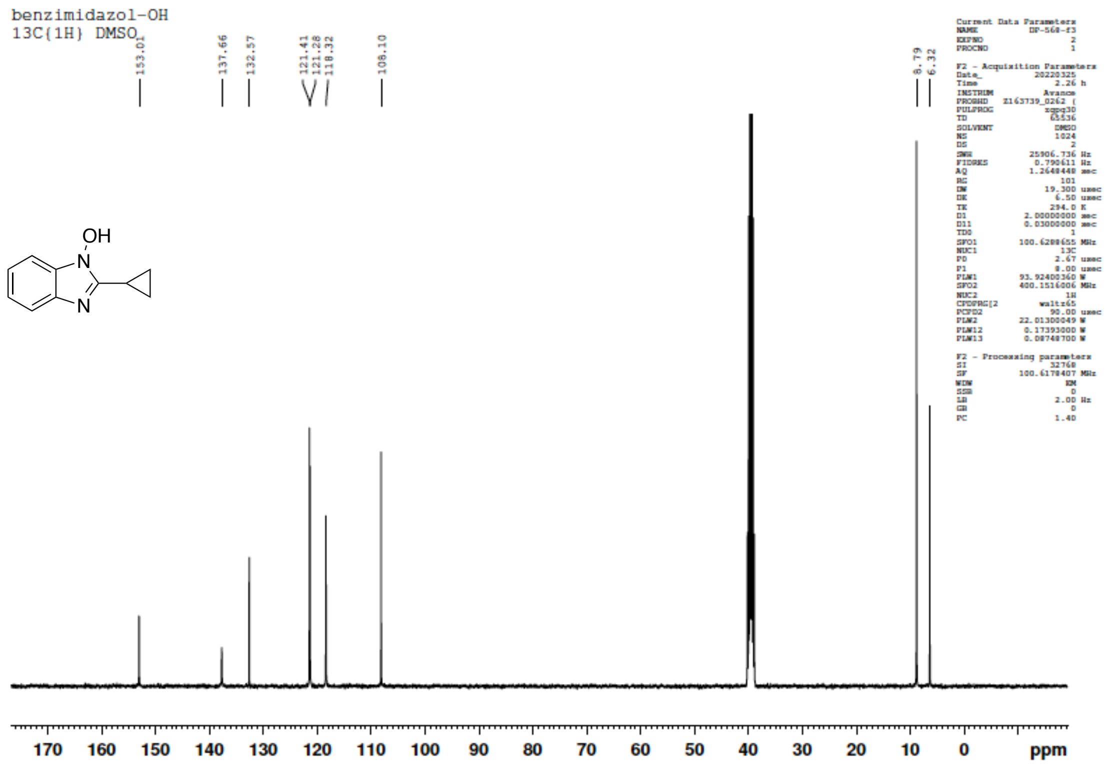

Figure S28. ^{1}H NMR (400 MHz) and $^{13}C\{^{1}H\}$ NMR (100 MHz) spectra of **16** (DMSO-d_6).

2. IR spectra

FT-IR spectra were recorded in KBr pellets.

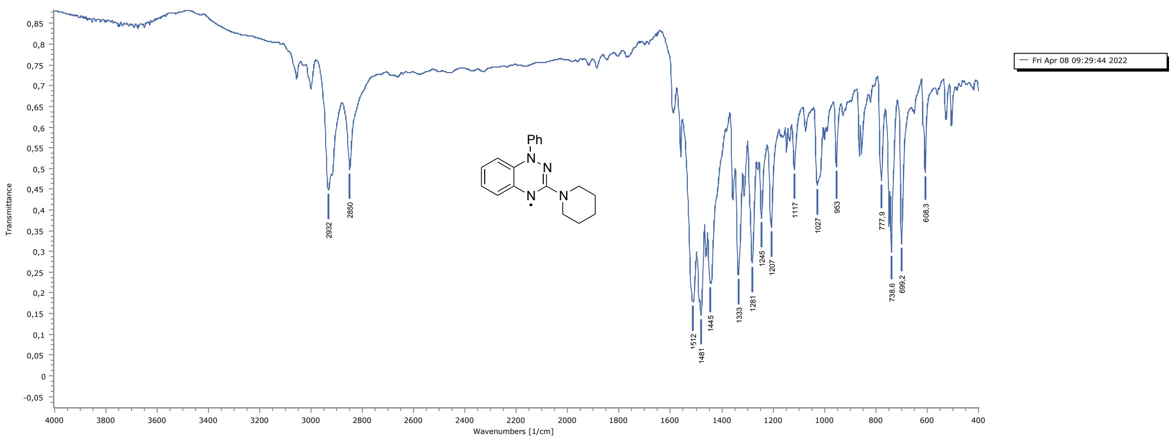

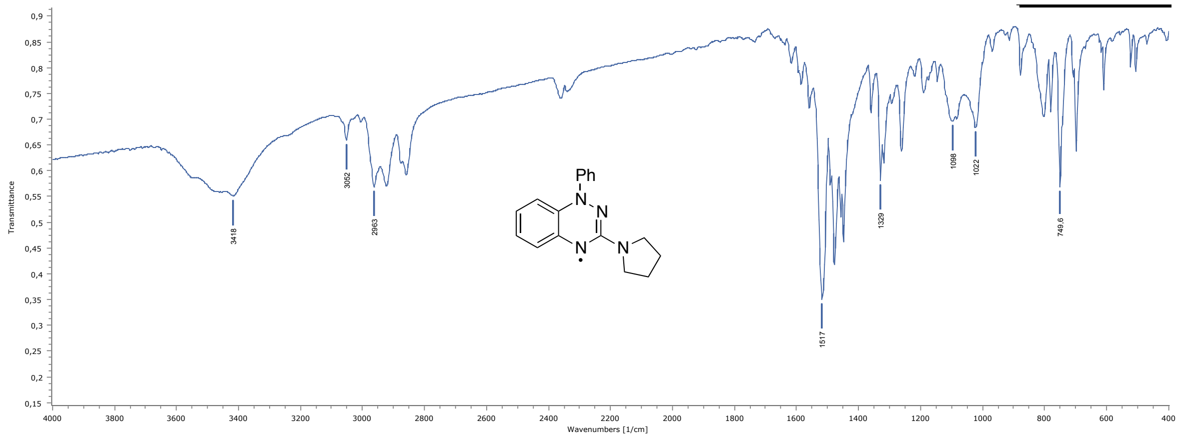

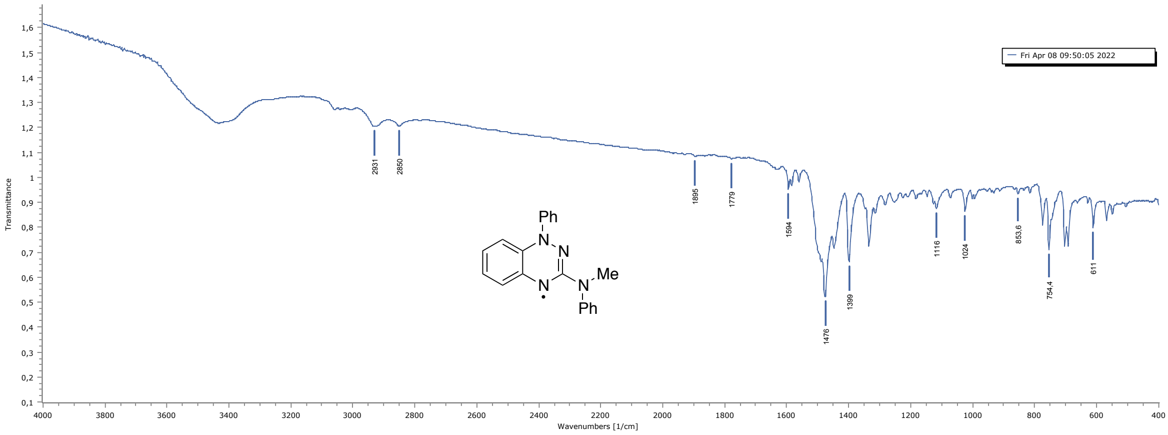

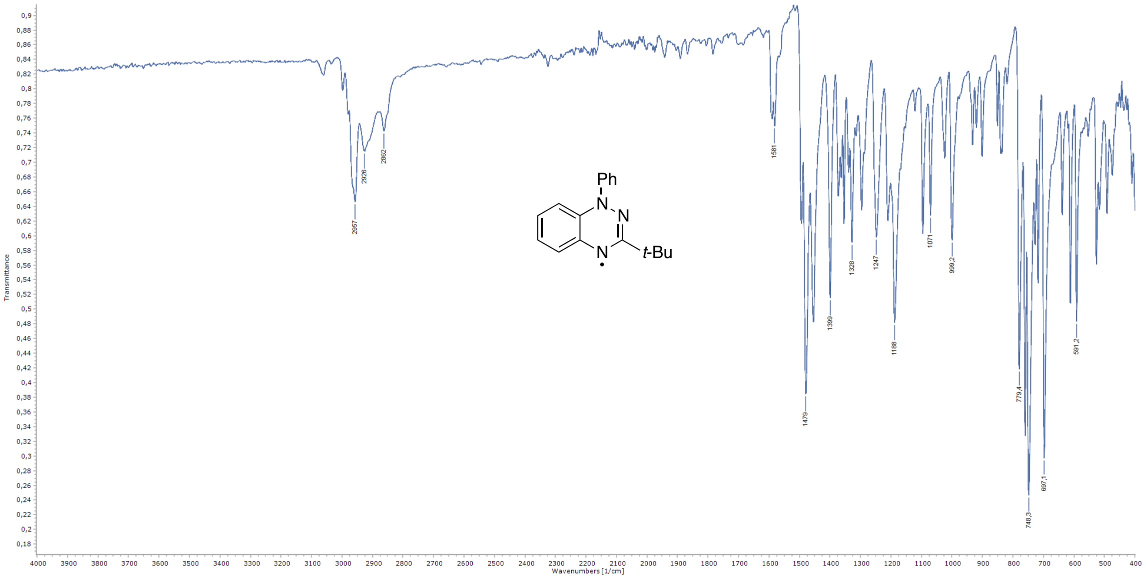

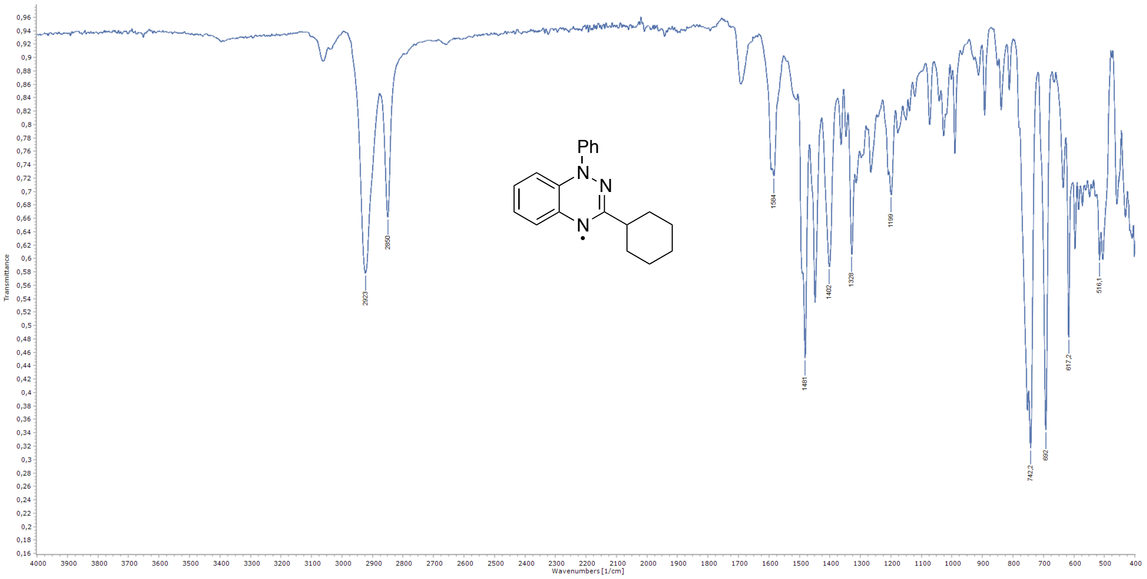

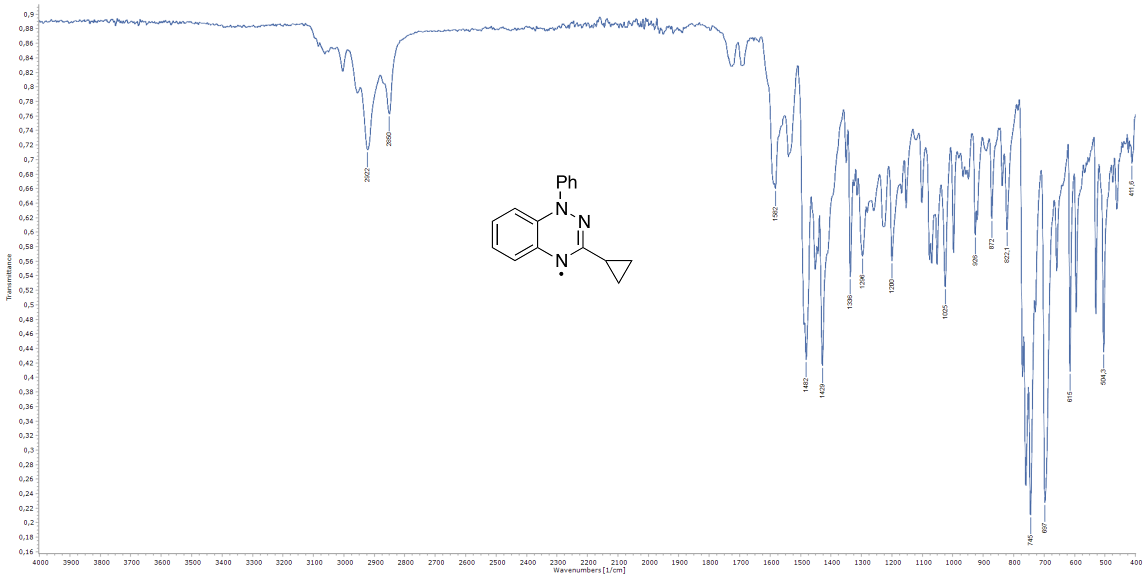

Figure S30. IR spectra for radicals **1** recorded in KBr.

3. UV vis spectroscopy

Electronic absorption spectra for C(3)-substituted radicals **1** were recorded on Jasco V-770 UV-Vis-NIR spectrometer in spectroscopic grade CH_2Cl_2 at concentrations in a range 1.5–10×10^{-5} mol/L and fitted to the Beer–Lambert law.

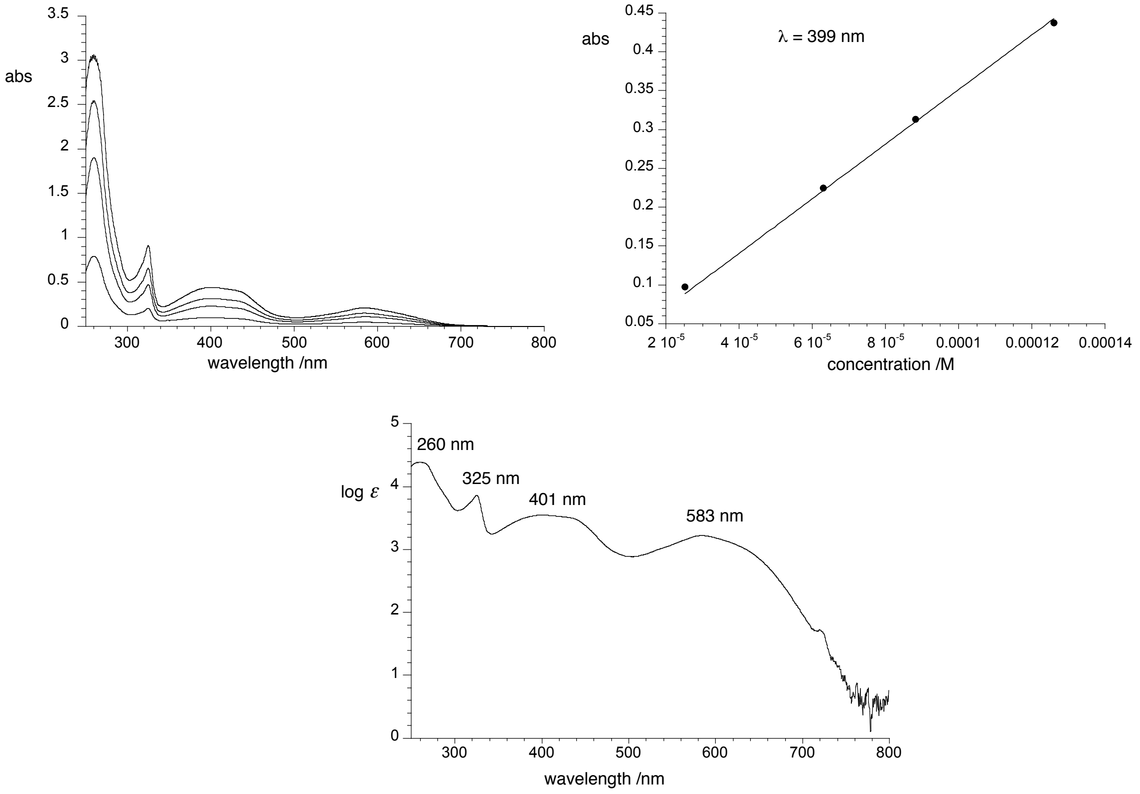

Figure S31. Clockwise: electronic absorption spectra for **1c** (X = morpholin-4-yl) in CH_2Cl_2 for four concentrations (top left), determination of molar extinction coefficient ε at λ = 399 nm (top right, best fit function: ε = 3514×conc, r^2 = 0.9990), and molar extinction log (ε) plot (bottom).[1]

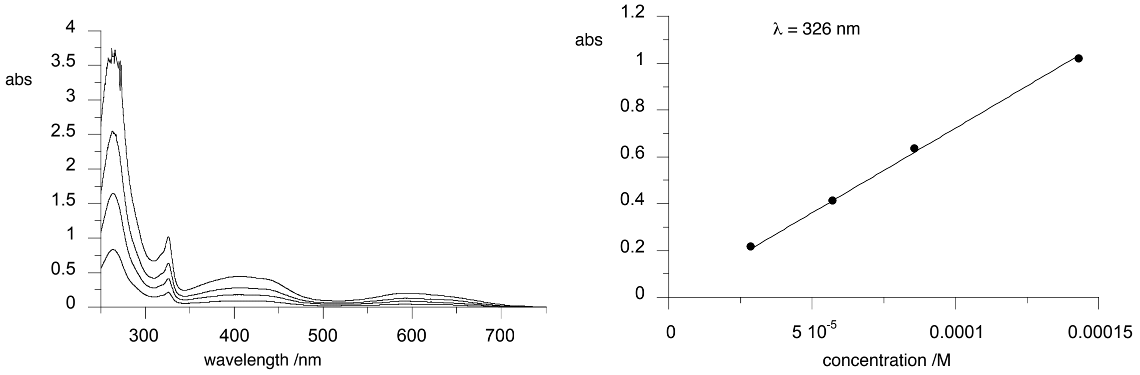

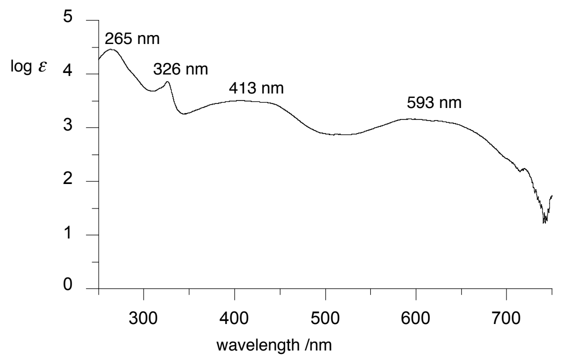

Figure S32. Top: electronic absorption spectra for **1g** (X = piperidin-1-yl) in CH_2Cl_2 for four concentrations (top left), determination of molar extinction coefficient ε at λ = 326 nm (top right, best fit function: ε = 7220×conc, r^2 = 0.9985), molar extinction log (ε) plot (bottom).

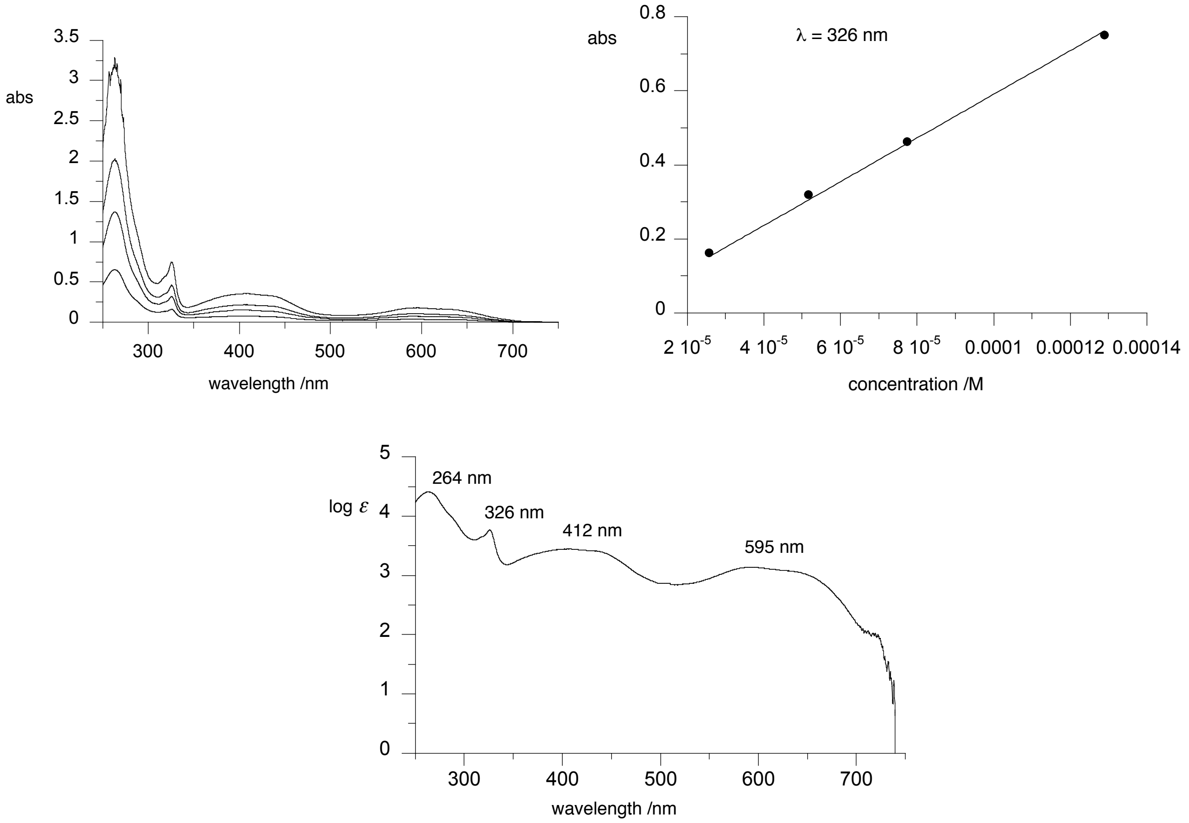

Figure S33. Top: electronic absorption spectra for **1h** (X = pyrrolidin-1-yl) in CH_2Cl_2 for four concentrations (top left), determination of molar extinction coefficient ε at λ = 326 nm (top right, best fit function: ε = 5906×conc, r^2 = 0.9973) molar extinction log (ε) plot (bottom).

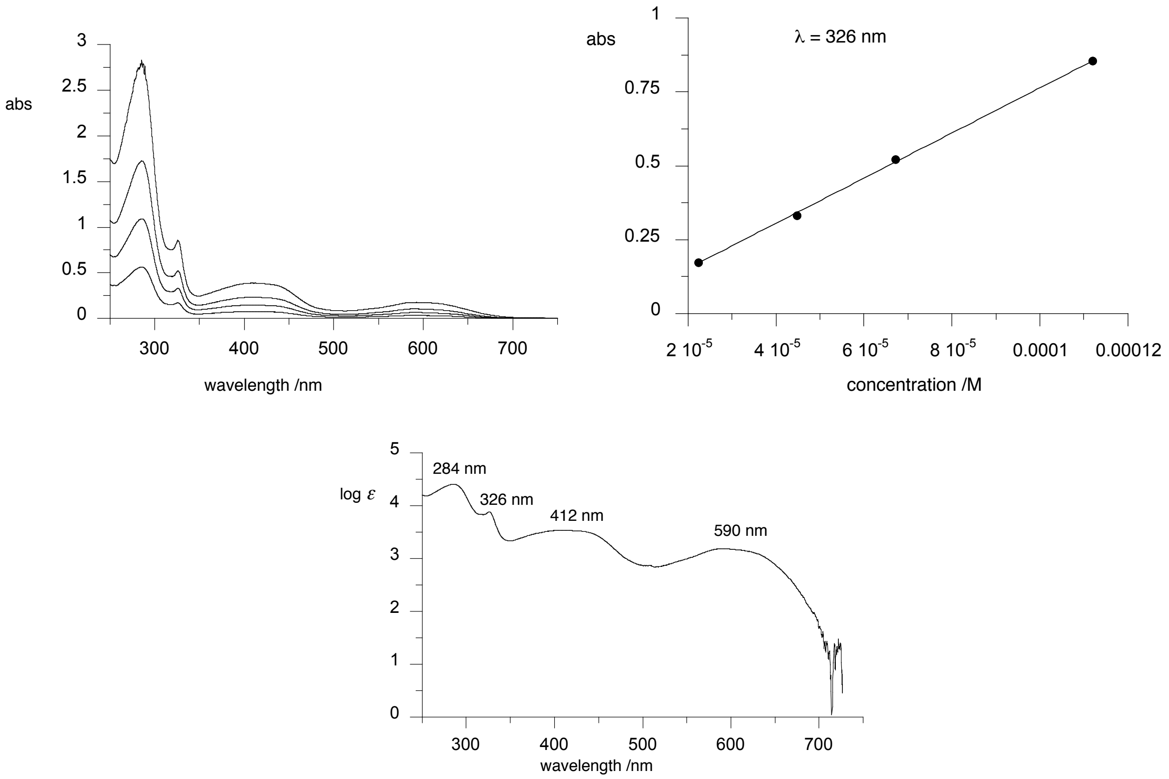

Figure S34. Top: electronic absorption spectra for **1i** (X = NMePh) in CH_2Cl_2 for four concentrations (top left), determination of molar extinction coefficient ε at λ = 326 nm (top right, best fit function: ε = 7638×conc, r^2 = 0.9993), molar extinction log (ε) plot (bottom).

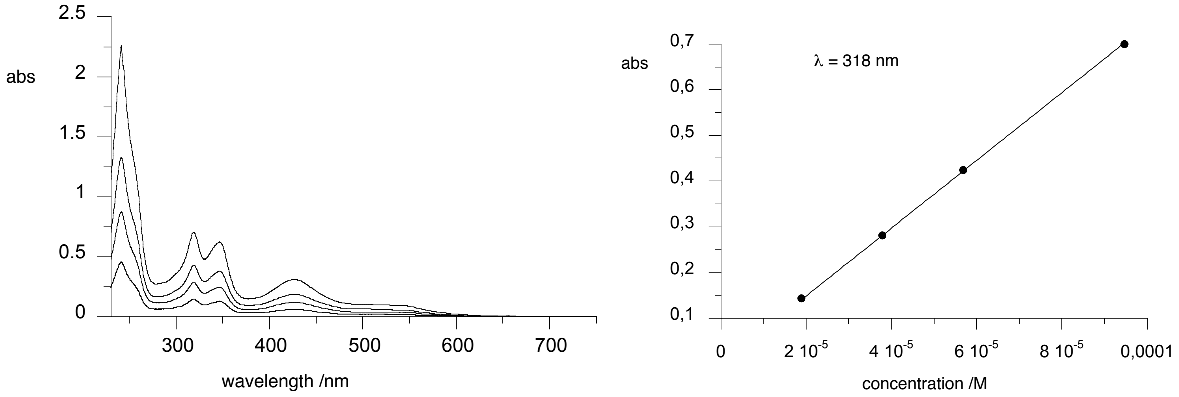

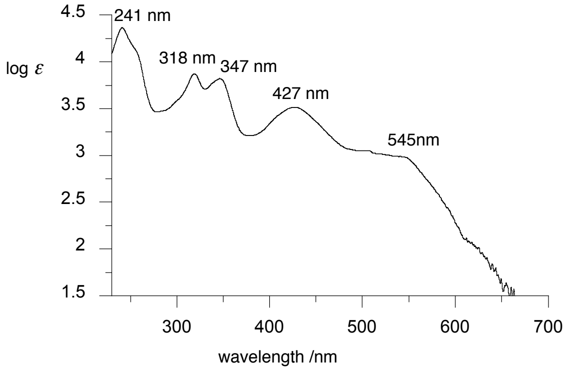

Figure S35. Top: electronic absorption spectra for **1k** (X = *t*-Bu) in CH_2Cl_2 for four concentrations (top left), determination of molar extinction coefficient ε at λ = 318 nm (top right, best fit function: ε = 7423×conc, r^2 = 0.9999), molar extinction log (ε) plot (bottom).

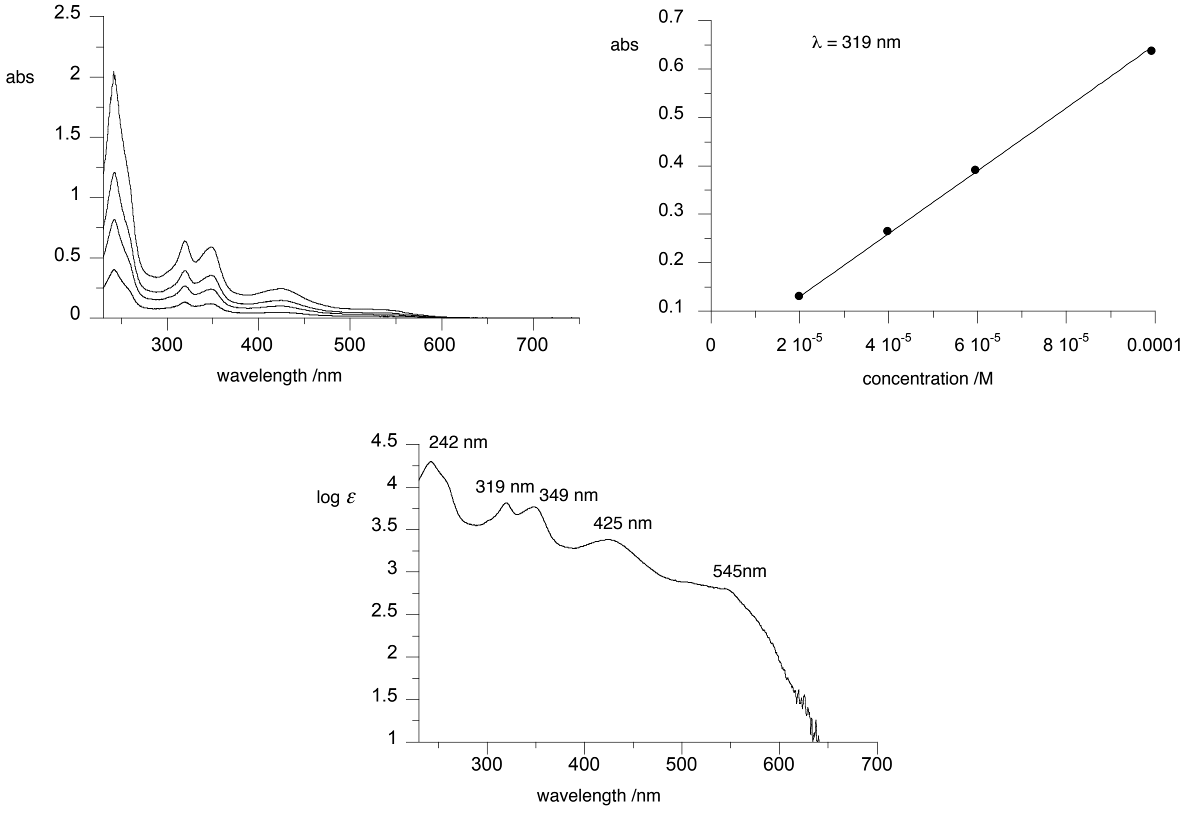

Figure S36. Top: electronic absorption spectra for **1l** (X = *c*-Hex) in CH_2Cl_2 for four concentrations (top left), determination of molar extinction coefficient ε at λ = 319 nm (top right, best fit function: ε = 6505×conc, r^2 = 0.9991), molar extinction log (ε) plot (bottom).

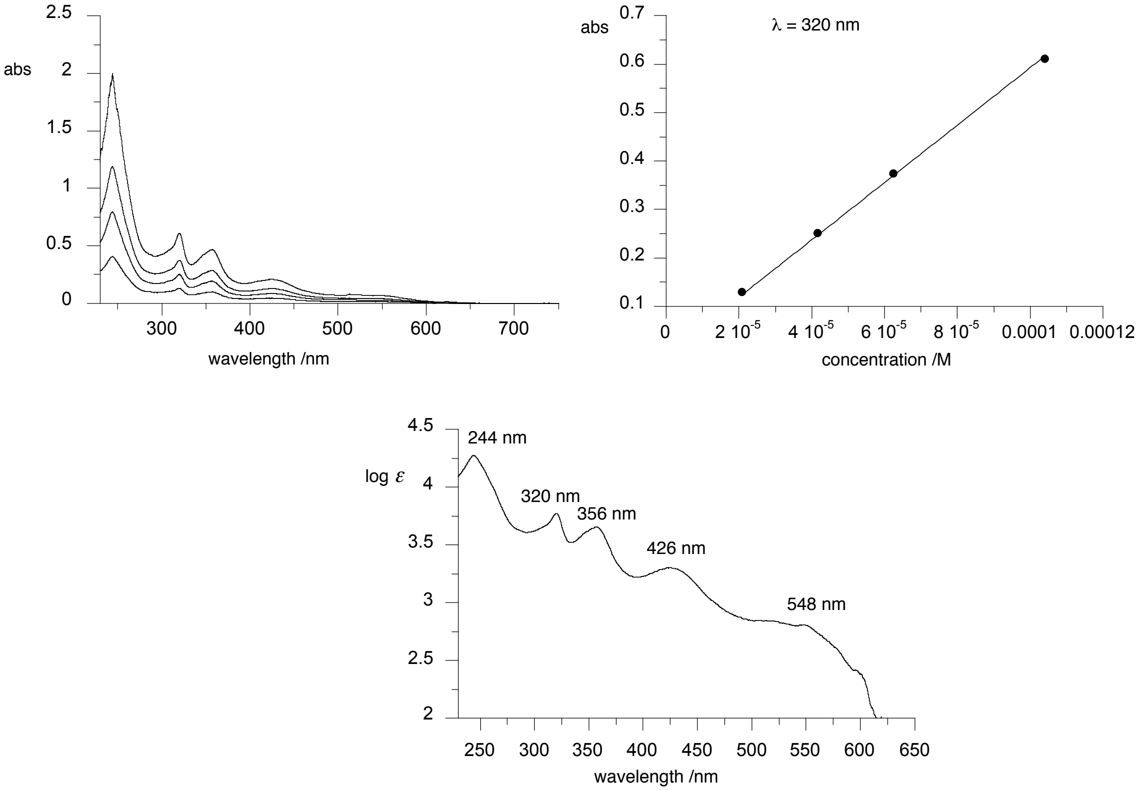

Figure S37. Top: electronic absorption spectra for **1m** (X = *c*-Pr) in CH_2Cl_2 for four concentrations (top left), determination of molar extinction coefficient ε at λ = 320 nm (top right, best fit function: ε = 5925×conc, r^2 = 0.9992), molar extinction log (ε) plot (bottom).

4. Electrochemical results

The electrochemical characterization of selected radicals was conducted using Metrohm Autolab PGSTAT 128N potentiostat/galvanostat instrument. Radical **1** was dissolved in dry, spectroscopic grade CH_2Cl_2 (concentration 0.5 mM) in the presence of $[n\text{-}Bu_4N]^+[PF_6]^-$ as an electrolyte (concentration 50 mM) and the resulting solution was degassed by purging with Ar gas for 10 minutes. A three-electrode electrochemical cell was used with a glassy carbon disk as the working electrode (ϕ 2 mm, alumina polished), Pt wire as the counter electrode and Ag/AgCl wire as the pseudoreference electrode. All samples were measured without internal reference once and afterwards with internal reference Fc/Fc^+ couple (0.46 V *vs* SCE)[2] with a scan rate of 50 mV s^{-1} at *ca.* 20 °C starting from 0.0 V in the oxidative scan direction. CV plots according to IUPAC convention are shown in Figures S38–S44 and numerical result are shown in Table 2 in the main text.

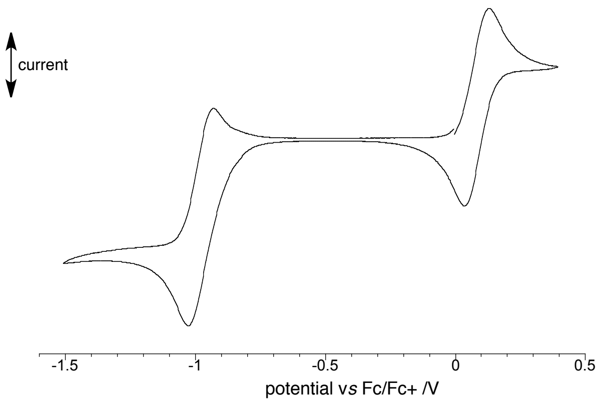

Figure S38. Cyclic voltammogram for radical **1c** (X = morpholin-4-yl).[1] Details are provided above.

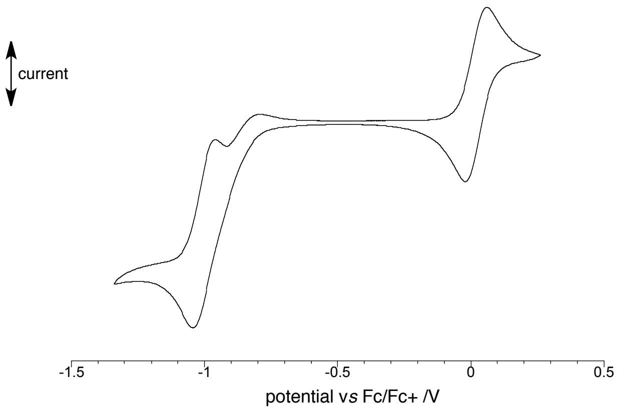

Figure S39. Cyclic voltammogram for radical **1g** (X = piperidin-1-yl). Details are provided above.

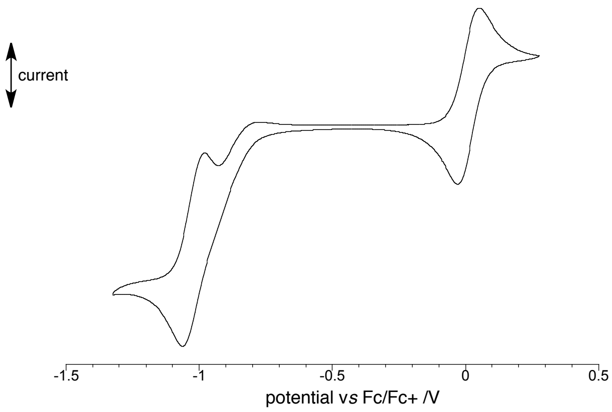

Figure S40. Cyclic voltammogram for radical **1h** (X = pyrrolidin-1-yl). Details are provided above.

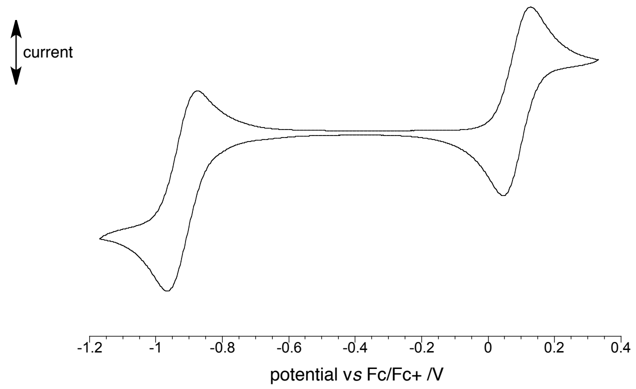

Figure S41. Cyclic voltammogram for radical **1i** (X = NMePh). Details are provided above.

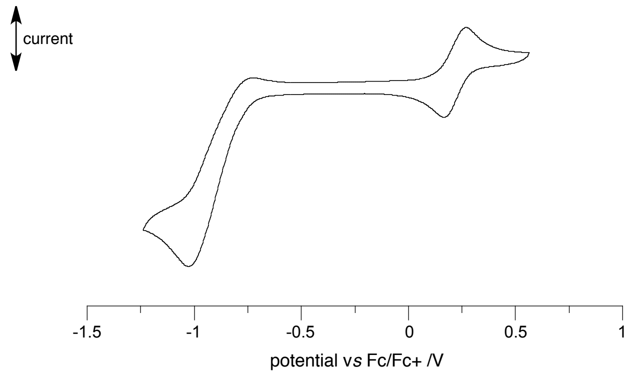

Figure S42. Cyclic voltammogram for radical **1k** (X = *t*-Bu). Details are provided above.

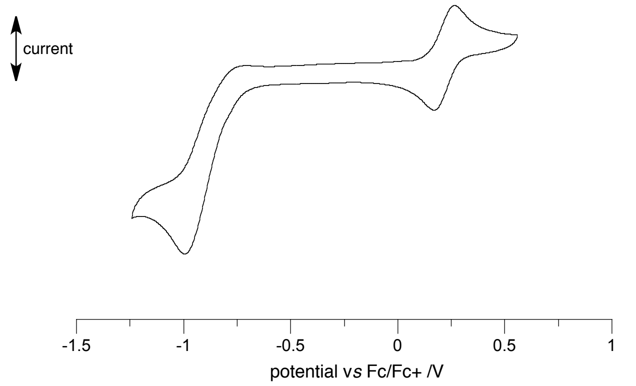

Figure S43. Cyclic voltammogram for radical **1l** (X =*c*-Hex). Details are provided above.

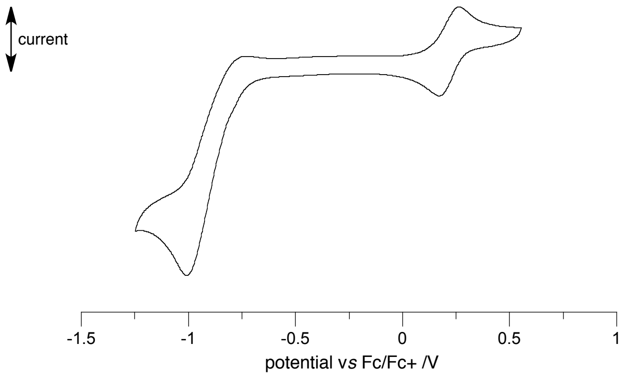

Figure S44. Cyclic voltammogram for radical **1m** (X =*c*-Pr). Details are provided above.

5. EPR spectroscopy

EPR spectra for radicals **1** were recorded on dilute and degassed solutions in benzene at room temperature on an X-band EMX-Nano EPR spectrometer. The microwave power was in a range of 0-5 mW (established with the Power Sweep program below the saturation of the signal) with a modulation frequency of 100 kHz, modulation amplitude of 0.5 G_{pp} and spectral width of 100 G. Accurate g-values were obtained using TEMPO as EMX-Nano internal standard. Simulations of the spectra were performed with the EMX-Nano software using DFT results (*vide infra*) as the starting point including all nitrogen and up to 4 hydrogen atoms. The resulting *hfcc* values were perturbed several times until a global minimum for the fit was achieved. Experimental and simulated spectra are shown in Figures S45–S51 and resulting *hfcc* are listed in Table S1.

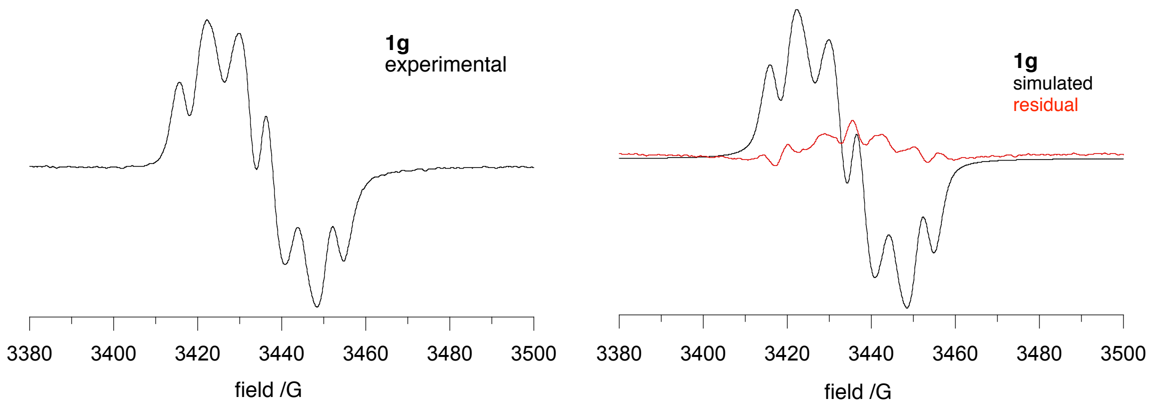

Figure S46. Experimental, simulated and difference spectra for radical **1g** (X = piperidin-1-yl).

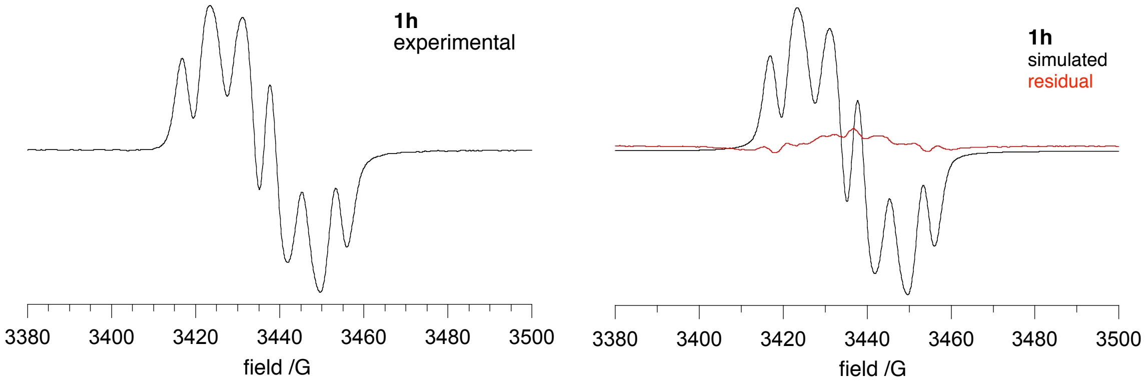

Figure S47. Experimental, simulated and difference spectra for radical **1h** (X = pyrrolidin-1-yl).

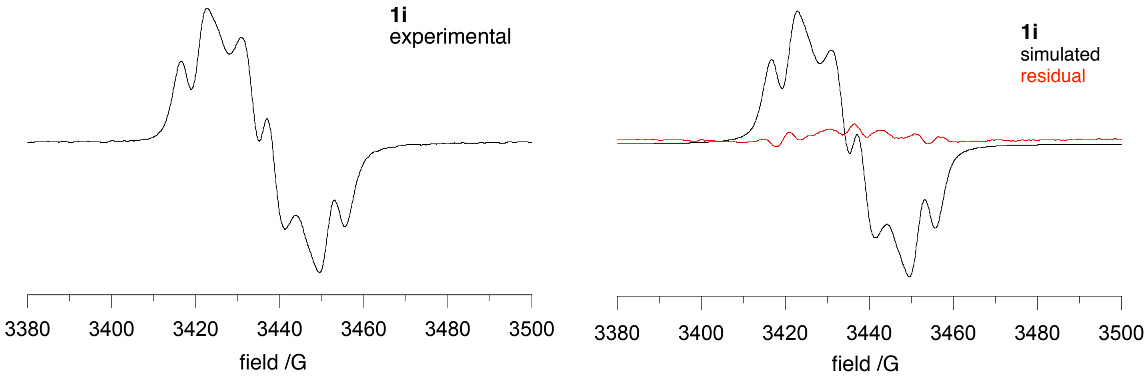

Figure S48. Experimental, simulated and difference spectra for radical **1i** (X= NMePh).

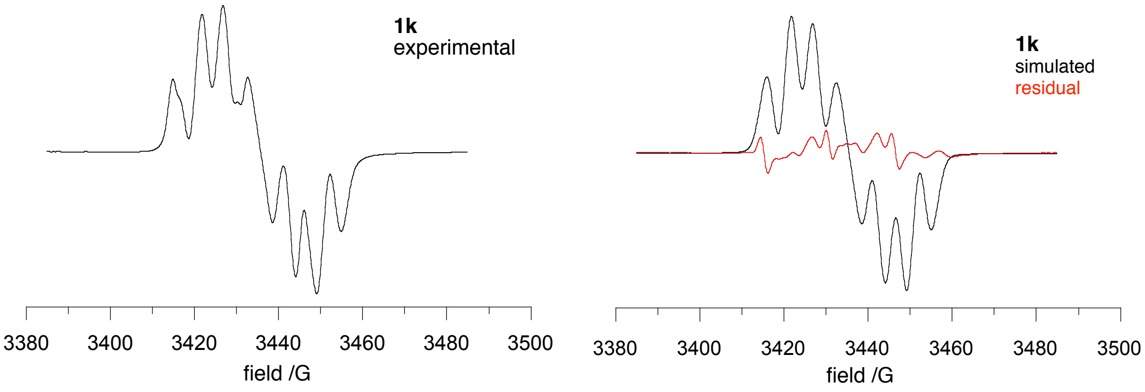

Figure S49. Experimental, simulated and difference spectra for radical **1k** (X= *t*-Bu).

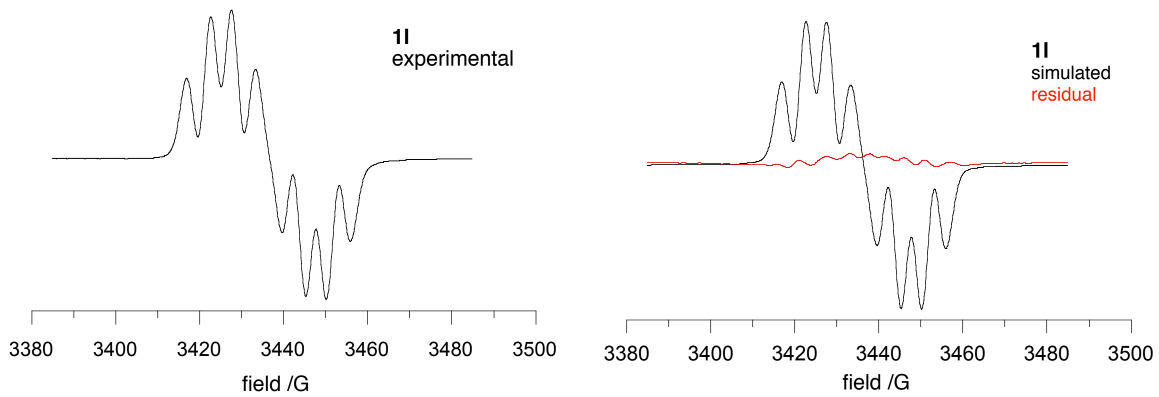

Figure S50. Experimental, simulated and difference spectra for radical **1l** (X= *c*-Hex).

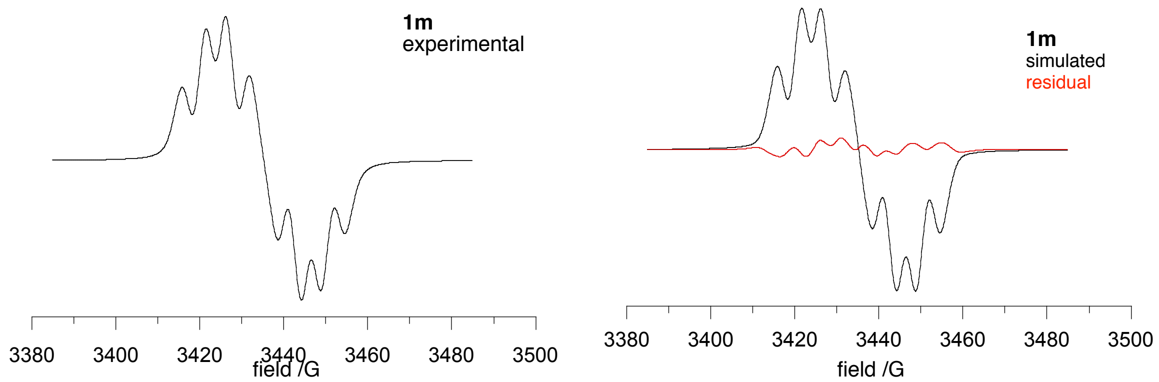

Figure S51. Experimental, simulated and difference spectra for radical **1m** (X= *c*-Pr).

Table S1. Summary of hyperfine coupling constants (G) for radicals **1**.[a]

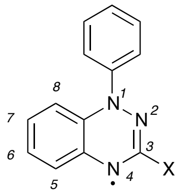

Compound	a_{N1}[a]	a_{N2}[a]	a_{N4}[a]	a_H	a_H	a_H	a_H	a_x	g[b]
**1a,** Ph[c]	7.65	4.87	4.90	1.32	1.58	1.03	0.58	-	2.0033
**1b,** NH_2[d]	7.96	4.24	5.71	1.55	0.98	0.45	0.36	*N :* 0.92	2.0035
**1c,** morpholi-4-yl[d]	7.99	4.13	5.75	1.67	0.62	1.00	0.02	*N*: 0.92	2.0033
**1g**, piperydyn-1-yl	7.79	4.09	5.88	1.25	1.25	0.92	1.25	*N*: 0.85	2.0037
**1h**, pyrrolidin-1-yl	7.83	4.07	5.93	1.12	1.11	1.12	1.12	*N*: 0.80	2.0036
**1i**, NMePh	7.78	5.34	5.34	1.25	1.25	0.93	1.25	*N*: 0.81	2.0036
**1k**, *t*-Bu	7.46	4.82	5.34	-	-	-	-	-	2.0047
**1l,** *c*-Hex	7.54	5.02	5.02	1.20	1.20	1.20	0.20	-	2.0044
**1m**, *c*-Pr	7.52	4.96	4.96	-	-	-	-	-	2.0039

[a] Assignments follow the previous EPR and ENDOR studies on ^{15}N-labeled derivatives (Ref. [3]), [b] Referenced to TEMPO as the internal standard. [c] Ref. [4]. [d] Ref.[1]

6. Computational details and results

a) geometry optimization

Quantum-mechanical calculations were carried out using Gaussian 09 suite of programs.[5] Geometry optimizations were undertaken at the B3LYP/6-31G(2d,p) level of theory for radicals **1**. Vibrational frequencies were used to characterize the nature of stationary points and to obtain thermodynamic parameters. Zero-point energy (ZPE) correction were scaled by 0.9806.

b) hfcc calculations

Isotropic Fermi contact coupling constants for radicals **1** were calculated using the CAM-B3LYP/EPR-III // UB3LYP/6-31G(2d,p) method in benzene dielectric medium requested with ythe SCRF(Solvent=Benzene) keyword (PCM model).

Table S2. Calculated hyperfine coupling constants (G) for radicals **1**.[a]

Radical **1**	$\alpha_{N(1)}$	$\alpha_{N(2)}$	$\alpha_{N(4)}$	$\alpha_{H(5)}$	$\alpha_{H(6)}$	$\alpha_{H(7)}$	$\alpha_{H(8)}$	$\alpha_{H(o)}$	$\alpha_{H(m)}$	$\alpha_{H(p)}$
**1a**, Ph	5.86	4.37	4.65	-1.73	-0.62	-2.1	-0.41	-1.12	0.82	-0.99
**1b,** NH_2	6.27	4.88	3.94	-1.05	-1.12	-1.35	-0.92	-1.25	0.90	-1.14
**1c**, morpholin-4-yl	6.28	5.00	3.78	-0.96	-1.21	-1.23	-1.03	-1.19	0.88	-1.06
**1g**, piperidinyl	6.23	5.12	3.69	-0.96	-1.20	-1.18	-1.06	-1.22	0.89	-1.09
**1h**, pyrrolidinyl	6.20	5.11	3.72	-1.01	-1.13	-1.21	-1.01	-1.25	0.89	-1.14
**1i**, NMePh	6.23	5.06	3.75	-0.98	-1.16	-1.22	-1.00	-1.26	0.90	-1.16
**1k**, *t*-Bu	5.74	4.08	4.90	-1.92	-0.44	-2.33	-0.26	-1.17	0.80	-1.04
**1l**, *c*-Hex	5.81	4.26	4.76	-1.79	-0.56	-2.17	-0.35	-1.21	0.82	-1.08
**1m**, *c*-Pr	5.89	4.32	4.67	-1.68	-0.65	-2.06	-0.43	-1.21	0.83	-1.07

[a] *hfcc* in gauss (G) calculated with the CAM-B3LYP/EPR-III // UB3LYP/6-31G(2d,p) method in benzene dielectric medium.

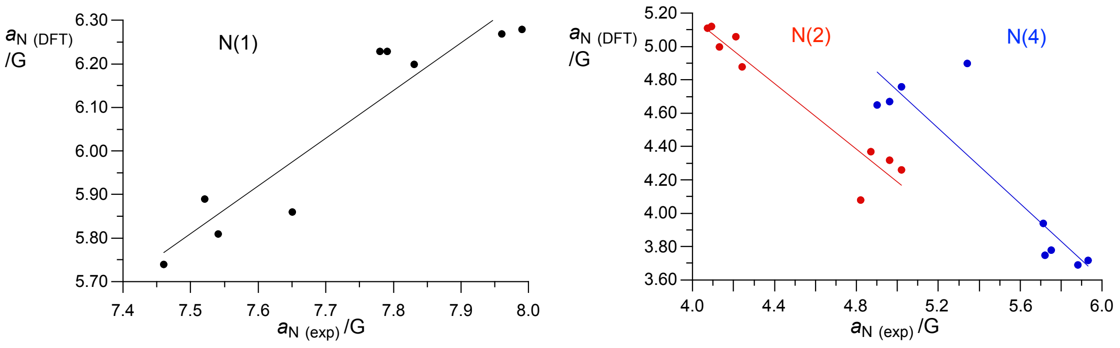

Figure S52. A comparison of experimental and DFT-derived *hfcc* for the ring nitrogen atoms in **1**.

c) Electronic absorption data

Electronic excitation energies in CH_2Cl_2 dielectric medium were obtained for derivatives **1** at the CAM-B3LYP/6-31G(2d,p) // UB3LYP/6-31G(2d,p) level of theory using time-dependent DFT method supplied in the Gaussian 09 package. Solvation models in calculations were implemented by PCM model using the SCRF(solvent=CH2CL2) keyword. Three lowest excitation energies, classified as $\pi \rightarrow \pi^*$ transitions, are listed in Table S3.

Energies of FMOs involved in the low energy transitions are listed in Table S4.

Table S3. Calculated electronic transition energies and oscillator strength values.[a]

Radical **1**	$\pi \rightarrow \pi^*$ /nm (*f*)	$\pi \rightarrow \pi^*$ nm (*f*)	$\pi \rightarrow \pi^*$ nm (*f*)
**1a**, Ph	515.9 (0.005)[b]	445.0 (0.050)[c]	387.7 (0.002)
**1b,** NH_2	478.2 (0.068)[c]	416.5 (0.001)[b]	371.3 (0.038)
**1c,** morpholin-4-yl	513.5 (0.057)[c]	415.9 (0.005)[b]	385.4 (0.039)
**1g,** piperidinyl	530.5 (0.057)[c]	415.0 (0.009)[b]	390.8 (0.030)
**1h,** pyrrolidinyl	534.1 (0.059)[c]	414.7 (0.010)[b]	388.5 (0.048)
**1i,** NMePh	513.8 (0.058)[c]	420.9 (0.004)[b]	402.6 (0.037)
**1k**, tBu	462.2 (0.034)[b]	432.7 (0.025)[c]	393.1 (0.003)
**1l**, *c*-Hex	462.3 (0.033)[b]	432.0 (0.027)[c]	385.3 (0.003)
**1m**, *c*-Pr	465.8 (0.038)[b]	433.2 (0.018)[b]	380.2 (0.002)

[a] Obtained with the TD CAM-B3LYP/6-31G(2d,p) // UB3LYP/6-31G(2d,p) method in CH_2Cl_2 dielectric medium. [b] Main component α-HOMO/α-LUMO. [c] Main component β-HOMO – β-LUMO.

Table S4. Calculated energies for selected MOs.[a]

Radical **1**	α-HOMO /eV	α-LUMO /eV	β-HOMO /eV	β-LUMO /eV
**1a,** Ph	-6.24	-0.47	-7.65	-1.69
**1b**, NH_2	-6.20	0.21	-7.50	-1.61
**1c**, morpholin-4-yl	-6.13	0.23	-7.09	-1.53
**1g**, Piperidinyl	-6.06	0.27	-6.93	-1.47
**1h**, pyrrolidinyl	-6.05	0.27	-6.91	-1.46
**1i**, NMePh	-6.13	0.23	-6.96	-1.54
**1k**, *t*Bu	-6.17	0.19	-7.81	-1.62
**1l**, *c*-Hex	-6.19	0.17	-7.82	-1.64
**1m**, *c*-Pr	-6.20	0.17	-7.75	-1.65

[a] Obtained with the TD CAM-B3LYP/6-31G(2d,p) // UB3LYP/6-31G(2d,p) method in CH_2Cl_2 dielectric medium.

d) oxidation potentials for radicals 1

The oxidation potential $E_{1/2}^{0/+1}$ for radicals **1** was calculated at the (U)B3LYP/6-31++G(2d,p) // (U)B3LYP/6-31G(2d,p) level of theory in CH_2Cl_2 dielectric medium (single point calculations) using the following process and equation S1:

$$\mathbf{1} \rightarrow \mathbf{1}^{+} \qquad E_{1/2}^{0/+1} = \Delta G_{298}/23.016 - (4.44V + 0.71V) \qquad \text{eq S1}$$

where ΔG_{298} is the free energy change in kcal mol^{-1}, 23.016 in conversion of kcal mol^{-1} to eV, 4.44±0.02 V is the absolute potential of standard H^+/H electrode, (SHE),[6] and 0.71 V is the relative standard potential of the Fc/Fc^+ electrode (*vs* SHE).

Thermodynamic corrections were obtained at the (U)B3LYP/6-31G(2d,p) level of theory in vacuum. The results are shown in Table S5.

Table S5. Calculated oxidation potentials for radicals **1**.[a]

Radical **1**	$E_{1/2}^{0/+1}$
**1a,** Ph	-0.25
**1b,** NH_2	-0.46
**1c,** morpholin-4-yl	-0.51
**1g,** piperidinyl	-0.58
**1h,** pyrrolidinyl	-0.59
**1i,** NMePh	-0.63
**1k,** *t*-Bu	-0.39
**1l,** cyclohexyl	-0.38
**1m,** cyclopropyl	-0.39

[a] Obtained with the (U)B3LYP/6-31++G(2d,p) // (U)B3LYP/6-31G(2d,p) method in CH_2Cl_2 dielectric medium using eq S1.

e) partial output data for TD-DFT calculations

Excitation energies were calculated with the TD CAM-B3LYP/6-31++G(2d,p)//B3LYP/6-31G(2d,p) method. Only three lowest energy excitations are listed along with energies of relevant MOs.

```
1a, X=Ph
Excited State   1:  2.173-A      2.4031 eV  515.94 nm  f=0.0047  <S**2>=0.930
     75A -> 76A        0.85485
     75A -> 86A        0.14803
     75A -> 87A       -0.13617
     74B -> 75B        0.33368
 This state for optimization and/or second-order correction.
 Total Energy, E(TD-HF/TD-KS) =  -896.162275001
 Copying the excited state density for this state as the 1-particle RhoCI
density.

 Excited State   2:  2.353-A      2.7865 eV  444.95 nm  f=0.0401  <S**2>=1.134
     74A -> 76A        0.13747
     75A -> 76A       -0.31943
     75A -> 77A       -0.12494
     69B -> 75B       -0.10676
     73B -> 75B        0.28321
     73B -> 76B        0.13916
     74B -> 75B        0.78279
     74B -> 76B       -0.11603

 Excited State   3:  2.247-A      3.1980 eV  387.69 nm  f=0.0022  <S**2>=1.012
     74A -> 76A        0.11782
     69B -> 75B        0.73598
     70B -> 75B        0.27794
     71B -> 75B       -0.43966
     73B -> 75B       -0.12708
     74B -> 76B       -0.12545
Alpha  occ. eigenvalues --   -0.40984  -0.39653  -0.36319  -0.33875  -0.33101
Alpha  occ. eigenvalues --   -0.32353  -0.31955  -0.31384  -0.29553  -0.22944
Alpha virt. eigenvalues --   -0.01719   0.00737   0.01318   0.01876   0.02195
  Beta  occ. eigenvalues --   -0.40422  -0.38912  -0.34306  -0.33171  -0.32672
  Beta  occ. eigenvalues --   -0.32088  -0.31510  -0.30794  -0.28106
  Beta virt. eigenvalues --   -0.06225  -0.01325   0.01142   0.01360   0.01988
```

```
1b, X=NH2
Excited State   1:  2.268-A      2.5927 eV  478.21 nm  f=0.0679  <S**2>=1.036
     59A -> 62A       -0.13505
     59A -> 63A       -0.11684
     58B -> 59B        0.91343
 This state for optimization and/or second-order correction.
 Total Energy, E(TD-HF/TD-KS) =  -720.569364398
 Copying the excited state density for this state as the 1-particle RhoCI
density.

 Excited State   2:  2.300-A      2.9768 eV  416.50 nm  f=0.0010  <S**2>=1.072
     59A -> 60A        0.63893
     59A -> 61A        0.25292
     59A -> 62A       -0.41313
     59A -> 63A       -0.31481
     59A -> 65A       -0.11615
     59A -> 67A        0.12664
     57B -> 59B        0.12558
     57B -> 60B        0.11432
     58B -> 59B       -0.25520
```

```
 Excited State   3:  2.594-A      3.3390 eV  371.33 nm  f=0.0383  <S**2>=1.432
     56A -> 60A        0.19956
     57A -> 61A        0.10262
     57A -> 63A        0.16088
     59A -> 60A        0.15337
     59A -> 61A       -0.12840
     59A -> 62A        0.20386
     59A -> 63A        0.20600
     54B -> 59B        0.37298
     55B -> 60B        0.17588
     56B -> 59B        0.22162
     56B -> 63B       -0.15442
     57B -> 59B        0.55155
     58B -> 59B        0.13667
     58B -> 60B       -0.13773
Alpha  occ. eigenvalues --   -0.41997  -0.40469  -0.37434  -0.34147  -0.33385
Alpha  occ. eigenvalues --   -0.32825  -0.32313  -0.29338  -0.22777
Alpha virt. eigenvalues --    0.00769   0.01275   0.01827   0.02176   0.02413
  Beta  occ. eigenvalues --   -0.41127  -0.39656  -0.36153  -0.33439  -0.32566
  Beta  occ. eigenvalues --   -0.32058  -0.30970  -0.27561
  Beta virt. eigenvalues --   -0.05904   0.01110   0.01329   0.01970   0.02318
```

1c, X=morpholin-4-yl

```
Excited State   1:  2.170-A      2.4144 eV  513.52 nm  f=0.0570  <S**2>=0.927
     78A -> 81A       -0.11412
     77B -> 78B        0.94002
 This state for optimization and/or second-order correction.
 Total Energy, E(TD-HF/TD-KS) =  -951.723269352
 Copying the excited state density for this state as the 1-particle RhoCI
density.

 Excited State   2:  2.352-A      2.9815 eV  415.85 nm  f=0.0049  <S**2>=1.133
     78A -> 79A        0.61441
     78A -> 80A        0.17027
     78A -> 81A        0.42971
     78A -> 82A        0.19777
     78A -> 84A        0.31245
     78A -> 85A       -0.21287
     78A -> 86A       -0.11361
     76B -> 79B       -0.10445
     77B -> 78B        0.18291

 Excited State   3:  2.362-A      3.2171 eV  385.39 nm  f=0.0394  <S**2>=1.145
     74A -> 79A       -0.13160
     72B -> 78B        0.24315
     73B -> 78B        0.35814
     74B -> 79B        0.10018
     75B -> 78B       -0.32660
     76B -> 78B        0.68101
     77B -> 78B        0.10972
     77B -> 81B       -0.11725
     77B -> 84B       -0.11145
Alpha  occ. eigenvalues --   -0.36454  -0.33700  -0.33330  -0.32828  -0.32232
Alpha  occ. eigenvalues --   -0.31483  -0.27682  -0.22517
Alpha virt. eigenvalues --    0.00859   0.01346   0.01603   0.02195   0.02343
  Beta  occ. eigenvalues --   -0.35105  -0.32997  -0.32734  -0.32494  -0.31881
  Beta  occ. eigenvalues --   -0.30134  -0.26044
  Beta virt. eigenvalues --   -0.05621   0.01159   0.01392   0.01795   0.02281
```

1g, X=piperidinyl

```
Excited State   1:  2.143-A      2.3372 eV  530.49 nm  f=0.0566  <S**2>=0.898
     77B -> 78B        0.95261
```

```
 This state for optimization and/or second-order correction.
 Total Energy, E(TD-HF/TD-KS) =  -915.824064826
 Copying the excited state density for this state as the 1-particle RhoCI density.

 Excited State   2:  2.397-A      2.9878 eV  414.96 nm  f=0.0085  <S**2>=1.186
     76A -> 79A        0.10597
     78A -> 79A        0.61347
     78A -> 80A        0.14881
     78A -> 81A        0.37350
     78A -> 84A        0.38871
     78A -> 85A        0.23556
     78A -> 86A       -0.15091
     76B -> 79B       -0.10562
     77B -> 78B        0.14999

 Excited State   3:  2.308-A      3.1728 eV  390.77 nm  f=0.0303  <S**2>=1.082
     73B -> 78B       -0.24931
     73B -> 79B        0.11589
     74B -> 78B        0.24555
     75B -> 78B       -0.50173
     76B -> 78B        0.64222
     77B -> 84B       -0.11122
Alpha  occ. eigenvalues --   -0.37944  -0.35846  -0.32867  -0.32692  -0.32067
Alpha  occ. eigenvalues --   -0.31385  -0.27141  -0.22271
Alpha virt. eigenvalues --    0.00975   0.01398   0.01726   0.02220   0.02377
  Beta  occ. eigenvalues --   -0.37706  -0.34076  -0.32453  -0.32321  -0.31575
  Beta  occ. eigenvalues --   -0.29901  -0.25451
  Beta virt. eigenvalues --   -0.05387   0.01266   0.01458   0.01890   0.02285
```

1h, X=pyrrolidinyl

```
Excited State   1:  2.138-A      2.3215 eV  534.06 nm  f=0.0592  <S**2>=0.893
     73B -> 74B        0.95588
 This state for optimization and/or second-order correction.
 Total Energy, E(TD-HF/TD-KS) =  -876.530583892
 Copying the excited state density for this state as the 1-particle RhoCI density.

 Excited State   2:  2.405-A      2.9901 eV  414.65 nm  f=0.0098  <S**2>=1.196
     72A -> 75A        0.11682
     74A -> 75A        0.60383
     74A -> 76A        0.24682
     74A -> 77A       -0.28713
     74A -> 78A       -0.20496
     74A -> 79A       -0.20122
     74A -> 80A        0.34258
     74A -> 81A        0.16697
     74A -> 82A       -0.15170
     72B -> 76B       -0.12053
     73B -> 74B        0.13556

 Excited State   3:  2.458-A      3.1915 eV  388.48 nm  f=0.0484  <S**2>=1.261
     70A -> 75A       -0.15930
     69B -> 74B        0.31151
     70B -> 76B        0.11340
     71B -> 74B       -0.21549
     72B -> 74B        0.75043
     73B -> 80B       -0.16302
Alpha  occ. eigenvalues --   -0.39416  -0.39097  -0.35806  -0.33200  -0.32715
Alpha  occ. eigenvalues --   -0.32117  -0.31318  -0.27116  -0.22221
Alpha virt. eigenvalues --    0.01009   0.01285   0.01952   0.02146   0.02366
  Beta  occ. eigenvalues --   -0.39251  -0.38517  -0.34048  -0.32601  -0.32352
  Beta  occ. eigenvalues --   -0.31763  -0.29804  -0.25383
  Beta virt. eigenvalues --   -0.05356   0.01276   0.01396   0.02080   0.02189
```

1i, X=NMePh

```
Excited State   1:  2.166-A      2.4130 eV  513.82 nm  f=0.0579  <S**2>=0.923
     83A -> 85A       -0.10547
     83A -> 86A       -0.11285
     81B -> 83B       -0.17624
     82B -> 83B        0.91338
 This state for optimization and/or second-order correction.
 Total Energy, E(TD-HF/TD-KS) =  -990.779811588
 Copying the excited state density for this state as the 1-particle RhoCI
density.

 Excited State   2:  2.327-A      2.9456 eV  420.91 nm  f=0.0037  <S**2>=1.104
     83A -> 84A        0.72016
     83A -> 85A        0.29539
     83A -> 86A        0.29332
     83A -> 93A        0.14709
     83A -> 94A       -0.14877
     83A -> 95A        0.23315
     82B -> 83B        0.20428

 Excited State   3:  2.305-A      3.0794 eV  402.62 nm  f=0.0367  <S**2>=1.078
     78A -> 84A        0.10367
     76B -> 83B        0.44487
     78B -> 83B       -0.12511
     81B -> 83B        0.75629
     82B -> 83B        0.14535
     82B -> 84B        0.10827
     82B -> 86B        0.11539
Alpha  occ. eigenvalues --   -0.33841  -0.33388  -0.32797  -0.32208  -0.31264
Alpha  occ. eigenvalues --   -0.30455  -0.26848  -0.22521
Alpha virt. eigenvalues --    0.00840   0.01055   0.01485   0.01939   0.02152
  Beta  occ. eigenvalues --   -0.33148  -0.32472  -0.32080  -0.31618  -0.31233
  Beta  occ. eigenvalues --   -0.29425  -0.25582
  Beta virt. eigenvalues --   -0.05642   0.01085   0.01168   0.01688   0.02011
```

1k, X=*t*Bu

```
Excited State   1:  2.336-A      2.6825 eV  462.20 nm  f=0.0341  <S**2>=1.114
     70A -> 73A        0.13176
     70A -> 74A       -0.10734
     71A -> 72A        0.52241
     71A -> 73A        0.38076
     71A -> 74A       -0.32133
     71A -> 75A       -0.12495
     66B -> 71B        0.11740
     67B -> 72B       -0.12005
     70B -> 71B        0.53093
 This state for optimization and/or second-order correction.
 Total Energy, E(TD-HF/TD-KS) =  -822.385284227
 Copying the excited state density for this state as the 1-particle RhoCI
density.

 Excited State   2:  2.163-A      2.8654 eV  432.69 nm  f=0.0245  <S**2>=0.919
     71A -> 72A       -0.51618
     71A -> 73A       -0.21029
     71A -> 74A        0.18518
     66B -> 71B        0.12566
     70B -> 71B        0.73775

 Excited State   3:  2.082-A      3.1540 eV  393.10 nm  f=0.0030  <S**2>=0.834
     66B -> 71B        0.60473
     67B -> 71B       -0.19936
     68B -> 71B       -0.42304
     69B -> 71B        0.56432
```

```
     70B -> 71B       -0.10762
Alpha  occ. eigenvalues --   -0.35381  -0.33430  -0.32893  -0.32221  -0.30362
 Alpha  occ. eigenvalues --   -0.22679
 Alpha virt. eigenvalues --    0.00685   0.01055   0.01442   0.02054   0.02274
Beta  occ. eigenvalues --   -0.41368  -0.40252  -0.39126  -0.38146  -0.37303
  Beta  occ. eigenvalues --   -0.32789  -0.32653  -0.32500  -0.31908  -0.28702
  Beta virt. eigenvalues --   -0.05963   0.00952   0.01217   0.01664   0.02165
```

1l, X=*c*-Hex

```
Excited State   1:  2.345-A      2.6818 eV  462.31 nm  f=0.0333  <S**2>=1.125
     77A -> 80A       -0.14845
     78A -> 79A        0.55154
     78A -> 80A        0.38707
     78A -> 81A        0.28192
     78A -> 82A       -0.10494
     75B -> 78B       -0.10545
     75B -> 79B       -0.11359
     77B -> 78B       -0.52302
 This state for optimization and/or second-order correction.
 Total Energy, E(TD-HF/TD-KS) =  -899.772206876
 Copying  the  excited  state  density  for  this  state  as  the  1-particle  RhoCI
density.

 Excited State   2:  2.171-A      2.8697 eV  432.04 nm  f=0.0265  <S**2>=0.929
     78A -> 79A        0.52361
     78A -> 80A        0.20075
     78A -> 81A        0.15730
     73B -> 78B       -0.11926
     77B -> 78B        0.74481

 Excited State   3:  2.085-A      3.2183 eV  385.25 nm  f=0.0026  <S**2>=0.837
     72B -> 78B       -0.11516
     73B -> 78B        0.75589
     74B -> 78B        0.32222
     75B -> 78B        0.13054
     76B -> 78B        0.45372
Alpha  occ. eigenvalues --   -0.36228  -0.35740  -0.34871  -0.33681  -0.32909
 Alpha  occ. eigenvalues --   -0.32256  -0.30368  -0.22754
 Alpha virt. eigenvalues --    0.00633   0.01059   0.01466   0.02077   0.02228
Beta  occ. eigenvalues --   -0.35761  -0.35565  -0.32989  -0.32535  -0.32460
  Beta  occ. eigenvalues --   -0.31985  -0.28736
  Beta virt. eigenvalues --   -0.06025   0.00891   0.01262   0.01654   0.02183
```

1m, X=*c*-Pr

```
Excited State   1:  2.349-A      2.6616 eV  465.83 nm  f=0.0378  <S**2>=1.130
     65A -> 68A       -0.15835
     66A -> 67A        0.49256
     66A -> 68A        0.36277
     66A -> 69A       -0.19800
     64B -> 67B       -0.11013
     65B -> 66B        0.63718
 This state for optimization and/or second-order correction.
 Total Energy, E(TD-HF/TD-KS) =  -781.856479644
 Copying  the  excited  state  density  for  this  state  as  the  1-particle  RhoCI
density.

 Excited State   2:  2.146-A      2.8622 eV  433.18 nm  f=0.0182  <S**2>=0.901
     66A -> 67A       -0.60984
     66A -> 68A       -0.25603
     66A -> 69A        0.14847
     64B -> 66B        0.14436
```

```
     65B -> 66B        0.66033

 Excited State   3:  2.086-A      3.2609 eV  380.22 nm  f=0.0019  <S**2>=0.838
     61B -> 66B        0.81848
     61B -> 78B       -0.11175
     62B -> 66B        0.22651
     63B -> 66B        0.41393
     64B -> 66B       -0.13286
Alpha  occ. eigenvalues --   -0.34160  -0.33835  -0.32890  -0.32268  -0.30150
 Alpha  occ. eigenvalues --   -0.22771
 Alpha virt. eigenvalues --    0.00625   0.01049   0.01507   0.02064   0.0235
Beta  occ. eigenvalues --   -0.42269  -0.40683  -0.39277  -0.36448  -0.36204
  Beta  occ. eigenvalues --   -0.33187  -0.32568  -0.32013  -0.31797  -0.28474
  Beta virt. eigenvalues --   -0.06063   0.00887   0.01309   0.01650   0.02196
```

7. Archive for (U)B3LYP/6-31G(2d,p) geometry optimization results

3-Phenylbenzo[*e*][1,2,4]triazin-4-yl (1a)

```
1\1\GINC-LOCALHOST\FOpt\UB3LYP\6-31G(2d,p)\C19H14N3(2)\PIOTR\14-Apr-20
 16\0\\#P UB3LYP/6-31G(2d,p) FOpt(tight) SCF=Direct #P Geom=(NoDistance
 ,NoAngle) fcheck freq(noraman, readIso)\\Diphenyl Benzotriazinyl\\0,2\
 N,0.0668880214,0.0309630908,-0.1028664636\N,0.0238965915,-0.05474281,1
 .253647311\C,1.1929887729,0.0077038091,1.8969357473\N,2.4025743493,0.
 1425592176,1.3488723893\C,2.4499206899,0.1208091115,-0.0168500117\C,1.
 2704710724,0.0307119382,-0.8073322179\C,1.3616759023,-0.0879239311,-2.
 2003608307\C,2.608360457,-0.0609852868,-2.8129157629\C,3.7754169709,0.
 0763554312,-2.051030983\C,3.6948894705,0.1595754005,-0.6715013337\C,1.
 1093446979,-0.0683596906,3.3791715752\C,-0.1272886442,-0.1278035658,4.
 0359293998\C,-0.1838626196,-0.2019451712,5.4235706815\C,0.9909487953,-
 0.2169543811,6.1750345386\C,2.2243622022,-0.1562248937,5.5282288686\C,
 2.2849487348,-0.0822904591,4.1402957841\C,-1.217092773,0.0582020482,-0
 .7238169603\C,-2.1941947198,-0.8475910628,-0.3044536492\C,-3.460123073
 3,-0.8096014281,-0.8790134768\C,-3.7591852161,0.1276959918,-1.86752227
 69\C,-2.7844733268,1.0376953244,-2.2726126284\C,-1.5152640635,1.011411
 9615,-1.7008194155\H,4.5796797505,0.239921382,-0.0503244882\H,4.742779
 7813,0.1040643328,-2.5411022715\H,2.670595596,-0.1517363438,-3.8920209
 326\H,0.4669832054,-0.2100748445,-2.7959877221\H,3.2359382323,-0.03423
 63774,3.6247199258\H,3.1430040384,-0.1671108823,6.106305805\H,0.944831
 607,-0.2744239091,7.2579980578\H,-1.1479937466,-0.2459065944,5.9202674
 24\H,-1.0364740242,-0.1106496138,3.4478892229\H,-0.7656963371,1.737092
 5576,-1.9929763864\H,-3.0132267659,1.7833599863,-3.0267818837\H,-4.747
 4788553,0.1531115916,-2.3139000976\H,-4.214628561,-1.518401918,-0.5541
 502677\H,-1.948902213,-1.5635660114,0.4697683292\\Version=ES64L-G09Rev
 D.01\State=2-A\HF=-896.7104558\S2=0.766382\S2-1=0.\S2A=0.750163\RMSD=1
 .865e-09\RMSF=6.095e-07\Dipole=-0.6153241,-0.0482827,-0.9597168\Quadru
 pole=3.0821865,-7.7212136,4.6390271,0.9415626,0.932608,-2.5784791\PG=C
 01 [X(C19H14N3)]\\@
```

1a^{+}

```
1\1\GINC-LOCALHOST\FOpt\RB3LYP\6-31G(2d,p)\C19H14N3(1+)\PIOTR\14-Apr-2
 022\0\\#P B3LYP/6-31G(2d,p) FOpt(tight) SCF=Direct #P Geom=(NoDistance
 ,NoAngle) fcheck freq(noraman, readIso)\\Diphenyl Benzotriazinyl\\1,1\
 N,0.0885418611,0.025438077,-0.0790914575\N,0.0413323205,0.0066113134,1
 .2250919811\C,1.2132839024,0.0101686259,1.9135884381\N,2.4092484664,0.
 0961554636,1.3249323481\C,2.4560499417,0.0866204509,-0.0081762405\C,1.
 25458284,0.009060611,-0.7957130954\C,1.3183362456,-0.1033871058,-2.203
 9320971\C,2.5522412536,-0.0829287584,-2.8043787728\C,3.7490096524,0.05
 17432989,-2.0442942264\C,3.7089517758,0.1310275675,-0.6788400669\C,1.1
 170326451,-0.052902476,3.373565113\C,-0.1336235645,-0.0929319119,4.014
 2542791\C,-0.2013655556,-0.1570362377,5.3995123936\C,0.9699401257,-0.1
 821168233,6.1575844303\C,2.2146883471,-0.1419642412,5.5270110594\C,2.2
 92520781,-0.0770226553,4.1426772951\C,-1.2136317325,0.0533839155,-0.71
 8574949\C,-2.1424672203,-0.9216711891,-0.3617389205\C,-3.4030847256,-0
```

.884561823,-0.9477000357\C,-3.7255977705,0.1250746923,-1.8553140507\C,
-2.7882728241,1.1046396988,-2.1835096828\C,-1.5167400548,1.0744401371,
-1.6185223654\H,4.6024491436,0.2065291059,-0.0713309839\H,4.7015971377
,0.0725862927,-2.5618076976\H,2.6196041523,-0.1779079699,-3.8823144611
\H,0.4158326771,-0.2178975708,-2.7875066894\H,3.2541374315,-0.04497349
55,3.6462984824\H,3.1243198553,-0.1612920957,6.1166818678\H,0.91304673
37,-0.2322030688,7.2396484926\H,-1.1678625216,-0.1856625186,5.88998260
9\H,-1.042199684,-0.0683854829,3.42601715\H,-0.7905774174,1.8462853235
,-1.8451281159\H,-3.0469639492,1.9004904601,-2.8723746534\H,-4.7125397
731,0.1522976808,-2.3033572685\H,-4.1331692124,-1.644058005,-0.6928207
826\H,-1.8740372844,-1.6929452859,0.3498756733\\Version=ES64L-G09RevD.
01\State=1-A\HF=-896.4985167\RMSD=2.945e-09\RMSF=8.222e-07\Dipole=0.08
94824,-0.0551886,-1.7766615\Quadrupole=7.2632229,-24.8419766,17.578753
6,1.2195837,1.9312336,-3.6372277\PG=C01 [X(C19H14N3)]\\@

3-Amino-1-phenyl-1,4-dihydrobenzo[*e*][1,2,4]triazin-4-yl (1b)
1\1\GINC-LOCALHOST\FOpt\UB3LYP\6-31G(2d,p)\C13H11N4(2)\PIOTR\08-Mar-20
18\0\\#P UB3LYP/6-31G(2d,p) FOpt(tight) SCF=Direct #P Geom=(NoDistance
,NoAngle) fcheck freq(noraman, readIso)\\1-Ph-Benzotriazinyl-3-NH2\\0,
2\N,-0.9340741252,-0.0756616605,-0.0021459412\N,0.3336635287,-0.578343
1919,-0.0004708758\C,1.3147229887,0.3337837963,0.0807464277\N,1.208612
2427,1.6550268669,0.1820746176\C,-0.0610419863,2.1470193522,0.06887505
62\C,-1.192008781,1.2917753287,-0.0628645588\C,-2.4678470559,1.8371905
245,-0.276059176\C,-2.6355023763,3.2139834331,-0.2979003038\C,-1.53841
18636,4.0671790565,-0.1157264694\C,-0.2717408517,3.5390609691,0.058323
17\C,-1.9464998792,-1.0779821427,0.0149354341\C,-1.8094500486,-2.19511
4931,-0.8135276309\C,-2.7728916267,-3.1973237937,-0.7846459523\C,-3.87
18727201,-3.0970557722,0.0683011897\C,-3.995600431,-1.9890625754,0.904
4728584\C,-3.0349114124,-0.9816427626,0.8867751599\H,0.6003167068,4.17
28320664,0.1730689206\H,-1.6806034218,5.1427048578,-0.1264312929\H,-3.
6251870083,3.6260066398,-0.4630066717\H,-3.3166533849,1.1856596704,-0.
4348644326\H,-3.1146950209,-0.135676346,1.5586013346\H,-4.8354184968,-
1.9112799588,1.5870574093\H,-4.6218592211,-3.8805633353,0.0867571381\H
,-2.6648064923,-4.0593930558,-1.4346748977\H,-0.9451260937,-2.26300128
94,-1.461648887\N,2.5876554136,-0.192728567,0.0319675289\H,3.290895873
9,0.4238088874,0.4112383425\H,2.6593052133,-1.1559164668,0.3245376728\
\Version=ES64L-G09RevD.01\State=2-A\HF=-721.0116236\S2=0.762659\S2-1=0
.\S2A=0.750107\RMSD=9.017e-09\RMSF=1.428e-06\Dipole=-0.5692907,-0.5228
966,0.2612809\Quadrupole=3.699447,1.41376,-5.113207,-1.3292006,1.37897
37,0.9463828\PG=C01 [X(C13H11N4)]\\@

$1b^+$
1\1\GINC-LOCALHOST\FOpt\RB3LYP\6-31G(2d,p)\C13H11N4(1+)\PIOTR\13-Apr-2
022\0\\#P B3LYP/6-31G(2d,p) FOpt(tight) SCF=Direct #P Geom=(NoDistance
,NoAngle) fcheck freq(noraman, readIso)\\1-Ph-Benzotriazinyl-3-NH2 cat
\\1,1\N,-0.9114998221,-0.0656698388,0.0404670504\N,0.2972456525,-0.556
2776921,0.1477479654\C,1.3231348979,0.3375002585,0.186013582\N,1.18641
85076,1.6670961286,0.2036498949\C,-0.0490751223,2.1455187045,0.0673571
15\C,-1.1860437221,1.2664279208,-0.063324829\C,-2.4815481746,1.7899020
059,-0.3078955665\C,-2.6381659283,3.1490061662,-0.3611991045\C,-1.5337
32168,4.0323642719,-0.1731183074\C,-0.2712365894,3.5504693262,0.032137
7222\C,-1.9517378942,-1.0764435941,0.0358525975\C,-1.8470439475,-2.123
6138893,-0.8772462813\C,-2.8241418462,-3.1128601786,-0.8685371881\C,-3
.8687712863,-3.0593344728,0.0550565352\C,-3.9436095162,-2.0142686644,0
.9759565949\C,-2.9837950285,-1.0066837806,0.9712561784\H,0.5885712059,
4.1983109085,0.1503187564\H,-1.7045174934,5.1024497289,-0.2139522698\H
,-3.619645467,3.5649738025,-0.5580174959\H,-3.3190556857,1.1249945553,
-0.464486867\H,-3.0173441204,-0.2049311658,1.6994826628\H,-4.743651063
,-1.9851164063,1.7066568963\H,-4.6232676788,-3.8379483303,0.0614524704
\H,-2.7668118454,-3.9265080008,-1.5823198599\H,-1.0239279142,-2.150765
2089,-1.5806629832\N,2.5528699109,-0.1929012242,0.2329082112\H,3.34522
97857,0.4193965874,0.3418415912\H,2.674122023,-1.1898023182,0.30637009
87\\Version=ES64L-G09RevD.01\State=1-A\HF=-720.8022365\RMSD=8.365e-09\
RMSF=1.496e-06\Dipole=0.2696694,0.2255734,-0.0340805\Quadrupole=9.3485

79,8.657942,-18.006521,2.0413857,-0.0334136,1.0673373\PG=C01 [X(C13H11
N4)]\\@

3-(Morpholin-4-yl)-1-phenyl-1,4-dihydrobenzo[*e*][1,2,4]triazin-4-yl (1c)

1\1\GINC-LOCALHOST\FOpt\UB3LYP\6-31G(2d,p)\C17H17N4O1(2)\PIOTR\08-Mar-
2018\0\\#P UB3LYP/6-31G(2d,p) FOpt(tight) SCF=Direct #P Geom=(NoDistan
ce,NoAngle) fcheck freq(noraman, readIso)\\1-Ph-Benzotriazinyl-3-Morph
olinyl\\0,2\N,-0.944249274,-0.1067350574,0.1422251711\N,0.2979264292,-
0.6266687536,0.3423965039\C,1.286662319,0.2689204791,0.5312580429\N,1.
1753500006,1.5963325023,0.5676941632\C,-0.0546886084,2.1012554013,0.26
40520072\C,-1.1726325333,1.2594983478,0.0061428929\C,-2.3995645822,1.8
12500007,-0.3928003368\C,-2.5388969388,3.189758122,-0.4803803322\C,-1.
4603837079,4.0334820846,-0.1794671569\C,-0.2379640303,3.4954610745,0.1
807340428\C,-1.9674886138,-1.0948426144,0.0431441178\C,-1.7507675269,-
2.220904938,-0.7548730431\C,-2.7273414453,-3.2076098071,-0.8355957623\
C,-3.9191949686,-3.0815934453,-0.1223027488\C,-4.1250828919,-1.9628798
19,0.6829271591\C,-3.1522185088,-0.9710398819,0.7737598609\H,0.6204479
132,4.1224630394,0.3937986876\H,-1.5808336684,5.1098960773,-0.24265827
77\H,-3.4901042243,3.6095722014,-0.7891795338\H,-3.2304702707,1.166345
0518,-0.6426538551\H,-3.2988365285,-0.1149279662,1.421190281\H,-5.0405
521693,-1.8643271805,1.2568727461\H,-4.6789242938,-3.8530161377,-0.188
4538486\H,-2.556696201,-4.0771114462,-1.4618764186\H,-0.8178018599,-2.
3073369669,-1.2971965153\N,2.5412932637,-0.2727576562,0.698292147\O,5.
1477918319,-1.3431974764,0.852908539\C,3.6540871728,0.5639983316,1.131
1637278\C,2.7367902561,-1.6959637843,0.9474099249\C,4.9523726815,0.026
6738689,0.5363869304\C,4.0774972077,-2.1328885801,0.3621481066\H,3.729
2801968,0.5600765442,2.2305326589\H,3.469753535,1.5876201175,0.8078363
553\H,2.7305949327,-1.8973968626,2.0307597093\H,1.9208730169,-2.256822
5211,0.494078242\H,5.8099920756,0.5666899019,0.9469349474\H,4.94135807
74,0.1626176029,-0.5574622557\H,4.2958738703,-3.1662868829,0.645605820
7\H,4.0356780359,-2.0708478074,-0.7378270309\\Version=ES64L-G09RevD.01
\State=2-A\HF=-952.2664018\S2=0.762144\S2-1=0.\S2A=0.750099\RMSD=7.319
e-09\RMSF=1.658e-06\Dipole=-0.9527731,-0.3518925,-0.2447236\Quadrupole
=0.8736889,3.1018145,-3.9755035,3.4020208,-1.4633918,1.9152314\PG=C01
[X(C17H17N4O1)]\\@

1c^{+}

1\1\GINC-LOCALHOST\FOpt\RB3LYP\6-31G(2d,p)\C17H17N4O1(1+)\PIOTR\13-Apr
-2022\0\\#P B3LYP/6-31G(2d,p) FOpt(tight) SCF=Direct #P Geom=(NoDistan
ce,NoAngle) fcheck freq(noraman, readIso)\\1-Ph-Benzotriazinyl-3-Morph
olinyl cat\\1,1\N,-0.9050113489,-0.0986666744,0.1921941685\N,0.2582223
931,-0.6000390123,0.5093727661\C,1.2987290479,0.2743444585,0.673738603
8\N,1.1605769265,1.6121307605,0.6285270212\C,-0.0260787052,2.096888907
4,0.2818209863\C,-1.1427289719,1.2291758448,-0.0016821919\C,-2.3774733
585,1.7553396339,-0.4615876585\C,-2.505252242,3.1128368111,-0.57969904
78\C,-1.4296996891,3.990261407,-0.2458430623\C,-0.2229539628,3.5039985
369,0.1709883121\C,-1.9517900727,-1.0966692661,0.0594721069\C,-1.74472
57353,-2.1548879412,-0.8218012496\C,-2.7306621581,-3.1293901909,-0.934
5812031\C,-3.8885135812,-3.0484402899,-0.1602602481\C,-4.0682194298,-1
.9906533643,0.7309367491\C,-3.0985397032,-0.9985434914,0.8454608975\H,
0.6147958944,4.1488676215,0.4059710072\H,-1.5764916664,5.060274826,-0.
3438244129\H,-3.4377587821,3.5325132386,-0.9390680394\H,-3.189705979,1
.0926797148,-0.7254127731\H,-3.216430859,-0.1849249941,1.5514613564\H,
-4.9594042037,-1.9386565836,1.3457330304\H,-4.6505391344,-3.814734969,
-0.2477138228\H,-2.5924588933,-3.9521028334,-1.6266355916\H,-0.8367713
288,-2.2016008233,-1.4106451279\N,2.5016889607,-0.2639508075,0.9256698
255\O,5.0982798951,-1.3368366948,0.7012650489\C,3.6790317558,0.5682405
44,1.2152436326\C,2.7283799296,-1.7070208829,1.0913423256\C,4.88229656
16,0.03566527,0.4359566078\C,3.9840671673,-2.1162805776,0.3184461506\H
,3.884511962,0.516327043,2.2918871097\H,3.4626837347,1.6011563823,0.94
97197053\H,2.8701389323,-1.9154275479,2.1592201951\H,1.8588341152,-2.2
597384381,0.7413194818\H,5.7854269821,0.5662161426,0.74544015\H,4.7306
720449,0.1998203683,-0.6426450325\H,4.2300646114,-3.1568719867,0.54198
12547\H,3.8017388609,-2.0232949713,-0.7642433615\\Version=ES64L-G09Rev

D.01\State=1-A\HF=-952.0604458\RMSD=2.898e-09\RMSF=5.177e-07\Dipole=-1
.8039926,0.6610847,-0.389573\Quadrupole=10.4115249,8.0451997,-18.45672
46,2.6322593,3.2279184,0.8292569\PG=C01 [X(C17H17N4O1)]\\@

3-(Piperidin-1-yl)-1-phenyl-1,4-dihydrobenzo[*e*][1,2,4]triazin-4-yl (1g)

1\1\GINC-LOCALHOST\FOpt\UB3LYP\6-31G(2d,p)\C18H19N4(2)\PIOTR\10-Apr-20
22\0\\#P UB3LYP/6-31G(2d,p) FOpt(tight) SCF=Direct #P Geom=(NoDistance
,NoAngle) fcheck freq(noraman, readIso)\\1-Ph-Benzotriazinyl-3-Piperid
ine\\0,2\N,-1.0474907792,-0.0680845295,0.0412538027\N,0.2135613673,-0.
5699286267,0.1430428532\C,1.2054489593,0.3387141982,0.2541264262\N,1.0
727650686,1.6656027647,0.2945757514\C,-0.1827387002,2.1510517113,0.083
3561409\C,-1.3059372294,1.2937683167,-0.0848029674\C,-2.5667207248,1.8
281571672,-0.393655312\C,-2.7316240518,3.202875281,-0.4768458613\C,-1.
6450040278,4.0623494006,-0.2612528407\C,-0.3917550177,3.542702571,0.00
79911157\C,-2.0597830824,-1.0715114479,0.024696004\C,-1.8796565352,-2.
2066224111,-0.7701171156\C,-2.8442658454,-3.2081707136,-0.7721871066\C
,-3.9882784102,-3.08895613,0.0166516573\C,-4.1569999997,-1.9615442112,
0.8183771423\C,-3.1955391865,-0.9546528246,0.8308531243\H,0.471600035,
4.1823274407,0.1528153361\H,-1.7845099344,5.1368045305,-0.3197089952\H
,-3.7089965386,3.6081407975,-0.7154600956\H,-3.4049865944,1.1696590279
,-0.5781856344\H,-3.3120922271,-0.0917814666,1.4752994563\H,-5.0336560
434,-1.8676060623,1.4507930729\H,-4.7389644663,-3.8719933651,0.0116874
129\H,-2.702022408,-4.0842400517,-1.396481955\H,-0.9832184556,-2.28769
48618,-1.3714861934\N,2.473645982,-0.183650172,0.3318797146\C,3.625106
1088,0.6771710229,0.5803227815\C,2.7204529148,-1.6113948066,0.50425308
63\C,4.8069854399,0.2689796644,-0.3061759711\C,3.8809186596,-2.0720354
946,-0.3850195576\H,3.9184149537,0.599002796,1.6412842489\H,3.32342227
72,1.7063809712,0.3978342353\H,2.9686904428,-1.812862292,1.5605265077\
H,1.8065320045,-2.153906121,0.2739228345\H,5.6756658033,0.8869469244,-
0.0522818287\H,4.5535041381,0.4805555819,-1.3527152962\H,4.086136043,-
3.130241535,-0.1870989189\H,3.5748997878,-1.9917903828,-1.4359072233\C
,5.1338723677,-1.2208344862,-0.1456919699\H,5.9373220407,-1.5162753386
,-0.8293053375\H,5.5047349242,-1.4025971676,0.8730528757\\Version=ES64
L-G09RevD.01\State=2-A\HF=-916.37705\S2=0.762057\S2-1=0.\S2A=0.750098\
RMSD=9.355e-09\RMSF=1.769e-06\Dipole=-0.3271024,-0.6072141,-0.0771367\
Quadrupole=6.5384482,-0.5185029,-6.0199452,0.0460606,-0.7756266,1.0988
784\PG=C01 [X(C18H19N4)]\\@

1g^{+}

1\1\GINC-LOCALHOST\FOpt\RB3LYP\6-31G(2d,p)\C18H19N4(1+)\PIOTR\11-Apr-2
022\0\\#P B3LYP/6-31G(2d,p) FOpt(tight) SCF=Direct #P Geom=(NoDistance
,NoAngle) fcheck freq(noraman, readIso)\\1-Ph-Benzotriazinyl-3-Piperid
ine cat start at the rad geo\\1,1\N,-0.9953992108,-0.060794818,0.07966
03133\N,0.1954188553,-0.5440245365,0.3079609165\C,1.2366840695,0.34461
13453,0.3849861656\N,1.0717425292,1.6812592209,0.3415128319\C,-0.14472
8357,2.1459057784,0.0864755371\C,-1.267644891,1.2615803096,-0.10417303
67\C,-2.5417558909,1.7671486698,-0.4708964011\C,-2.6982032858,3.121681
0525,-0.5867813668\C,-1.6126862822,4.0162952817,-0.3419586815\C,-0.370
6644316,3.5499119284,-0.0170036969\C,-2.0344690003,-1.0747432595,0.034
8494584\C,-1.8786195534,-2.138114429,-0.8505971649\C,-2.8557004711,-3.
1274931576,-0.881159555\C,-3.9536088822,-3.0556076398,-0.0232365997\C,
-4.0814419031,-1.9923337288,0.8702644734\C,-3.1203249141,-0.9858784193
,0.9038664929\H,0.4734554565,4.2079160511,0.1485241367\H,-1.7820383979
,5.0834773974,-0.4344480303\H,-3.6615935483,3.5257247518,-0.8756405796
\H,-3.3620389859,1.0912591044,-0.6678039548\H,-3.1970419618,-0.1674393
11,1.6099499596\H,-4.9248591832,-1.9469805205,1.5496103407\H,-4.708827
6592,-3.833188812,-0.0471567288\H,-2.757279241,-3.9547593152,-1.574600
872\H,-1.0159013949,-2.1779091313,-1.5043382938\N,2.4613067248,-0.1739
656908,0.5485717368\C,3.6533248769,0.6798918357,0.6865465279\C,2.72430
47426,-1.6184811591,0.6592433442\C,4.7202948835,0.2838860031,-0.340089
5031\C,3.7752010531,-2.0544152554,-0.3680247985\H,4.0403654729,0.54523
50195,1.7057054123\H,3.3519689194,1.7186245242,0.5758667753\H,3.091463
7489,-1.8095243137,1.6766295998\H,1.7915072659,-2.1629399102,0.5349779
457\H,5.6128816929,0.8920207432,-0.1635738003\H,4.356443475,0.53132982

1,-1.3450204437\H,3.9933233725,-3.1154321957,-0.2110424114\H,3.3507962
595,-1.9589839449,-1.3753750179\C,5.050479691,-1.2109233696,-0.2540915
818\H,5.7602123894,-1.4892302338,-1.0384005212\H,5.543091027,-1.421780
0169,0.7044264718\\Version=ES64L-G09RevD.01\State=1-A\HF=-916.1755971\
RMSD=3.197e-09\RMSF=5.193e-07\Dipole=-1.1414456,0.3629581,-0.1503704\Q
uadrupole=17.3033168,4.1390757,-21.4423925,-1.028849,1.0439766,0.40720
44\PG=C01 [X(C18H19N4)]\\@

3-(Pyrrolidyn-1-yl)-1-phenyl-1,4-dihydrobenzo[e][1,2,4]triazin-4-yl (1h)
1\1\GINC-LOCALHOST\FOpt\UB3LYP\6-31G(2d,p)\C17H17N4(2)\PIOTR\10-Apr-20
22\0\\#P UB3LYP/6-31G(2d,p) FOpt(tight) SCF=Direct #P Geom=(NoDistance
,NoAngle) fcheck freq(noraman, readIso)\\1-Ph-Benzotriazinyl-3-Pyrroli
dine\\0,2\N,-1.1457454647,-0.0691414635,0.1107930195\N,0.077037281,-0.
5896185276,0.4120657614\C,1.0235046742,0.311700951,0.752028173\N,0.901
2474213,1.6400492361,0.8232063781\C,-0.2918664454,2.142435561,0.395818
2111\C,-1.3662132528,1.3008134669,-0.0119469976\C,-2.5420435017,1.8589
342542,-0.5380783979\C,-2.6846448744,3.2369926792,-0.6029401593\C,-1.6
570398962,4.0784877567,-0.1534884033\C,-0.4798356727,3.5379275787,0.33
06855121\C,-2.1462759659,-1.0578311504,-0.1150033556\C,-1.8303599227,-
2.1848202872,-0.8789345251\C,-2.787347172,-3.1736682776,-1.078865441\C
,-4.0586513249,-3.0512472252,-0.5185785657\C,-4.3641447095,-1.93343578
95,0.255678691\C,-3.4127204679,-0.9390147358,0.4650704414\H,0.34244276
73,4.1634931831,0.6593864451\H,-1.7799024644,5.1556121164,-0.199427805
9\H,-3.597093964,3.6590149021,-1.0101494605\H,-3.3309692322,1.21535365
85,-0.9037242845\H,-3.6410952669,-0.0849794097,1.0910084338\H,-5.34316
03191,-1.8375826471,0.713619052\H,-4.8024690527,-3.8246166483,-0.67788
13939\H,-2.5379172564,-4.0431051504,-1.6783945289\H,-0.8357284019,-2.2
703861525,-1.2971609535\N,2.2312692542,-0.2241922509,1.0715660073\C,3.
382806865,0.5941793502,1.4461667847\C,2.5015980023,-1.659093615,1.1133
180178\C,4.540500513,-0.41442619,1.4469683999\C,3.858766253,-1.7390919
787,1.8280631952\H,3.2352561613,1.0451814565,2.4371695195\H,3.52071517
28,1.415957208,0.739153607\H,2.5542133914,-2.0777058228,0.0981653437\H
,1.7032212907,-2.1904015906,1.6383241669\H,4.9711140008,-0.488992548,0
.4421253692\H,5.341338546,-0.1272529512,2.1324594101\H,4.4320251285,-2
.6219825431,1.5352975451\H,3.7057810159,-1.7841573135,2.9120597079\\Ve
rsion=ES64L-G09RevD.01\State=2-A\HF=-877.0580365\S2=0.762253\S2-1=0.\S
2A=0.750102\RMSD=8.394e-09\RMSF=1.345e-06\Dipole=-0.0991661,-0.6646589
,-0.072473\Quadrupole=7.2007053,-1.3789542,-5.8217511,-1.2850623,1.982
3927,0.942756\PG=C01 [X(C17H17N4)]\\@

1h
1\1\GINC-LOCALHOST\FOpt\RB3LYP\6-31G(2d,p)\C17H17N4(1+)\PIOTR\11-Apr-2
022\0\\#P B3LYP/6-31G(2d,p) FOpt(tight) SCF=Direct #P Geom=(NoDistance
,NoAngle) fcheck freq(noraman, readIso)\\1-Ph-Benzotriazinyl-3-Pyrroli
dine cation start at rad geo\\1,1\N,-1.1158297225,-0.0589219461,0.1229
996828\N,0.0310170892,-0.5659092792,0.4898657411\C,1.0419168483,0.3161
84896,0.7675691807\N,0.9024920964,1.6559974586,0.7662816085\C,-0.26550
95979,2.1418416861,0.3599731949\C,-1.3521836409,1.2756367095,-0.028877
9569\C,-2.5588844673,1.8107669632,-0.549282535\C,-2.6935567855,3.17063
8997,-0.6237395413\C,-1.6521221704,4.0434530195,-0.1865832858\C,-0.470
320613,3.5506689831,0.2892102826\C,-2.1418622238,-1.0572437369,-0.1198
504297\C,-1.8635367721,-2.0848270995,-1.0176570612\C,-2.8308047397,-3.
061004919,-1.2322791587\C,-4.0418437882,-3.012757865,-0.5412138921\C,-
4.2933670151,-1.9865184245,0.3691425467\C,-3.3424227774,-0.993369673,0
.5853589734\H,0.3442095199,4.1917626606,0.6030429129\H,-1.803627951,5.
1152309365,-0.252936179\H,-3.6048649332,3.5957142695,-1.02835609\H,-3.
3445646183,1.1528329057,-0.8928860185\H,-3.5177567819,-0.2051959027,1.
3081210096\H,-5.2265192801,-1.9604356665,0.9200145542\H,-4.7891949298,
-3.7802853537,-0.7083552686\H,-2.636021835,-3.8598973468,-1.9385041901
\H,-0.9136843677,-2.1080489399,-1.5375636586\N,2.2173380055,-0.2196328
776,1.0916541385\C,3.4020850367,0.5961219812,1.4192918405\C,2.47464158
67,-1.6683867205,1.1941574092\C,4.5356428241,-0.43652268,1.4542790793\
C,3.8349012067,-1.7297083636,1.9017405434\H,3.2551329049,1.0842084513,
2.3897806398\H,3.5407695371,1.3799392393,0.6719407714\H,2.5141466814,-

2.1095818529,0.1911144166\H,1.6713074887,-2.1611809413,1.7454973201\H,
4.9596443443,-0.5596261813,0.4526022882\H,5.3419598643,-0.1323060023,2
.1234042155\H,4.3892754578,-2.632336296,1.6400181935\H,3.6896116289,-1
.7279119985,2.9866476421\\Version=ES64L-G09RevD.01\State=1-A\HF=-876.8
573936\RMSD=5.493e-09\RMSF=1.923e-06\Dipole=-0.6564399,0.2838451,-0.29
25784\Quadrupole=14.6860209,4.0605343,-18.7465552,-1.3564659,8.3774685
,0.7053159\PG=C01 [X(C17H17N4)]\\@

3-(*N*-methyl-*N*-phenylamino)-1-phenyl-1,4-dihydrobenzo[*e*][1,2,4]triazin-4-yl (1i)

1\1\GINC-LOCALHOST\FOpt\UB3LYP\6-31G(2d,p)\C20H17N4(2)\PIOTR\10-Apr-20
22\0\\#P UB3LYP/6-31G(2d,p) FOpt(tight) SCF=Direct #P Geom=(NoDistance
,NoAngle) fcheck freq(noraman, readIso)\\1-Ph-Benzotriazinyl-3-NMePh\\
0,2\N,-1.5185577764,-0.0572036366,-0.0124683239\N,-0.4514617447,-0.903
5754616,0.0167147574\C,0.7590538485,-0.3201820919,0.0773652214\N,1.027
0595876,0.9843595205,0.0968791093\C,-0.0429198392,1.8130558722,-0.0614
562288\C,-1.3750025456,1.3196292151,-0.1528799647\C,-2.4397538111,2.19
74478289,-0.4097562373\C,-2.199194494,3.5591208074,-0.5175326987\C,-0.
8983678732,4.0650628304,-0.3801748174\C,0.161396849,3.2040582099,-0.16
22663853\C,-2.7775155555,-0.7225136302,0.0499184999\C,-2.9837677113,-1
.8656722423,-0.7265667644\C,-4.1969146601,-2.541207433,-0.6509237556\C
,-5.2064612722,-2.0877382958,0.1977728688\C,-4.9903808971,-0.95573963,
0.9814542183\C,-3.778409936,-0.2737929274,0.916164266\H,1.1813778285,3
.5619208458,-0.0800962841\H,-0.7216280386,5.1325770057,-0.4596821576\H
,-3.0272058997,4.2311486999,-0.7156092511\H,-3.4428308966,1.812274024,
-0.5354919768\H,-3.5990419864,0.5887977622,1.5464394092\H,-5.761533210
8,-0.6061487796,1.6599531817\H,-6.1515984534,-2.6171468472,0.253493308
2\H,-4.3541178072,-3.4247015601,-1.2609307824\H,-2.1868196138,-2.21034
94,-1.3729174313\N,1.8128206463,-1.214602095,0.1294921219\C,3.17282434
77,-0.7953051435,0.1054322063\C,4.0590198312,-1.3846972183,-0.80309523
06\C,3.6618114978,0.1500525858,1.0148910288\C,5.4088915006,-1.03954709
1,-0.8004351884\C,5.0077984609,0.498815627,1.0036464319\C,5.8899758911
,-0.0937104325,0.1002733044\C,1.5262131162,-2.6429244204,0.0238584291\
H,3.6890742018,-2.1078861257,-1.5217297642\H,2.9816789237,0.6075903219
,1.7198911617\H,6.0803242362,-1.5076720614,-1.5132920444\H,5.371168772
2,1.2327452946,1.7161207748\H,6.9401728424,0.1786529373,0.1008236739\H
,0.7102685149,-2.9081037034,0.6978474569\H,1.2286084637,-2.9379303565,
-0.99037702\H,2.4227446626,-3.1943878053,0.3070528771\\Version=ES64L-G
09RevD.01\State=2-A\HF=-991.371619\S2=0.762639\S2-1=0.\S2A=0.750106\RM
SD=8.214e-09\RMSF=1.379e-06\Dipole=-0.8635045,-0.3666809,-0.0469118\Qu
adrupole=5.5243691,0.7890882,-6.3134573,-0.0372121,-1.4923056,3.746462
4\PG=C01 [X(C20H17N4)]\\@

$1i^+$

1\1\GINC-LOCALHOST\FOpt\RB3LYP\6-31G(2d,p)\C20H17N4(1+)\PIOTR\13-Apr-2
022\0\\#P B3LYP/6-31G(2d,p) FOpt(tight) SCF=Direct #P Geom=(NoDistance
,NoAngle) fcheck freq(noraman, readIso)\\1-Ph-Benzotriazinyl-3-NMePh s
econd orient\\1,1\N,-1.4838165884,-0.0694401998,-0.0277926795\N,-0.479
8706199,-0.8851605434,0.1384751417\C,0.7747079931,-0.3410397137,0.1274
909531\N,1.0264235414,0.9768671634,0.0809426746\C,-0.0127192947,1.7862
395123,-0.1156106052\C,-1.3545005322,1.2758504791,-0.2337247905\C,-2.4
341867864,2.1360247219,-0.5574733834\C,-2.1858557884,3.4759834056,-0.6
934351063\C,-0.8754003825,4.0071686908,-0.5105232694\C,0.1856143395,3.
1911402036,-0.2320642158\C,-2.7772516803,-0.7247566302,0.0245078638\C,
-2.9762584164,-1.847902726,-0.7754432478\C,-4.2021625348,-2.5008620684
,-0.7124563339\C,-5.1961310975,-2.0455574793,0.1544113389\C,-4.9691100
273,-0.9338179571,0.9655470457\C,-3.7536663344,-0.2588400842,0.9043583
916\H,1.1950537503,3.5668720645,-0.1188141567\H,-0.7225125937,5.075664
8763,-0.6154391652\H,-2.9977564829,4.1460913379,-0.951542411\H,-3.4262
607729,1.734885303,-0.7095611022\H,-3.5573025512,0.5892277777,1.549394
7062\H,-5.733608566,-0.5942126612,1.6548160303\H,-6.147703459,-2.56299
45292,0.2035118337\H,-4.3787515968,-3.3674796947,-1.3390600035\H,-2.18
5582077,-2.1930222666,-1.4299543437\N,1.8022323373,-1.2016083729,0.216

1949357\C,1.5947045672,-2.6288445357,0.2648286276\C,1.4997693219,-3.27
04297561,1.4981257308\C,1.5419126959,-3.3555213805,-0.9235856769\C,1.3
462822224,-4.654440147,1.5397156744\C,1.3874856996,-4.7395139843,-0.87
43467025\C,1.2894306997,-5.3882119971,0.3555947361\C,3.1737387509,-0.6
763747425,0.2657567327\H,1.5478646311,-2.6900478912,2.4131846003\H,1.6
317583046,-2.8407711418,-1.8743980143\H,1.273824471,-5.1578650438,2.49
74804824\H,1.3507898241,-5.3092660297,-1.7964242439\H,1.1739020253,-6.
4659164476,0.3917247243\H,3.3897585859,-0.0735115751,-0.6201789872\H,3
.320026934,-0.0513780789,1.15090612\H,3.8517216877,-1.5269228879,0.302
581235\\Version=ES64L-G09RevD.01\State=1-A\HF=-991.1693467\RMSD=8.320e
-09\RMSF=1.127e-06\Dipole=-1.0190917,1.2501412,-0.2362407\Quadrupole=6
.3340171,13.9864009,-20.3204179,-4.370839,0.1774932,-1.4548854\PG=C01
[X(C20H17N4)]\\@

3-(*tert*-butyl)-1-phenyl-1,4-dihydrobenzo[*e*][1,2,4]triazin-4-yl (1k)

1\1\GINC-LOCALHOST\FOpt\UB3LYP\6-31G(2d,p)\C17H18N3(2)\PIOTR\28-Sep-20
22\0\\#P UB3LYP/6-31G(2d,p) FOpt(tight) SCF=Direct #P Geom=(NoDistance
,NoAngle) fcheck freq(noraman, readIso)\\1-Ph-Benzotriazinyl-3-t-Bu\\0
,2\N,-0.4998243165,-0.2511151585,2.3756400991\N,0.4869962571,-0.650295
615,1.5185624655\C,1.3479732275,0.2809214359,1.1235883375\N,1.35369801
67,1.5701460566,1.4757064058\C,0.2999910057,1.9910678967,2.2365657186\
C,-0.7009772466,1.0893231428,2.6948307691\C,-1.8206400275,1.5712483409
,3.3875378737\C,-1.921135594,2.9260200742,3.6755788716\C,-0.918078922,
3.8181285027,3.2742348766\C,0.172680878,3.3555448111,2.5593204038\C,-1
.3132784921,-1.3173564404,2.8576869553\C,-1.7571035944,-2.2931527028,1
.9615048464\C,-2.5236538022,-3.356983148,2.4244700874\C,-2.8493463278,
-3.4575415068,3.7768505133\C,-2.3922580802,-2.4896169694,4.6691730886\
C,-1.620467719,-1.422663171,4.2172031842\H,0.9529085171,4.0221257701,2
.2098297273\H,-1.0039942698,4.8730333931,3.5121982394\H,-2.7898934356,
3.2905302663,4.213228611\H,-2.607996886,0.8927966325,3.6875786595\H,-1
.2395997106,-0.6869084289,4.9153134686\H,-2.6226895117,-2.5693817244,5
.7264155644\H,-3.4483807455,-4.2885200535,4.133605872\H,-2.8696472657,
-4.1093263148,1.7233577774\H,-1.4865740426,-2.2076176366,0.9169126116\
C,3.8151464485,0.0573458751,0.9100555816\C,2.3383441258,-1.6607979622,
-0.187913417\C,2.4702416199,-0.177239375,0.1872695228\C,2.4198767893,0
.6872093728,-1.0910290269\H,3.8884351004,-0.5590057951,1.8124126299\H,
3.9208750846,1.1047702103,1.2012799093\H,2.3705432927,-2.3039581468,0.
6947450767\H,1.3970999688,-1.8610835055,-0.7071707754\H,1.4831903944,0
.528928529,-1.6364842203\H,2.4961219879,1.7483540714,-0.8450138557\H,3
.246665562,0.4204100058,-1.7580178399\H,4.6482713516,-0.2093136691,0.2
508584175\H,3.1616945317,-1.9435289834,-0.85236658\\Version=ES64L-G09R
evD.01\State=2-A\HF=-822.9128015\S2=0.764526\S2-1=0.\S2A=0.750141\RMSD
=3.204e-09\RMSF=2.526e-06\Dipole=-0.8879222,-0.2726966,0.5519853\Quadr
upole=-1.535463,1.4479821,0.0874809,1.6461705,-3.1263426,1.2137081\PG=
C01 [X(C17H18N3)]\\@

$1k^+$

1\1\GINC-LOCALHOST\FOpt\RB3LYP\6-31G(2d,p)\C17H18N3(1+)\PIOTR\19-Oct-2
022\0\\#P B3LYP/6-31G(2d,p) FOpt(tight) SCF=Direct #P Geom=(NoDistance
,NoAngle) fcheck freq(noraman, readIso)\\1-Ph-Benzotriazinyl-3-t-Bu ca
tion\\1,1\N,-0.4835385034,-0.2398520016,2.362862171\N,0.5006087169,-0.
6369688329,1.5873974892\C,1.3611798031,0.2893892271,1.1188293239\N,1.3
09858929,1.5784180947,1.4529413816\C,0.2895981853,1.9936155232,2.20757
80064\C,-0.7109753424,1.0708552899,2.6741487246\C,-1.8457248686,1.5361
848177,3.3796058591\C,-1.9367380637,2.8774164999,3.6550127721\C,-0.923
4060395,3.7950215408,3.254565992\C,0.1645131037,3.3695627315,2.5428248
444\C,-1.317859054,-1.3164828215,2.8550547188\C,-1.8181663345,-2.23537
78913,1.9339529566\C,-2.5927004055,-3.2911863079,2.4007149852\C,-2.838
961018,-3.4306517153,3.7672531179\C,-2.3110025386,-2.513809097,4.67639
31469\C,-1.5460474563,-1.4424280455,4.2259452378\H,0.9379117814,4.0439
626377,2.1960655963\H,-1.0347207827,4.8442647956,3.5040455642\H,-2.805
629628,3.2512086759,4.1852900341\H,-2.6268702497,0.8511961915,3.677489
5229\H,-1.1049262571,-0.7442593651,4.9275736791\H,-2.483311555,-2.6365
169316,5.7394387906\H,-3.4376168217,-4.2606491758,4.1254018132\H,-3.00

13773272,-4.006127245,1.6960128172\H,-1.608198065,-2.1143107715,0.8785
162224\C,3.8193130833,0.0693066058,0.9335726317\C,2.3389481977,-1.6681
960554,-0.1508176026\C,2.4787252195,-0.1789251913,0.1997751439\C,2.427
390403,0.6752223277,-1.0888669882\H,3.8894552577,-0.5274332508,1.84845
03133\H,3.9402307684,1.1235947906,1.1937987962\H,2.3899904772,-2.30402
49675,0.736372767\H,1.3954086529,-1.8779657587,-0.663104543\H,1.492167
3697,0.5178540518,-1.635732741\H,2.5256235153,1.7399258653,-0.86621647
08\H,3.2516338394,0.3845129834,-1.745620543\H,4.6465211037,-0.22003682
33,0.2797276538\H,3.1539060741,-1.953812321,-0.8207327346\\Version=ES6
4L-G09RevD.01\State=1-A\HF=-822.699208\RMSD=6.805e-09\RMSF=2.352e-06\D
ipole=-1.3224787,0.685363,0.9696163\Quadrupole=-2.2158306,8.4536553,-6
.2378247,4.4125461,-12.2515908,2.6016966\PG=C01 [X(C17H18N3)]\\

3-(*cyclo*hexyl)-1-phenyl-1,4-dihydrobenzo[*e*][1,2,4]triazin-4-yl (1l)

1\1\GINC-LOCALHOST\FOpt\UB3LYP\6-31G(2d,p)\C19H20N3(2)\PIOTR\02-Oct-20
22\0\\#P UB3LYP/6-31G(2d,p) FOpt(tight) SCF=Direct #P Geom=(NoDistance
,NoAngle) fcheck\\1-Ph-Benzotriazinyl-3-Cyclohexyl\\0,2\N,-1.177897303
2,-0.1127339958,-0.0540829486\N,0.0338754078,-0.744477574,-0.104236510
4\C,1.1113327046,0.0339403785,-0.0158779789\N,1.1427220736,1.360788416
9,0.1162106233\C,-0.0659552995,1.9974215773,0.0458182973\C,-1.28634721
58,1.2761521475,-0.0792118565\C,-2.4957011219,1.9640852404,-0.25318201
44\C,-2.5061578817,3.3526858513,-0.24270872\C,-1.3185727989,4.07480788
19,-0.0679661926\C,-0.1157334619,3.4036674383,0.0670497731\C,-2.292109
4266,-1.0000127731,-0.0195560954\C,-2.3027108818,-2.1120165628,-0.8658
261637\C,-3.3676023386,-3.0049406965,-0.8173595186\C,-4.4224433651,-2.
7999924468,0.0716286956\C,-4.4000735051,-1.6978769971,0.9242017313\C,-
3.3370166664,-0.7997948548,0.8872748777\H,0.8252622592,3.9309140052,0.
17561276\H,-1.3405112626,5.1592931205,-0.0531177232\H,-3.4466269379,3.
8764981546,-0.3762877183\H,-3.4157602651,1.4158825908,-0.4049609644\H,
-3.3052255358,0.0396703993,1.5713246729\H,-5.2052788979,-1.5402581012,
1.6341622866\H,-5.2516548883,-3.498524105,0.10501533\H,-3.3730762639,-
3.8639713377,-1.4801979415\H,-1.4707734019,-2.2643910292,-1.5413515581
\C,3.3307798438,-0.2051497758,-1.193761024\C,3.1621420772,-0.554194327
6,1.3144209032\C,4.6934319853,-0.9105728487,-1.1964588057\C,4.52467933
45,-1.2589965824,1.3048856641\C,5.408177484,-0.7623726384,0.1530268013
\C,2.4344384952,-0.6997741415,-0.0396008221\H,3.46842957,0.8772277298,
-1.0853952009\H,2.8199071235,-0.3626616162,-2.1512010273\H,3.295010543
3,0.5146120535,1.5218962251\H,2.5309366222,-0.9561304499,2.115567224\H
,5.3172384593,-0.511457097,-2.0047198014\H,4.5505051462,-1.9787988889,
-1.4134849029\H,5.029226361,-1.1060954833,2.2660751479\H,4.373374833,-
2.3430668982,1.2030807824\H,6.3598600959,-1.306263975,0.1415381256\H,5
.6544163286,0.2959989653,0.3171151981\H,2.2041869714,-1.7597007541,-0.
1990566306\\Version=ES64L-G09RevD.01\State=2-A\HF=-900.3411173\S2=0.76
4485\S2-1=0.\S2A=0.750139\RMSD=4.250e-09\RMSF=8.924e-07\Dipole=-0.9914
45,-0.1022413,-0.0542133\Quadrupole=4.0502877,0.8971111,-4.9473988,0.3
244879,-1.3397823,1.6586876\PG=C01 [X(C19H20N3)]\\@

1l^{+}

1\1\GINC-LOCALHOST\FOpt\RB3LYP\6-31G(2d,p)\C19H20N3(1+)\PIOTR\18-Oct-2
022\0\\#P B3LYP/6-31G(2d,p) FOpt(tight) SCF=Direct #P Geom=(NoDistance
,NoAngle) fcheck freq(noraman, readIso)\\1-Ph-Benzotriazinyl-3-Cyclohe
xyl cqtion\\1,1\N,-1.1518212909,-0.101942672,-0.0577965676\N,0.0115179
421,-0.7111119412,-0.0534381494\C,1.1297425662,0.0491410422,-0.0398568
482\N,1.1227325129,1.3783424837,0.0328453526\C,-0.0590680399,2.0015616
719,-0.0097392582\C,-1.2872316793,1.2580420484,-0.1019060886\C,-2.5241
837554,1.9278804242,-0.2530823747\C,-2.5268138618,3.300193951,-0.25248
83617\C,-1.326127953,4.0507171907,-0.1007831605\C,-0.1165563886,3.4212
37919,0.0130633726\C,-2.2888105052,-0.998634128,-0.0198196568\C,-2.338
2876059,-2.0400988854,-0.9447999074\C,-3.4090754137,-2.9253343454,-0.8
930027936\C,-4.3954832431,-2.7779441455,0.0829693894\C,-4.3166521384,-
1.7428893876,1.0147739812\C,-3.2603966553,-0.838427617,0.9686704615\H,
0.8185894321,3.9608302331,0.0998829468\H,-1.3788388412,5.1336658398,-0
.0965955538\H,-3.4656645604,3.828066601,-0.3778716589\H,-3.4399733891,
1.3685710596,-0.3816864751\H,-3.1741994593,-0.0510124973,1.7082184649\

H,-5.0708111635,-1.6432614167,1.7868894436\H,-5.2239376142,-3.47627446
99,0.1222178092\H,-3.4701937088,-3.7320221197,-1.6143165594\H,-1.55734
51275,-2.1430019572,-1.6880653715\C,3.3530849015,-0.196714183,-1.19518
85778\C,3.1422593325,-0.53835982,1.323691829\C,4.7023898772,-0.9274428
839,-1.171221479\C,4.4935557012,-1.2644562358,1.3297645737\C,5.3989526
686,-0.7866167813,0.1878117147\C,2.4372292948,-0.6872706164,-0.0491995
366\H,3.5109967361,0.8824291321,-1.0814139417\H,2.8549734138,-0.347340
9058,-2.1599779053\H,3.2903391248,0.530349888,1.5241183141\H,2.4945273
846,-0.9281846955,2.1172260873\H,5.3388872878,-0.5360311628,-1.9712695
404\H,4.542977291,-1.9913149459,-1.3931380053\H,4.9813849304,-1.109236
6507,2.2975637574\H,4.3239643622,-2.3455662453,1.2368197436\H,6.336560
5588,-1.3511729398,0.1903598553\H,5.667619268,0.265663233,0.3521217203
\H,2.2018928074,-1.7456090682,-0.2060480456\\Version=ES64L-G09RevD.01\
State=1-A\HF=-900.1286375\RMSD=3.484e-09\RMSF=8.779e-07\Dipole=-2.4128
516,1.0202852,-0.1240266\Quadrupole=14.9232654,5.1392787,-20.0625442,0
.0463953,-1.1669249,1.6648412\PG=C01 [X(C19H20N3)]\\

3-(*cyclo*propyl)-1-phenyl-1,4-dihydrobenzo[*e*][1,2,4]triazin-4-yl (1m)

1\1\GINC-LOCALHOST\FOpt\UB3LYP\6-31G(2d,p)\C16H14N3(2)\PIOTR\27-Sep-20
22\0\\#P UB3LYP/6-31G(2d,p) FOpt(tight) SCF=Direct #P Geom=(NoDistance
,NoAngle) fcheck freq(noraman, readIso)\\1-Ph-Benzotriazinyl-3-cyclopr
opyl\\0,2\N,-0.4004095065,-0.2642099506,2.3563502573\N,0.6020232839,-0
.6740751532,1.5242476132\C,1.5549118146,0.2223225294,1.2622536463\N,1.
6427609583,1.4714175103,1.722237344\C,0.5809338104,1.9101912683,2.4624
231837\C,-0.5133339102,1.0576563705,2.7822563516\C,-1.6328863937,1.571
550103,3.4527485538\C,-1.6446768765,2.8995739777,3.8577747798\C,-0.551
2290017,3.7355029235,3.5963549915\C,0.5415103954,3.2467634582,2.902158
9502\C,-1.3255446004,-1.2939277114,2.6949024805\C,-1.7760816165,-2.157
8014824,1.6931127259\C,-2.6525427229,-3.1883400618,2.0143223156\C,-3.0
817624109,-3.3673194757,3.3291456609\C,-2.6177557848,-2.5126372852,4.3
273185432\C,-1.7366687258,-1.4800737072,4.0178488548\H,1.3914749747,3.
8738788083,2.6580947099\H,-0.5680111409,4.7691505104,3.9251749331\H,-2
.5143227507,3.2875173456,4.3772455892\H,-2.4888969255,0.9388527443,3.6
451733113\H,-1.3518323509,-0.8351414485,4.7985084588\H,-2.9287638901,-
2.6558068784,5.3568838817\H,-3.766433282,-4.1719159977,3.5751419531\H,
-3.0032387005,-3.8529812753,1.231649085\H,-1.4243832735,-2.01355872,0.
67962398\C,2.6278806923,-0.2730610327,0.3663607153\C,3.2676153101,0.68
04792467,-0.6278108102\C,4.0498375165,0.2205464606,0.5629791599\H,2.49
33105756,-1.3023078854,0.0574204136\H,2.8998169844,1.6998562065,-0.615
9271215\H,3.5172213077,0.2827587332,-1.605389943\H,4.8473186554,-0.500
3165401,0.420204773\H,4.1996482046,0.9349084391,1.3638880285\\Version=
ES64L-G09RevD.01\State=2-A\HF=-782.3535378\S2=0.764322\S2-1=0.\S2A=0.7
50135\RMSD=3.951e-09\RMSF=1.023e-06\Dipole=-0.8165081,-0.2785549,0.364
5013\Quadrupole=-1.0564745,0.9424419,0.1140326,1.6324774,-3.4181758,2.
2507905\PG=C01 [X(C16H14N3)]\\@

$1m^+$

1\1\GINC-LOCALHOST\FOpt\RB3LYP\6-31G(2d,p)\C16H14N3(1+)\PIOTR\19-Oct-2
022\0\\#P B3LYP/6-31G(2d,p) FOpt(tight) SCF=Direct #P Geom=(NoDistance
,NoAngle) fcheck freq(noraman, readIso)\\1-Ph-Benzotriazinyl-3-cyclopr
opyl cation\\1,1\N,-0.3753190078,-0.2561045297,2.3521288128\N,0.621541
095,-0.6728406427,1.6105873353\C,1.5799489217,0.2249842375,1.271951891
1\N,1.6039258407,1.4874352196,1.7001779256\C,0.5710175114,1.9175467219
,2.4290053464\C,-0.5207636737,1.0403000664,2.762036343\C,-1.6593327426
,1.5335619579,3.4429673457\C,-1.6710942945,2.8506682014,3.8261853089\C
,-0.571942305,3.7161367285,3.5581308856\C,0.5232950852,3.2673529097,2.
8720887924\C,-1.3264065514,-1.2937126121,2.6972652739\C,-1.8484738839,
-2.0756075397,1.6684755919\C,-2.7381448821,-3.0934995838,1.9928044223\
C,-3.0755446051,-3.3328453398,3.3255065783\C,-2.5241526438,-2.55455271
99,4.3434478797\C,-1.6443652359,-1.5212048427,4.0364401531\H,1.3619761
78,3.9073716392,2.6268597966\H,-0.621320092,4.747656289,3.8885504234\H
,-2.5405492153,3.2460123017,4.3393544124\H,-2.5036990239,0.888304825,3
.6397512482\H,-1.1871730283,-0.9314655657,4.8222349366\H,-2.7687652127
,-2.7562145021,5.3799472954\H,-3.7642341436,-4.1330935744,3.5721927412

\H,-3.1650862623,-3.7011587053,1.203336942\H,-1.5665129372,-1.87990626
1,0.6412594424\C,2.6452561308,-0.2877370567,0.4110789145\C,3.293454123
4,0.649584837,-0.6110485266\C,4.0698923287,0.2430147413,0.5874269931\H
,2.525691632,-1.3279938321,0.1378410752\H,2.9078815947,1.6620213988,-0
.6451861749\H,3.5318604174,0.2077769409,-1.571302525\H,4.8611699357,-0
.4896075795,0.481669791\H,4.2134595663,0.9772679012,1.3715106985\\Vers
ion=ES64L-G09RevD.01\State=1-A\HF=-782.1411276\RMSD=2.482e-09\RMSF=9.2
69e-07\Dipole=-0.7859535,0.5238813,0.5194635\Quadrupole=0.9902012,6.05
41269,-7.0443281,6.9254021,-11.7933094,4.4214892\PG=C01 [X(C16H14N3)]\

8. References

(1) Pomikło, D.; Bodzioch, A.; Pietrzak, A.; Kaszyński, P. *Org. Lett.* **2019**, *21*, 6995.

(2) Connelly, N. G.; Geiger, W. E. *Chem. Rev.* **1996**, *96*, 877.

(3) Neugebauer, F. A.; Rimmler, G. *Mag. Res. Chem.* **1988**, *26*, 595.

(4) Constantinides, C. P.; Obijalska, E.; Kaszyński, P. *Org. Lett.* **2016**, *18*, 916.

(5) Gaussian 09, Revision A.02, M. J. Frisch, G. W. Trucks, H. B. Schlegel, G. E. Scuseria, M. A. Robb, J. R. Cheeseman, G. Scalmani, V. Barone, B. Mennucci, G. A. Petersson, H. Nakatsuji, M. Caricato, X. Li, H. P. Hratchian, A. F. Izmaylov, J. Bloino, G. Zheng, J. L. Sonnenberg, M. Hada, M. Ehara, K. Toyota, R. Fukuda, J. Hasegawa, M. Ishida, T. Nakajima, Y. Honda, O. Kitao, H. Nakai, T. Vreven, J. A. Montgomery, Jr., J. E. Peralta, F. Ogliaro, M. Bearpark, J. J. Heyd, E. Brothers, K. N. Kudin, V. N. Staroverov, R. Kobayashi, J. Normand, K. Raghavachari, A. Rendell, J. C. Burant, S. S. Iyengar, J. Tomasi, M. Cossi, N. Rega, J. M. Millam, M. Klene, J. E. Knox, J. B. Cross, V. Bakken, C. Adamo, J. Jaramillo, R. Gomperts, R. E. Stratmann, O. Yazyev, A. J. Austin, R. Cammi, C. Pomelli, J. W. Ochterski, R. L. Martin, K. Morokuma, V. G. Zakrzewski, G. A. Voth, P. Salvador, J. J. Dannenberg, S. Dapprich, A. D. Daniels, O. Farkas, J. B. Foresman, J. V. Ortiz, J. Cioslowski, and D. J. Fox, Gaussian, Inc., Wallingford CT, 2009.

(6) Trasatti, S. *Pure Appl. Chem.* **1986**, *58*, 955.
